# Realization of a Heteroatom‐Transfer‐Ligand (HTL) Platform: Oxy Insertion at a Titanium–Alkyl Bond Facilitated by a Hydroxylaminato Ligand Framework

**DOI:** 10.1002/anie.202514536

**Published:** 2025-11-10

**Authors:** Thibault Cheisson, Henry H. Wilson, Michael R. Gau, Patrick J. Carroll, Eric J. Schelter

**Affiliations:** ^1^ Department of Chemistry University of Pennsylvania 231 S 34^th^ Street Philadelphia PA 19104 USA; ^2^ Department of Earth and Environmental Science University of Pennsylvania Philadelphia PA 19104 USA; ^3^ Department of Chemical and Biomolecular Engineering University of Pennsylvania Philadelphia PA 19104 USA; ^4^ Present address: Eramet, 10 boulevard de Grenelle Paris 75015 France

**Keywords:** Baeyer–Villiger type, Mechanism, Organometallics, Oxygenation, Titanium

## Abstract

Selective oxidation of metal‐carbon bonds is arguably one of the most important reactions in modern chemistry. In this work, we report the synthesis and the reactivity of a titanium alkyl complex [(TriNOx)Ti(CH_2_SiMe_3_)] (TriNOx^3−^ = [(2‐*^t^* BuNO)C_6_H_4_CH_2_]_3 _N)^3–^) that was found to undergo an internal rearrangement through an oxy‐insertion into the Ti─C bond facilitated by one of the hydroxylaminato moieties of the supporting TriNOx^3−^ ligand. Experimental and computational studies are in agreement with an organometallic Baeyer–Villiger‐type mechanism. Furthermore, stepwise dealkoxylation, reoxidation, and alkylation of the resulting complex allowed for closing a synthetic cycle and regenerating the initial starting complex. This work demonstrates the unique ability of the TriNOx^3−^ ligand to mediate and manage O‐atoms in the coordination sphere of a titanium cation and delineates a new type of metal‐ligand cooperativity through a Heteroatom‐Transfer Ligand (HTL) platform.

## Graphical abstract

A concept for carbon‐heteroatom bond functionalization: a Heteroatom‐Transfer Ligand (HTL) platform is demonstrated through oxygen insertion into a titanium‐carbon bond using a hydroxylamide ligand. A synthetic cycle for HTL is presented including kinetic and computational studies.

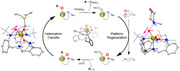

## Introduction

**Chart 1:**
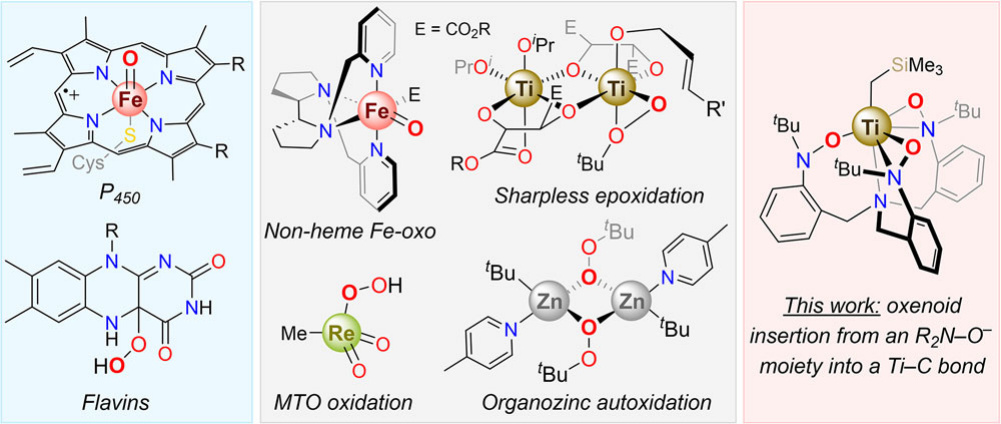
Natural or synthetic intermediates (isolated or proposed) responsible for oxygenation of organic substrates.^[^ ^2^, ^13^, ^14^, ^15^, ^16^, ^17^, ^18^, ^25^, ^26^ ^].^

The controlled functionalization of organic molecules with oxygen is one of the most important processes in biochemistry, fine chemical synthesis, and bulk chemical production.^[^ ^1^ ^]^ In Nature, such reactions are ubiquitous and accomplished by an array of oxygenases, relying on active motifs such as flavins, oxyferryl heme complexes (cytochromes P_450_), or nonheme oxo‐bridged bimetallic complexes.^[^ ^2^, ^3^, ^4^ ^]^ Biomimetic synthetic systems have been extensively identified and developed by chemists to accomplish these critical transformations.^[^ ^5^, ^6^, ^7^, ^8^, ^9^, ^10^, ^11^ ^]^ Among others (Chart 1) are: Ti‐based epoxidation,^[^ ^12^ ^]^ the methylrhenium trioxide (MTO) system,^[^ ^13^, ^14^, ^15^, ^16^, ^17^ ^]^ the nonheme Fe‐ and Mn‐oxo catalytic hydroxylation systems,^[^ ^18^, ^19^ ^]^ and the pioneering organozinc “auto‐oxidation” system reported by Frankland in 1849.^[^ ^20^ ^]^ Mimicking natural enzymatic pathways, these systems rely on the formation of reactive metal‐oxo fragments or the generation of activated peroxo moieties.

Molecular oxygen, hydrogen peroxide, and organic peroxides are the typical oxidants used in such metal‐mediated systems. However, the use of molecular oxygen with organic solvents or the use of peroxide can generally introduce high risk. For example, the primary oxidant for the Sharpless epoxidation, *^t^* BuOOH, is hazardous, with reports of explosions and fatalities associated with its use.^[^ ^21^ ^]^ Another example was seen in 2017 following hurricane Harvey, where flooding caused a failure of cooling systems used for organic peroxides and resulted in a fire at the Arkema plant in Crosby, Texas.^[^ ^22^ ^]^ The tendency of peroxides for energetic decomposition derives from their ability to easily generate free radicals and therefore to access (often nonselective) low energy radical pathways.^[^ ^23^ ^]^ For that reason, cooling is typically necessary to achieve selectivity and control in peroxide‐based oxidation reactions.^[^ ^8^, ^19^, ^24^ ^]^ Notably, these restrictions can become incompatible with thermally‐activated processes, such as C─H bond activation, hence limiting the development of (catalytic) hydroxylation procedures.

In this context, hydroxylaminato moieties (R_2_NO^−^) are structurally and electronically related to peroxo ligands (ROO^−^) and were identified early on as non‐reactive surrogates for the latter. Such ligands have helped in structural assignments of more reactive alkylperoxo‐species, as described by Wieghardt, Mimoun, or Sharpless for Mo‐, V‐, or Ti‐based epoxidation catalysts.^[^ ^27^, ^28^, ^29^ ^]^ While mimicking their coordination chemistry, hydroxylaminato ligands have typically failed at delivering electrophilic oxygen atom (oxenoid) equivalents that mirror the reactivity of organic peroxo moieties,^[^ ^8^, ^9^ ^]^ and have consequently been considered as chemically inert analogues.^[^ ^29^, ^30^, ^31^ ^]^

With our continuing interest in the coordination chemistry,^[^ ^32^, ^33^, ^34^, ^35^, ^36^, ^37^, ^38^, ^39^ ^]^ reactivity,^[^ ^40^, ^41^, ^42^, ^43^ ^]^ and application of hydroxylaminato ligands,^[^ ^44^, ^45^, ^46^, ^47^, ^48^ ^]^ we were curious to assess their proposed chemical inertness in oxygenation of organic moieties. In the present work, we report the synthesis and reactivity of a titanium alkyl complex [Ti(CH_2_SiMe_3_)TriNOx] supported by a tripodal hydroxylaminato ligand (TriNOx^3−^ = [(2‐*^t^* BuNO)C_6_H_4_CH_2_]_3 _N)^3–^)). We demonstrate the unprecedented ability of hydroxylaminato moieties to act as masked peroxides and to insert an oxenoid (oxy‐insertion) into a Ti─C bond in a transformation mimicking the reactivity of organic oxaziridines. Furthermore, we demonstrate that the partially deoxygenated complex can be reoxidized with a mild oxidant to close a synthetic cycle. In a broader perspective, these results showcase that hydroxylaminato ligands can store, shuttle, and regain oxygen atoms, creating a potential new case for metal‐ligand cooperativity, a concept that we refer to as a Heteroatom‐Transfer Ligand (HTL).

### Synthesis and Characterization

**Figure 1:**
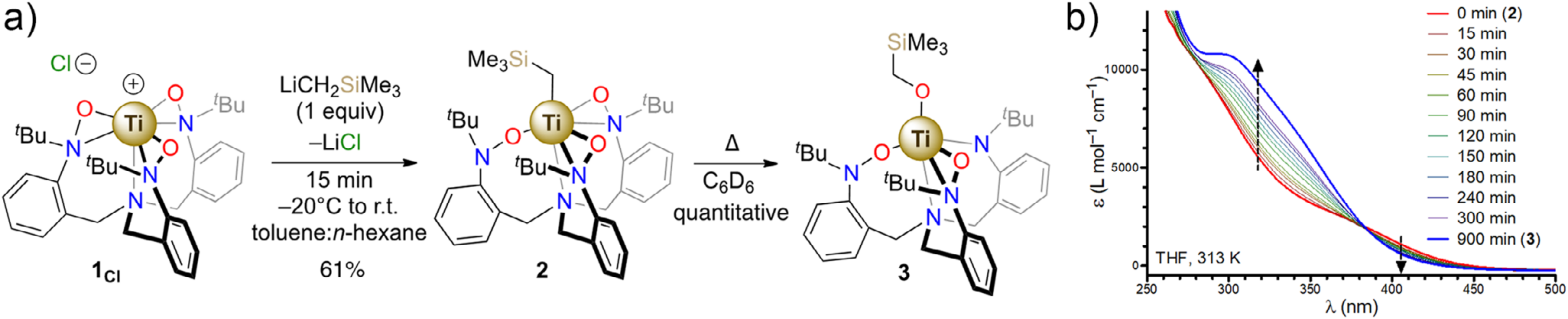
a) Synthesis of **2** and conversion to **3**. b) Conversion of **2** to **3** at 313 K in THF as observed by UV‐visible spectroscopy.

**Figure 2:**
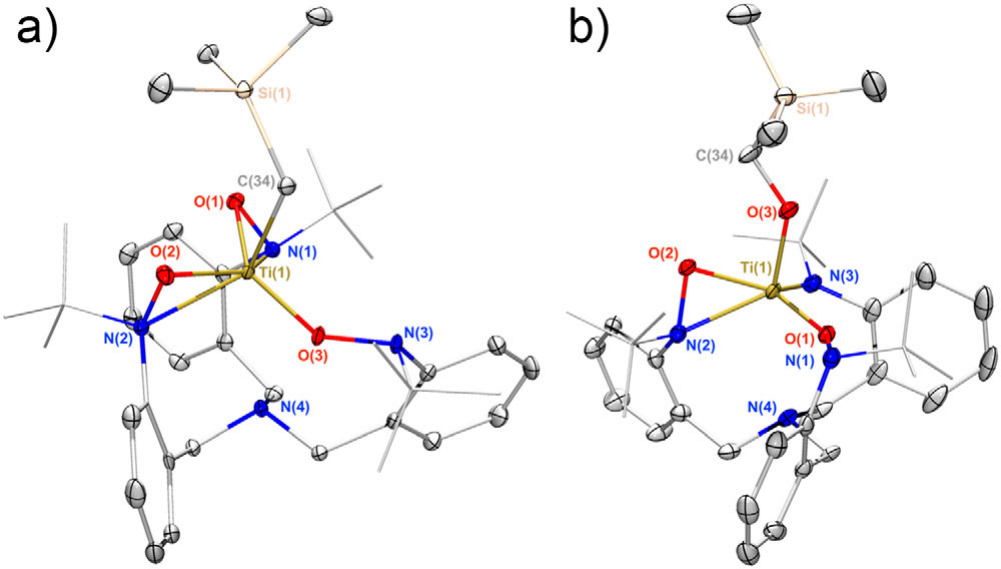
a) Thermal ellipsoid plot of **2**. b) Thermal ellipsoid plot of **3**, hydrogen atoms are omitted and *tert*‐butyl groups are depicted in a wireframe model for clarity.

Toward studies of oxygen atom insertion, alkylation of the previously synthesized [(TriNO*x*)Ti]Cl (**1_Cl_**),^[^ ^34^ ^]^ with LiCH_2_SiMe_3_ proceeds smoothly to provide [(TriNO*x*)Ti(CH_2_SiMe_3_)] (**2**) in 61% yield after filtration and recrystallization at −25 °C (Figure 1a), and a solid‐state crystallographic structure (Figure 2a) was obtained.

Notably, one hydroxylaminato arm is coordinated in κ^1^‐O fashion, while the other two are present in the typical κ^2^‐(*N*,*O*) binding mode. This ligand rearrangement is evidently necessary to accommodate the alkyl ancillary ligand in the Ti^4+^ cation coordination sphere, in contrast to f‐element TriNOx^3−^ complexes where such rearrangements are not observed.^[^ ^34^ ^]^ This asymmetry is not apparent in solution, however. ^1^H and ^13^C{^1^H} NMR spectroscopy of **2** (Figures S2 and S4) indicate typical *C_3_* symmetry, as observed for most TriNOx^3−^‐based complexes.^[^ ^35^, ^41^, ^44^, ^45^, ^49^, ^50^ ^]^ The coordination of the alkyl moieties to the Ti center is confirmed by the diastereotopic nature (δ_H_ = 2.34 and 1.38 ppm, |^2^ *J*_HH_| = 11.7 Hz, C_6_D_6_, 300 K) of the coordinated CH_2_SiMe_3_ protons, evidenced by ^1^H–^1^H COSY and ^13^C–^1^H HSQC correlation techniques (Figures S3 and S5). Lowering the temperature did not significantly alter the apparent symmetry of **2**; it is therefore surmised that the hydroxylaminato ligand rearrangement is dynamic and fast on the NMR timescale.

During an extended NMR spectroscopy investigation of isolated **2** at room temperature, we noted the appearance of a new set of signals in both the ^1^H‐ and ^13^C{^1^H} NMR spectra. Moderate heating of the C_6_D_6_ solution led to the quantitative conversion of **2** to the new species **3**. The newly formed complex **3** lacked any symmetry, with 3 sets of diastereotopic benzylic CH_2_ protons for the supporting ligand, 3 *^t^* Bu signals, and 1 signal for the ─SiMe_3_ moieties. The CH_2_ protons adjacent to the trimethylsilyl group are significantly deshielded compared to **2** and now appear as an AB quartet at 4.96 ppm (|^2^ *J*_HH_| = 14.2 Hz, C_6_D_6_, 300 K). This observation from the ^1^H‐NMR spectrum (Figure S8) was accompanied by full desymmetrization of the supporting ligand signals, evident by ^13^C{^1^H}‐NMR spectroscopy with 29 identifiable resonances consistent with *C*_1_ molecular symmetry for the complex (Figure S10). Finally, ^29^Si NMR showed some relative shielding of the trimethylsilyl group at δ_Si_ = 0.0 ppm in **2** versus δ_Si_ = −2.3 ppm in **3** (Figures S7 and S13). Notably, visible light was not observed to impact the transformation of **2** to **3**. Taken together, these data are consistent with a thermally driven internal rearrangement of **2** having a major impact on the observed solution symmetry. To gain more information, single crystals were grown by slow evaporation to determine the exact structure of **3** (Figure 2b), which was revealed as the product of a single oxygen‐atom transfer from one of the hydroxylaminato groups into the Ti─C bond.

Consistent with the solution data, the titanium center in the X‐ray structure of **3** lies in an asymmetrical environment with one hydroxylaminato arm coordinating in a κ^1^‐O fashion, the second in a κ^2^‐(*N*,*O*) coordination mode, and the ligand's third arm binding as an amide moiety. The Ti(IV) cation's coordination sphere is completed by the newly‐formed alkoxide ligand. These modifications to the Ti(IV) environment impact the vibrational and electronic characteristics of **3** compared to **2**, as reflected in their UV–vis spectra. As such, the transformation of the intensely yellow colored **2** into the faint yellow **3** (Figure S24) was monitored in THF at 313 K and demonstrated the conversion of **2** to **3** with an isosbestic point at 382 nm (Figure 1b). To more closely study the kinetics of this transformation, we turned our attention to ^1^H‐NMR spectroscopy at variable temperature.

**Figure 3:**
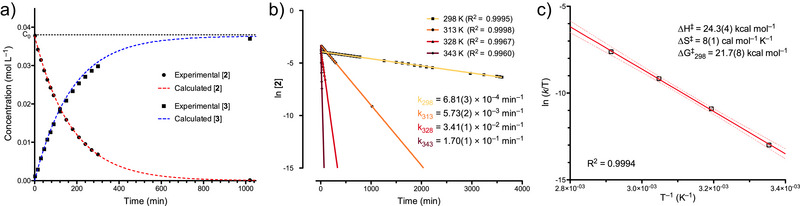
a) Experimental conversion of **2** to **3** against time at 313 K and associated first order kinetics fitted for c_0_ = 38.7 mmol L^−1^ and *k_313_* = 5.73 × 10^−3^ min^−1^. b) Linearized conversion of **2** against time and associated rate constants at 298, 313, 328, and 343 K. c) Eyring‐Polanyi plot with associated 95% confidence interval band and resulting activation parameters.

Conversions of C_6_D_6_ solutions of **2** were monitored against a ferrocene internal standard at different temperatures (Figure 3a). Consistent with UV–vis absorption data, all experiments are cleanly modeled by a first order rate law, with rate constant *k_T_* at a given temperature:

$$-\frac{d\left[2\right]}{dt}=k_{T}\;\left[2\right]=\frac{d\left[3\right]}{dt}$$

Rate constants were obtained at 298, 313, 328, and 343 K (Figure 3b). Application of the Eyring‐Polanyi formalism allowed extraction of activation parameters associated with the conversion of **2** to **3**: ∆H^‡^ = 24.3(4) kcal mol^−1^ and ∆S^‡^ = 8(1) cal mol^−1^ K^−1^ (Figure 3c). The free energy of activation at 298 K ∆G^‡^_298_ = 21.7(8) kcal mol^−1^ agrees well with the observed slow transformation at room temperature, while the small entropy of activation is consistent with an intramolecular rearrangement described by a first‐order rate law.

Given the synthetic interest of the observed oxy‐insertion, we were interested in studying the ability of **3** to release the formed alkoxide moiety and regenerate the original TriNOx^3−^ ligand to close a synthetic cycle. Treatment of **3** with electron‐poor or ‐rich boranes such as B(C_6_F_5_)_3_ or BEt_3_ indicated no reaction at room temperature and led to intractable decomposition at elevated temperatures. On the other hand, treatment of **3** with an excess of the silicon‐based electrophile,^[^ ^51^ ^]^ SiMe_3_Cl, led to the appearance of a new product **4_Cl_** characterized by an unsymmetrical ^1^H NMR spectrum similar to **3** (Figure S30). This result suggested the substitution of the alkoxide ligand by a chloride in the Ti coordination sphere, which was confirmed by X‐ray crystallographic analysis (Figure S40).

**Scheme 1:**
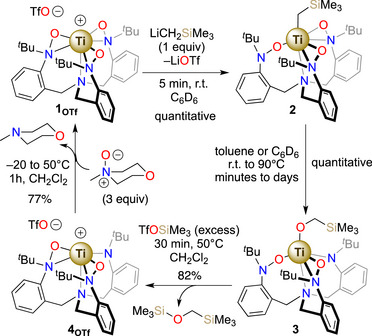
Synthetic cycle encompassing the oxy‐insertion rearrangement, dealkoxylation, and re‐oxidation steps.

**Figure 4:**
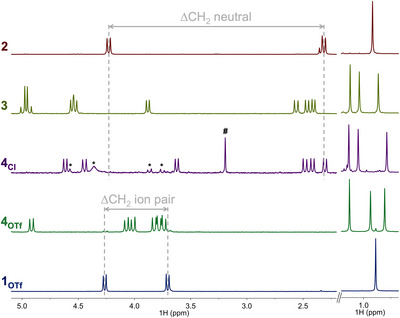
Benzylic and *tert*‐butyl regions in the ^1^H NMR (400 MHz) spectra of **2**, **3**, **4_Cl_** in C_6_D_6_ and **4_OTf_**, **1_OTf_** in CD_2_Cl_2_. The “diastereotopic gap” (Δδ_dia_) for neutral and ion pair compounds is illustrated. * denotes an unidentified impurity and # denotes the SiMe_3_OCH_2_SiMe_3_ side‐product. See the Supporting Information for the full spectra.

The conversion of **3** to **4_Cl_** proved to be slow and was therefore not pursued but, the result encouraged us to explore the reactivity of **3** with a more potent electrophile. Treatment of a toluene or benzene solution of **3** by a slight excess of SiMe_3_OTf led to an immediate color change from yellow to pale pink, followed by the deposition of a microcrystalline pink solid **4_OTf_**. After heating at 323 K for 30 minutes, **4_OTf_** was filtered and isolated in 82% yield (Scheme 1). Notably, the silyl ether Me_3_SiOCH_2_SiMe_3_ was identified from the filtrate. The isolated solid **4_OTf_** was dissolved in CD_2_Cl_2_ and demonstrated, again, an unsymmetrical species as determined by ^1^H and ^13^C{^1^H} NMR spectroscopy (Figures S14 and S16). The presence of the triflate anion was ascertained by ^19^F{^1^H} and ^13^C{^1^H} NMR spectroscopy (Figure S19). Contrary to **2**, **3**, **4_Cl_** for which large differences in the ^1^H chemical shifts of the diastereotopic benzylic ligand protons are observed (Figure 4, ∆δ_dia_ > 1.2 ppm); the diastereotopic benzylic proton NMR signals in **4_OTf_** were found in a much narrower range (∆δ_dia_ < 0.35 ppm) and downfield (Figure 4). As previously observed for TriNOx‐based Ce^IV^ and Ti^IV^ complexes,^[^ ^34^, ^52^ ^]^ this small “diastereotopic gap” is characteristic of the absence of an apical ligand on the cationic metal center and contraction of the metal coordination sphere. Although X‐ray quality crystals could not be obtained for **4_OTf_**, this observation strongly supports the formation of a charge‐separated complex with a cationic titanium center and an outer sphere triflate counter anion as depicted in Scheme 1.

**Figure 5:**
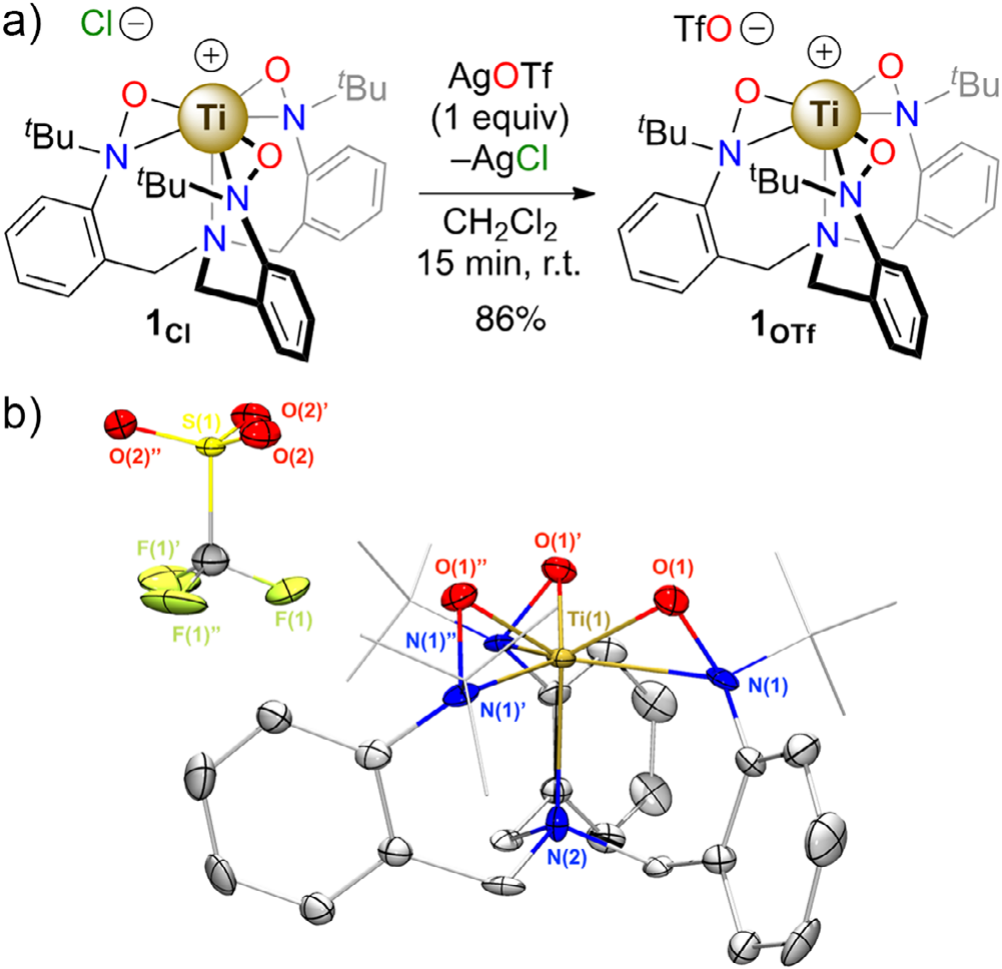
a) Independent synthesis of **1_OTf_**. b) Thermal ellipsoid plot of **1_OTf_**, hydrogen atoms are omitted for clarity and *tert*‐butyl groups are depicted in a wireframe model for clarity.

In order to close a synthetic cycle, the next step was the reoxidation of **4_OTf_** by an oxygen‐atom transfer (OAT) reagent. Toward this goal, we focused our attention on *N*‐oxides due to their well‐known ability to deliver O‐atoms.^[^ ^53^ ^]^ Heating a mixture of pyridine *N*‐oxide and **4_OTf_** at reflux in CD_2_Cl_2_ demonstrated the formation of a *C_3_*‐symmetrical minor product, later identified as the reoxidized complex **1_OTf_** (vide infra); however, the reaction was not clean with several as yet unidentified side‐products present in the mixture. Hypothesizing that we would observe cleaner reactivity with tertiary alkyl amine *N*‐oxides, trimethylamine *N*‐oxide and *N*‐methylmorpholine *N*‐oxide (NMO) were screened. Both reagents efficiently reoxidize the ligand framework to produce **1_OTf_** in nearly quantitative yield as shown by ^1^H NMR spectroscopy monitoring (Figures S32–S34). On a preparative scale, a 3‐fold excess of NMO reacted with **4_OTf_** afforded **1_OTf_** in a 77% isolated yield. **1_OTf_** was identified on the basis of its ^1^H NMR spectrum that is nearly identical to **1_Cl_** leading to the conclusion that **1_OTf_** is an ion pair in solution with a [TriNO*x*(Ti)]^+^ cation and a triflate counter‐ion. This result was confirmed by an independent synthesis through AgOTf salt‐metathesis from **1_Cl_** and an associated crystallographic structure confirming that the supported assignment of the solution speciation (Figure 5).

Finally, NMR‐scale experiments demonstrated that **1_OTf_** can be alkylated with LiCH_2_SiMe_3_ to regenerate **2** (Figure S35). This sequence of reactions forms a closed synthetic cycle for the transformation of alkyl lithium to their respective silyl ether. While the synthetic interest of the said transformation is modest, the mode of functionalization of a metal‐alkyl described here is highly unusual and opens a fundamentally new way of oxidizing alkyl fragments.

### ^15^N NMR spectroscopy

**Figure 6:**
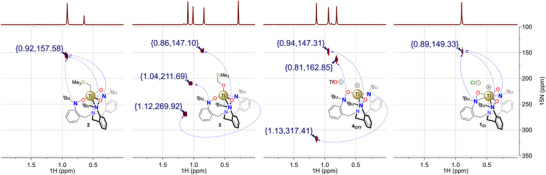
^1^H‐^15^N HMBC NMR spectra of (left to right) **2**, **3**, **4_OTf,_** and **1_Cl_** at 300 K in C_6_D_6_ (**2** and **3**) or CD_2_Cl_2_ (**4_OTf_** and **1_Cl_**) and cross‐peak assignments.

The series of complexes produced in this study also afforded a unique occasion to probe and correlate the different coordination modes for the hydroxylaminato and amide moieties to their respective ^15^N NMR chemical shifts and validate the solution‐based structures of these different complexes. ^1^H–^15^N heteronuclear long‐range correlation NMR experiments afforded — in a reasonable time at natural abundance — good cross peak correlations between the protons of the *tert*‐butyl moieties and the nitrogen atom bearing them (Figure 6).

The comparison of the spectra of **2**, **3**, **4_OTf,_** and **1_Cl_** enabled assignment of the resonance at δ_N_ ∼ 148 ppm to the κ^2^‐(*N*,*O*) coordination mode observed in all complexes reported in this study. For **3**, δ_N_ = 212 ppm was assigned to the κ^1^‐(O) coordination of the hydroxylaminato moieties observed in the solid‐state (Figure 2b), and finally, δ_N_ = 270 ppm was assigned to the coordinated amide moieties.

For **4_OTf_**, the solution ^15^N data support three distinct nitrogen coordination modes: κ^2^‐(N,O) at δ_N_ = 147 ppm, κ^1^‐(O) at δ_N_ = 163 ppm and κ^1^‐(N) at 313 ppm. The chemical shift differences observed between **3** and **4_OTf_** are best explained by the cationic nature of the latter complex. Related studies on TriNO*x*‐based Al, Ti, Ce, and Dy complexes^[^ ^34^, ^45^, ^52^, ^54^ ^]^ have demonstrated that the absence of an apical ligand causes “sinking” of the metal center into the TriNOx^3−^ pocket due to enhanced interaction with the bridgehead tertiary amine: for example Ti(1)−N(4) 3.065(1) Å in **2** versus Ti(1)−N(2) 2.302(5) Å in **1_OTf_**. From these observations, we propose that the coordination of the nitrogen at δ_N_ = 163 ppm in **4_OTf_** has a coordination mode intermediate between pure κ^2^‐(*N*,*O*) (as in **1_Cl_**) and pure κ^1^‐(O) (as in **3**).

For **2**, it is noteworthy that solid‐state data indicated a single κ^1^‐(O) coordination mode, while ^1^H and ^13^C{^1^H} agreed with a *C*_3_‐symmetric species on the NMR timescale, suggesting a dynamic behavior in solution. This hypothesis is confirmed by the observation of a single cross peak at δ_N_ = 158 ppm, i.e., deshielded by 10 ppm when compared with other κ^2^‐(*N*,*O*) nitrogen chemical shifts. This change in δ_N_ is assumed to arise from an averaged contribution of a κ^1^‐(O) coordination mode.

In summary, the ^15^N NMR spectroscopic data presented in this study contribute to a better knowledge of hydroxylaminato complexes spectroscopic properties but most importantly create a connection between solid‐ and solution‐state data by providing a useful probe to monitor hydroxylaminato ligands’ coordination modes and their ability to react in an oxy‐insertion process.

### DFT Studies

**Figure 7:**
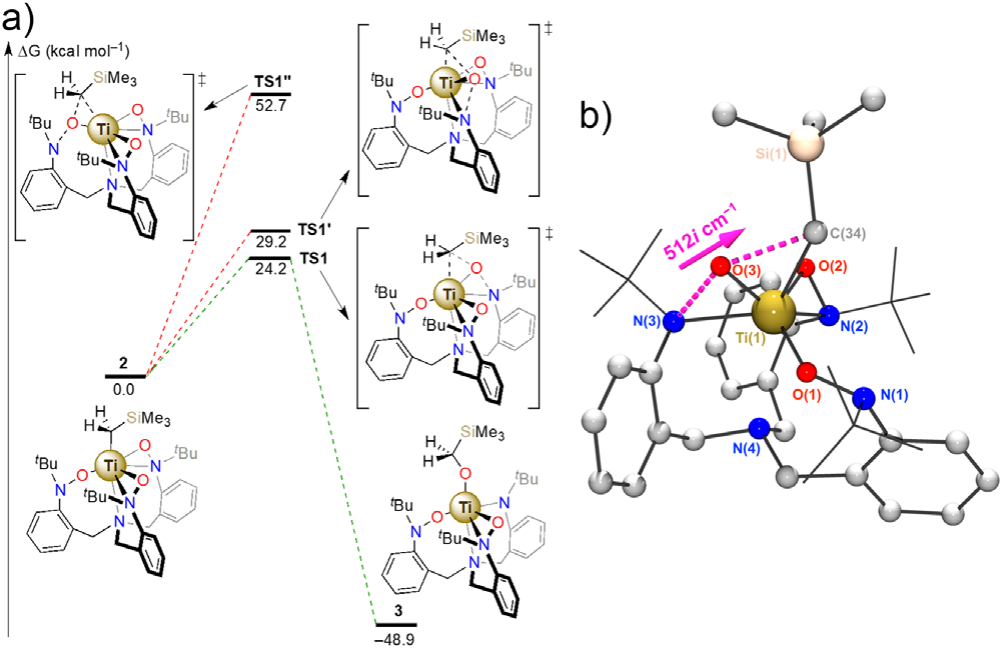
a) DFT‐optimized reaction coordinate for the transformation of **2** to **3**. b) Depiction of the DFT‐optimized **TS1** and main displacement vector associated with the imaginary frequency. Selected bond lengths [Å] and angles [°]: N(3)−O(3) 1.941, O(3)−C(34) 2.186, C(34)−Ti(1) 2.379, Ti(1)−N(3) 2.198; N(3)−O(3)−C(34) 146.9, C(34)−Ti(1)−N(3) 119.6.

We next turned our attention to DFT methods to study in more detail the mechanism of the observed oxy‐insertion reaction of **2** and the ligand reoxidation steps. Starting from the optimized structure of **2,** we evaluated independently the ability of each hydroxylaminato moiety to insert an oxenoid into the Ti–C bond. Transition states were located for these three trajectories (Figure 7a). All demonstrated a Ti‐mediated transfer of an oxygen atom to the CH_2_SiMe_3_ moiety, synchronous with the cleavage of the Ti─C bond—no intermediate metal‐oxo structure was implicated along the reaction coordinate. Energetically, the κ^2^‐(*N*,*O*) coordination mode is more favorable to the oxy‐insertion process than the κ^1^‐*O* coordination mode, with **TS1** and **TS1′** located at 24 and 29 kcal mol^−1^ over **2** and **TS1″** about 53 kcal mol^−1^ over **2,** respectively. **TS1″** consists of a Ti(1)–O(1)–C(34) metallocycle, while **TS1** and **TS1′** are characterized by a 4‐membered metallacycle geometry arising from the initial κ^2^‐(N,O) coordination mode. In **TS1**, the elements of the cycle are nearly coplanar with a N(3)–O(3)–Ti(1)–C(34) torsion angle of 178° (Figure 7b). Conversely, in **TS1′** the four atoms deviate significantly from co‐planarity with a torsion angle at 155° due to less favorable steric interactions. A control experiment performed between only NMO and LiCH_2_Si(CH_3_)_3_ notably showed no evidence for the oxy‐insertion product, instead producing tetramethylsilane, presumably through acid‐base chemistry as generally observed in the literature.^[^ ^55^, ^56^, ^57^, ^58^ ^]^ This result indicates the essential requirement for the metal complex to observe the oxy‐insertion chemistry and provides supporting evidence for the geometric characteristics that lower the relative energy of **TS1**.

**Figure 8:**
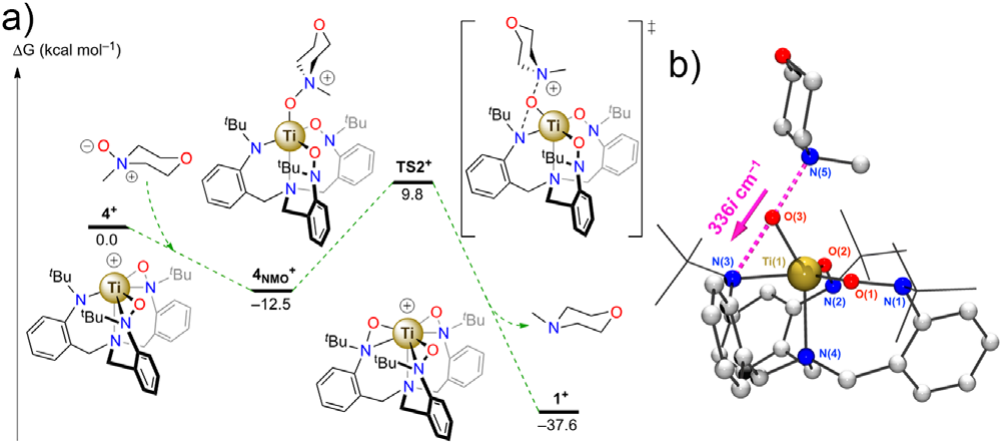
a) DFT‐optimized reaction coordinate for the transformation of **4^+^** to **1^+^**. b) Depiction of the DFT‐optimized **TS2^+^** and main displacement vector associated with the imaginary frequency. Selected bond lengths [Å] and angles [°]: N(3)−O(3) 2.0157, O(3)−N(5) 1.970, O(3)−Ti(1) 1.873; N(3)−O(3)−N(5) 177.4.

Following the synthetic cycle, we next examined the reoxidation step. Starting from the optimized cationic structure, **4^+^**, the coordination of NMO was found to be exergonic by 12.5 kcal mol^−1^ and induced a reorganization of the coordination sphere with the two hydroxylaminato arms switching to the κ^1^‐*O* coordination mode. A transition state for the transfer of the oxygen atom O(3) from N(5) to N(3), **TS2^+^**, was located at 9.8 kcal mol^−1^ above **4^+^**, corresponding to a free energy barrier of 22.3 kcal mol^−1^ for the reoxidation process from **4_NMO_^+^** (Figure 8a). Similar to **TS1**, the O‐atom transfer is mediated by the Ti center with the four atoms engaged in the transition state essentially coplanar with a N(3)–O(3)–Ti(1)–N(5) torsion angle at 177°, suggesting directionality to the underlying orbital interactions (Figure 8b). Following the intrinsic reaction coordinate from **TS2^+^** leads to a restored complex **1^+^** and free *N*‐methylmorpholine with no observed interaction between these two products. The entire reoxidation process of **4^+^** to **1^+^** by NMMO is notably exergonic by ∼38 kcal mol^−1^.

Overall, the computational investigation of the synthetic cycle demonstrates a good agreement between the calculated barrier and the experimental observations. The transition state for the oxy‐insertion **TS1**, found at 24.2 kcal mol^−1^, is in good agreement with the experimentally determined barrier of 21.7(8) kcal mol^−1^. Similarly, the experimentally facile reoxidation of **4_OTf_** by NMO was found to require ∼22 kcal mol^−1^ by DFT methods. Both the O‐atom transfer and reoxidation processes feature synchronous bond‐breaking and ‐forming events mediated by the Ti‐center, without metal‐oxo intermediates. Indeed, both transition states are fundamentally similar in their optimized structures, and their minimum energies tend toward a linear N–O–N interaction between the constituents of the transition state and co‐planar with the Ti(IV) center. Finally, the κ^2^‐(*N*,*O*) coordination mode of the hydroxylaminato moieties appears to be instrumental in achieving the low‐energy thermal pathway by facilitating approach to transition state geometries for both O‐atom transfer steps, in agreement with Hammond's postulate.

## Discussion

**Figure 9:**
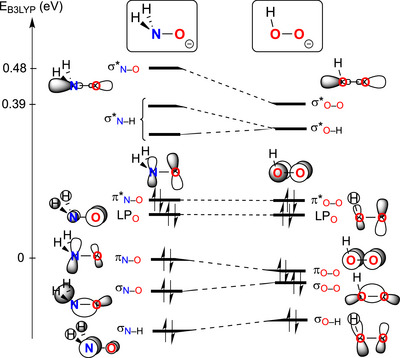
Comparative molecular orbital diagram of H_2_NO^−^ and HOO^−^ (B3‐LYP/6–31G*).

In the present work, we have demonstrated the ability of κ^2^‐(*N*,*O*)‐bound hydroxylaminato moieties to deliver an oxenoid fragment that can be inserted in a Ti─C bond. To the best of our knowledge, such behavior is unprecedented, but reminiscent of the reactivity of peroxides. A comparison of scaled, simplified molecular orbitals diagrams for H_2_NO^–^ and HOO^–^ (B3LYP/6–31G*) demonstrates the similarities between the two fragments while indicating the general lack of reactivity of hydroxylaminato moieties compared to peroxides (Figure 9).

In H_2_NO^–^, the σ*_N–O_ is notably ∼0.09 eV higher in energy than the corresponding σ*_O–O_ in HOO^–^, caused by the electronegativity difference in the hydroxlyaminato fragment versus the peroxide. This can be understood to hinder nucleophilic attack on hydroxlyaminato moieties due to poor orbital energy overlap, whereas a more favorable situation for the peroxide electrophile is encountered, evidenced by their increased reactivity.

Coordination to a metal center and substitution are likely to alter this simplified interpretation of the differences between hydroxylaminato and hydroperoxide; however, it is empirically established that coordinated hydroxylaminato complexes are generally reported to be stable in a wide array of conditions, while peroxide complexes are often unstable and reactive.^[^ ^29^, ^30^, ^31^ ^]^

It is proposed that the tethered TriNOx^3–^ ligand framework leads to sufficient alignment of orbital energy levels and hydroxylaminato orientation to partly promote N─O bond cleavage by the nucleophilic alkyl moiety of **2** while helping to stabilize the σ*_N–O_ orbitals by creating a near linear geometry, favorable for insertion of the oxygen atom into the Ti─C bond through **TS1**. This important templating role of the TriNOx^3–^ HTL platform can also be rationalized by noting that previous alkyl‐titanium(IV)‐hydroxylaminato complexes described by Mitzel & Waymouth were stable at elevated temperatures (100 °C) or in the presence of a strongly alkylating agent (AlMe_3_).^[^ ^59^, ^60^ ^]^

**Figure 10:**
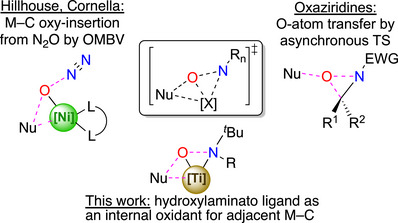
Comparison of the reported system with Organometallic Baeyer–Villiger (OMBV) oxidations at Ni centers,^[^ ^61^, ^62^, ^63^, ^64^ ^]^ and with electron deficient oxaziridines.^[^ ^65^ ^].^

Overall, the reactivity of this HTL system and the mechanistic elements studied are reminiscent of two types of described systems (Figure 10): *O*‐atom transfer reactions from organic oxazirdines and oxy‐insertion at an M─C bond by N_2_O through an organometallic Baeyer–Villiger oxidation.

In the reported oxaziridine systems, substitution at the nitrogen atom by electron‐withdrawing groups allows for *O*‐atom transfer upon reaction with a variety of nucleophiles.^[^ ^65^, ^66^, ^67^ ^]^ This *O*‐atom transfer is performed through a concerted, asynchronous, nearly linear transition state that resembles **TS1**, based on mechanistic investigation by Beak and coworkers^[^ ^68^ ^]^ and computations by Houk et al.^[^ ^69^ ^]^

On the other hand, Hillhouse and co‐workers have described oxy‐insertion at alkyl‐ and aryl‐Hf and Ni complexes upon reaction with N_2_O,^[^ ^61^, ^62^, ^70^, ^71^ ^]^ through an organometallic Baeyer–Villiger‐type (OMBV) mechanism initially established for methylrhenium trioxide oxidation (MTO, Chart 1).^[^ ^17^, ^72^, ^73^, ^74^, ^75^, ^76^, ^77^, ^78^ ^]^ This chemistry was revisited and expanded by Cornella and co‐workers, who demonstrated catalytic production of phenols and substituted alcohols from arylhalides using nitrous oxide (N_2_O) as the oxidant. These transformations are thought to be facilitated by the oxy‐insertion of N_2_O into a Ni^I^─C bond by an OMBV‐type mechanism.^[^ ^63^, ^64^ ^]^ Formal O‐atom insertion into metal‐alkyls have also been reported to proceed through different mechanisms involving acid‐base chemistry and rearrangement of the O‐atom transfer agent as for example reported by Arnold when reacting a thorium‐alkyl species with trimethylamine N‐oxide.^[^ ^79^ ^]^

One of the unique features of the present HTL system lies in the nature of the oxenoid moiety (a hydroxylaminato ligand), and the fact that it does not generate a leaving group as in oxaziridine chemistry (an imine) or OMBV chemistry (N_2_, H_2_O, PhI, IO_3_^–^, etc.). In the present case, a coordinated amide moiety remains in the coordination sphere of the Ti(IV) center, allowing for the regeneration of the active HTL platform, evidenced by the reactivity of **4_OTf_**. When the deoxygenated complex is made cationic and electrodeficient, with a sterically exposed titanium center, the complex **4_OTf_** is prone to regain electron density by incorporating an oxygen atom through an oxenoid insertion into the nucleophilic Ti─N bond, regenerating the TriNO*x* ^3–^ HTL in the process.

This relatively facile transformation between the hydroxylaminato form and deoxygenated amide form of the TriNOx^3–^ ligand mediated by oxenoid insertion is remarkable and constitutes an interesting case of metal‐ligand cooperativity based on heteroatom transfer. Redox active‐ and proton‐based cooperative ligands are widely‐known and have resulted in many important catalytic outcomes.^[^ ^80^, ^81^, ^82^, ^83^ ^]^ The Heteroatom‐Transfer Ligand (HTL), TriNO*x* ^3−^, reported herein contributes a new class of cooperative oxygen‐atom mediator, with strong synthetic potential given the importance of oxygenation reactions in modern chemistry.

## Conclusion

In conclusion, we have explored the reactivity of a unique alkyl‐titanium(IV) complex supported by a tethered hydroxylaminato ligand ([Ti(CH_2_SiMe_3_)TriNOx]). Upon thermal activation, an oxy‐insertion into the Ti^IV^─C bond from one of the hydroxylaminato arms was observed, turning the latter into an amide moiety. Experimental and computational mechanistic studies point toward an intramolecular, closed‐shell, concerted mechanism by an organometallic Baeyer–Villiger‐type mechanism without an intermediate oxo complex. Furthermore, dealkoxylation of the resulting complex allowed for the generation of a cationic Ti^IV^ complex that was observed to react quantitatively with tertiary amine *N*‐oxides to regenerate the original TriNOx^3–^ framework. Finally, alkylation of this complex allowed the closure of a synthetic cycle for the oxidation of the alkyl moieties by [Ti(TriNOx)]^+^. Although the current system has no practical synthetic or catalytic applications in the present form, it demonstrates a unique mode of reactivity that is reminiscent and complementary to recent reports by Cornella on catalytic oxidation of arylhalides.^[^ ^63^, ^64^ ^]^ Moreover, this system demonstrates the versatility of the TriNOx^3–^ ligand platform that allow for controllable and reversible O‐atom storage and delivery into key molecular bonds. We contend that this ligand‐assisted management of oxygen atoms may be a new case of metal‐ligand‐metal cooperativity that affords articulation of a heteroatom‐transfer ligand (HTL) concept. Further exploration and development of this concept, including expansion of initial results that show similar reactivity with [Th(CH_2_SiMe_3_)TriNOx]^[^ ^49^ ^]^ albeit at slower rates, is underway in our laboratory.

## Conflict of Interests

The authors declare no conflict of interest.

## Supporting information



Supporting Information

## Data Availability

The data that support the findings of this study are openly available in the Cambridge Structural Database at https://www.ccdc.cam.ac.uk, Deposition numbers 2469559–2469562, and in the ESI.

## References

[1] G. M. Tomboc , Y. Park , K. Lee , K. Jin , Chem. Sci. 2021, 12, 8967–8995, 10.1039/D1SC01272J. 34276926 PMC8261717

[2] J. C. Lewis , P. S. Coelho , F. H. Arnold , Chem. Soc. Rev. 2011, 40, 2003–2021, 10.1039/C0CS00067A. 21079862 PMC3064445

[3] J. Dong , E. Fernández‐Fueyo , F. Hollmann , C. E. Paul , M. Pesic , S. Schmidt , Y. Wang , S. Younes , W. Zhang , Angew. Chem. Int. Ed. 2018, 57, 9238–9261, 10.1002/anie.201800343. PMC6099261 29573076

[4] Y. Liang , J. Wei , X. Qiu , N. Jiao , Chem. Rev. 2018, 118, 4912–4945, 10.1021/acs.chemrev.7b00193. 29630343

[5] D. C. Powers , T. Ritter , in Comprehensive Organic Synthesis II *(Second Edition)* (Ed.: P. Knochel ), Elsevier, Amsterdam, 2014, pp. 719–743.

[6] V. Dantignana , A. Company , M. Costas , Isr. J. Chem. 2020, 60, 1004–1018.

[7] S. S. Stahl , J. A. Labinger , J. E. Bercaw , Angew. Chem. Int. Ed. 1998, 37, 2180–2192, 10.1002/(SICI)1521-3773(19980904)37:16<2180::AID-ANIE2180>3.0.CO;2-A. 29711451

[8] Y. Minko , I. Marek , Org. Biomol. Chem. 2014, 12, 1535–1546, 10.1039/C3OB42349B. 24477293

[9] G. Boche , J. C. W. Lohrenz , Chem. Rev. 2001, 101, 697–756, 10.1021/cr940260x. 11712501

[10] A. V. Sberegaeva , D. Watts , A. N. Vedernikov , in Advances in Organometallic Chemistry, Vol. 67 (Ed.: P. J. Pérez ), Academic Press, Cambridge, MA, 2017, pp. 221–297;

[11] W. Lu , L. Zhou , Oxidation of C‐H Bonds, Wiley, Hoboken, NJ, 2017, 10.1002/9781119092490.

[12] R. A. Johnson , K. B. Sharpless , in Catalytic Asymmetric Synthesis (Ed.: I. Ojima ), Wiley, New York, 2005, 229–280.

[13] W. A. Herrmann , F. E. Kühn , Acc. Chem. Res. 1997, 30, 169–180, 10.1021/ar9601398.

[14] C. C. Romão , F. E. Kühn , W. A. Herrmann , Chem. Rev. 1997, 97, 3197–3246, 10.1021/cr9703212. 11851489

[15] W. A. Herrmann , R. W. Fischer , D. W. Marz , Angew. Chem. Int. Ed. 1991, 30, 1638–1641, 10.1002/anie.199116381.

[16] F. E. Kühn , A. Scherbaum , W. A. Herrmann , J. Organomet. Chem. 2004, 689, 4149–4164.

[17] J. M. Gonzales , R. Distasio , R. A. Periana , W. A. Goddard , J. Oxgaard , J. Am. Chem. Soc. 2007, 129, 15794–15804, 10.1021/ja0714742. 18052160

[18] M. C. White , J. Zhao , J. Am. Chem. Soc. 2018, 140, 13988–14009, 10.1021/jacs.8b05195. 30185033 PMC6410715

[19] M. Milan , M. Salamone , M. Costas , M. Bietti , Acc. Chem. Res. 2018, 51, 1984–1995, 10.1021/acs.accounts.8b00231. 30080039

[20] D. Seyferth , Organometallics 2001, 20, 2940–2955, 10.1021/om010439f.

[21] B. Li , S. M. Guinness , S. Hoagland , M. Fichtner , H. Kim , S. Li , R. J. Maguire , J. C. McWilliams , J. Mustakis , J. Raggon , D. Campos , C. R. Voss , E. Sohodski , B. Feyock , H. Murnen , M. Gonzalez , M. Johnson , J. Lu , X. Feng , X. Sun , S. Zheng , B. Wu , Org. Process Res. Dev. 2018, 22, 707–720, 10.1021/acs.oprd.8b00083.

[22] Organic Peroxide Decomposition, Release, and Fire at Arkema Crosby Following Hurricane Harvey Flooding, U.S. Chemical Safety and Hazard Investigation Board, 2018, 2017‐08‐I‐TX.

[23] P. Molyneux , Tetrahedron 1966, 22, 2929–2943, 10.1016/S0040-4020(01)82271-7.

[24] S. T. Oyama , Mechanisms in Homogeneous and Heterogeneous Epoxidation Catalysis, Elsevier, Amsterdam, 2008.

[25] I. D. Williams , S. F. Pedersen , K. B. Sharpless , S. J. Lippard , J. Am. Chem. Soc. 1984, 106, 6430–6431, 10.1021/ja00333a060.

[26] J. Lewiński , W. Śliwiński , M. Dranka , I. Justyniak , J. Lipkowski , Angew. Chem. Int. Ed. 2006, 45, 4826–4829. 10.1002/anie.200601001 16795096

[27] K. Wieghardt , W. Holzbach , E. Hofer , J. Weiss , Inorg. Chem. 1981, 20, 343–348, 10.1021/ic50216a007.

[28] S. F. Pedersen , J. C. Dewan , R. R. Eckman , K. B. Sharpless , J. Am. Chem. Soc. 1987, 109, 1279–1282, 10.1021/ja00238a065.

[29] L. Saussine , H. Mimoun , A. Mitschler , J. Fischer , New J. Chem. 1980, 4, 235.

[30] L. H. Doerrer , J. R. Galsworthy , M. L. H. Green , M. A. Leech , M. Müller , J. Chem. Soc., Dalton Trans. 1998, 19, 3191–3194, 10.1039/a803126f.

[31] D. C. Crans , J. J. Smee , E. G. Gaidamauskiene , O. P. Anderson , S. M. Miller , W. Jin , E. Gaidamauskas , E. Crubellier , R. Grainda , L.‐H. Chi , G. R. Willsky , J. Inorg. Biochem. 2004, 98, 1837–1850, 10.1016/j.jinorgbio.2004.08.010. 15522411

[32] A. McSkimming , J. Su , T. Cheisson , M. R. Gau , P. J. Carroll , E. R. Batista , P. Yang , E. J. Schelter , Inorg. Chem. 2018, 57, 4387–4394, 10.1021/acs.inorgchem.7b03238. 29569906

[33] J. A. Bogart , A. J. Lewis , S. A. Medling , N. A. Piro , P. J. Carroll , C. H. Booth , E. J. Schelter , Inorg. Chem. 2013, 52, 11600–11607, 10.1021/ic401974t. 24024698

[34] M. A. Boreen , J. A. Bogart , P. J. Carroll , E. J. Schelter , Inorg. Chem. 2015, 54, 9588–9593, 10.1021/acs.inorgchem.5b01687. 26397706

[35] T. Cheisson , L. A. Solola , M. R. Gau , P. J. Carroll , E. J. Schelter , Organometallics 2018, 37, 4332–4335, 10.1021/acs.organomet.8b00366.

[36] E. N. Lapsheva , T. Cheisson , C. Álvarez Lamsfus , P. J. Carroll , M. R. Gau , L. Maron , E. J. Schelter , Chem. Commun. 2020, 56, 4781—4784. 10.1039/c9cc10052k 32226992

[37] J. A. Bogart , C. A. Lippincott , P. J. Carroll , E. J. Schelter , Angew. Chem. Int. Ed. 2015, 54, 8222–8225, 10.1002/anie.201501659. 26014901

[38] J. Su , T. Cheisson , A. McSkimming , C. A. P. Goodwin , I. M. DiMucci , T. Albrecht‐Schönzart , B. L. Scott , E. R. Batista , A. J. Gaunt , S. A. Kozimor , P. Yang , E. J. Schelter , Chem. Sci. 2021, 12, 13343–13359, 10.1039/D1SC03905A. 34777753 PMC8528073

[39] R. F. Higgins , K. P. Ruoff , A. Kumar , E. J. Schelter , Acc. Chem. Res. 2022, 55, 2616–2627, 10.1021/acs.accounts.2c00312. 36041177

[40] H. Fang , B. E. Cole , Y. Qiao , J. A. Bogart , T. Cheisson , B. C. Manor , P. J. Carroll , E. J. Schelter , Angew. Chem. Int. Ed. 2017, 56, 13450–13454, 10.1002/anie.201706894. 28777883

[41] B. E. Cole , T. Cheisson , R. F. Higgins , E. Nakamaru‐Ogiso , B. C. Manor , P. J. Carroll , E. J. Schelter , Inorg. Chem. 2020, 59, 172–178, 10.1021/acs.inorgchem.9b00975. 31199139

[42] J. E. Kim , J. A. Bogart , P. J. Carroll , E. J. Schelter , Inorg. Chem. 2016, 55, 775–784, 10.1021/acs.inorgchem.5b02236. 26689656

[43] A. B. Weberg , S. Chaudhuri , T. Cheisson , C. Uruburo , E. Lapsheva , P. Pandey , M. R. Gau , P. J. Carroll , G. C. Schatz , E. J. Schelter , Chem. Sci. 2022, 13, 6796–6805, 10.1039/D2SC01926D. 35774165 PMC9200122

[44] B. E. Cole , I. B. Falcones , T. Cheisson , B. Manor , P. Carroll , E. J. Schelter , Chem. Commun. 2018, 54, 10276–10279, 10.1039/C8CC04409K. 30140822

[45] T. Cheisson , B. E. Cole , B. C. Manor , P. J. Carroll , E. J. Schelter , Acs Sustain Chem Eng 2019, 7, 4993–5001, 10.1021/acssuschemeng.8b05638.

[46] T. Cheisson , J. Jian , J. Su , T. M. Eaton , M. R. Gau , P. J. Carroll , E. R. Batista , P. Yang , J. K. Gibson , E. J. Schelter , Phys. Chem. Chem. Phys., 2019, 21, 19868–19878, 10.1039/C9CP03764K. 31475264

[47] R. F. Higgins , T. Cheisson , B. E. Cole , B. C. Manor , P. J. Carroll , E. J. Schelter , Angew. Chem. Int. Ed. 2020, 59, 1851–1856, 10.1002/anie.201911606. 31610094

[48] J. A. Bogart , B. E. Cole , M. A. Boreen , C. A. Lippincott , B. C. Manor , P. J. Carroll , E. J. Schelter , Proc. Natl. Acad. Sci. USA 2016, 113, 14887–14892, 10.1073/pnas.1612628113. 27956636 PMC5206573

[49] T. Cheisson , K. D. Kersey , N. Mahieu , A. McSkimming , M. R. Gau , P. J. Carroll , E. J. Schelter , J. Am. Chem. Soc. 2019, 141, 9185–9190, 10.1021/jacs.9b04061. 31117665

[50] B. E. Cole , T. Cheisson , J. J. M. Nelson , R. F. Higgins , M. R. Gau , P. J. Carroll , E. J. Schelter , Acs Sustain Chem Eng 2020, 8, 14786–14794, 10.1021/acssuschemeng.0c03974.

[51] C. A. Malapit , M. Borrell , M. W. Milbauer , C. E. Brigham , M. S. Sanford , J. Am. Chem. Soc. 2020, 142, 5918–5923, 10.1021/jacs.9b13531. 32207616 PMC7533108

[52] J. A. Bogart , C. A. Lippincott , P. J. Carroll , C. H. Booth , E. J. Schelter , Chemistry – A European Journal 2015, 21, 17850–17859, 10.1002/chem.201502952. 26503580

[53] E. A. LaPierre , M. L. Clapson , W. E. Piers , L. Maron , D. M. Spasyuk , C. Gendy , Inorg. Chem. 2018, 57, 495–506, 10.1021/acs.inorgchem.7b02766. 29260872

[54] A. J. Woodside , M. A. Smith , T. M. Herb , B. C. Manor , P. J. Carroll , P. R. Rablen , C. R. Graves , Organometallics 2019, 38, 1017–1020.

[55] R. Beugelmans , L. Benadjila‐Iguertsira , J. Chastanet , G. Negron , G. Roussi , Can. J. Chem. 1985, 63, 725–734, 10.1139/v85-120.

[56] R. Beugelmans , L. Benadjilaiguertsira , G. Roussi , J Chem Soc Chem Comm 1982, 10, 544, 10.1039/c39820000544.

[57] M. J. Neal , E. J. Chartier , A. M. Lane , S. L. Hejnosz , L. T. Jesikiewicz , P. Liu , J. J. Rohde , P. Lummis , D. J. Fox , J. D. Evanseck , T. D. Montgomery , J. Org. Chem. 2025, 90, 3673–3683, 10.1021/acs.joc.4c03090. 40083234 PMC11915385

[58] M. J. Neal , S. L. Hejnosz , J. J. Rohde , J. D. Evanseck , T. D. Montgomery , J. Org. Chem. 2021, 86, 11502–11518, 10.1021/acs.joc.1c01047. 34379424

[59] A. Willner , J. Niemeyer , N. W. Mitzel , Dalton Trans. 2009, 23, 4473–4480, 10.1039/b821786f. 19488445

[60] A. P. Dove , X. Xie , R. M. Waymouth , Chem. Commun. 2005, 16, 2152–2154, 10.1039/b418778d. 15846430

[61] P. T. Matsunaga , G. L. Hillhouse , A. L. Rheingold , J. Am. Chem. Soc. 1993, 115, 2075–2077, 10.1021/ja00058a085.

[62] K. Koo , G. L. Hillhouse , A. L. Rheingold , Organometallics 1995, 14, 456–460, 10.1021/om00001a062.

[63] F. L.e Vaillant , A. M. Calbet , S. González‐Pelayo , E. J. Reijerse , S. Ni , J. Busch , J. Cornella , Nature 2022, 604, 677–683, 10.1038/s41586-022-04516-4. 35478236 PMC9046086

[64] S. Ni , F. L. Vaillant , A. Mateos‐Calbet , R. Martin , J. Cornella , J. Am. Chem. Soc. 2022, 144, 18223–18228, 10.1021/jacs.2c06227. 36162124 PMC9562464

[65] K. S. Williamson , D. J. Michaelis , T. P. Yoon , Chem. Rev. 2014, 114, 8016–8036, 10.1021/cr400611n. 24754443 PMC4150611

[66] F. A. Davis , A. C. Sheppard , Tetrahedron 1989, 45, 5703–5742, 10.1016/S0040-4020(01)89102-X.

[67] F. A. Davis , Tetrahedron 2018, 74, 3198–3214, 10.1016/j.tet.2018.02.029.

[68] D. R. Anderson , K. W. Woods , P. Beak , Org. Lett. 1999, 1, 1415–1417, 10.1021/ol990969w.

[69] K. N. Houk , J. Liu , N. C. DeMello , K. R. Condroski , J. Am. Chem. Soc. 1997, 119, 10147–10152, 10.1021/ja963847x.

[70] G. A. Vaughan , P. B. Rupert , G. L. Hillhouse , J. Am. Chem. Soc. 1987, 109, 5538–5539, 10.1021/ja00252a047.

[71] P. T. Matsunaga , J. C. Mavropoulos , G. L. Hillhouse , Polyhedron 1995, 14, 175–185, 10.1016/0277-5387(94)00330-H.

[72] R. A. Periana , D. J. Taube , S. Gamble , H. Taube , T. Satoh , H. Fujii , Science 1998, 280, 560–564, 10.1126/science.280.5363.560. 9554841

[73] S. M. Bischof , M.‐J. Cheng , R. J. Nielsen , T. B. Gunnoe , W. A. Goddard , III, R. A. Periana , Organometallics 2011, 30, 2079–2082, 10.1021/om2002365.

[74] S. N. Brown , J. M. Mayer , J. Am. Chem. Soc. 1996, 118, 12119–12133, 10.1021/ja962087n.

[75] T. M. Figg , T. R. Cundari , T. B. Gunnoe , Organometallics 2011, 30, 3779–3785, 10.1021/om200258d.

[76] T. M. Figg , J. R. Webb , T. R. Cundari , T. B. Gunnoe , J. Am. Chem. Soc. 2012, 134, 2332–2339, 10.1021/ja2102778. 22188276

[77] T. M. Figg , T. R. Cundari , Organometallics 2012, 31, 4998–5004, 10.1021/om300270x.

[78] D. B. Pardue , J. Mei , T. R. Cundari , T. B. Gunnoe , Inorg. Chem. 2014, 53, 2968–2975, 10.1021/ic402759w. 24571202

[79] N. S. Settineri , M. E. Garner , J. Arnold , J. Am. Chem. Soc. 2017, 139, 6261–6269, 10.1021/jacs.7b02356. 28430420

[80] C. Gunanathan , D. Milstein , Acc. Chem. Res. 2011, 44, 588–602, 10.1021/ar2000265. 21739968

[81] A. Kumar , P. Daw , D. Milstein , Chem. Rev. 2022, 122, 385–441, 10.1021/acs.chemrev.1c00412. 34727501 PMC8759071

[82] O. R. Luca , R. H. Crabtree , Chem. Soc. Rev. 2013, 42, 1440–1459, 10.1039/C2CS35228A. 22975722

[83] P. J. Chirik , Acc. Chem. Res. 2015, 48, 1687–1695, 10.1021/acs.accounts.5b00134. 26042837

