# Supplementary material for: Realization of a Heteroatom‐Transfer‐Ligand (HTL) Platform: Oxy Insertion at a Titanium–Alkyl Bond Facilitated by a Hydroxylaminato Ligand Framework
Source: Angew Chem Int Ed Engl. 2025 Nov 10;64(52):e14536. doi: 10.1002/anie.202514536 (PMC12723462; doi:10.1002/anie.202514536)
Supplement: Supplementary file 1 — Supporting Information [file ANIE-64-e14536-s001.docx]

Supporting Information for:

**Realization of a Heteroatom-Transfer-Ligand (HTL) Platform: Oxy Insertion at a Titanium--Alkyl Bond Facilitated by a Hydroxylaminato Ligand Framework**

Thibault Cheisson,*^[a,b]^ Henry H. Wilson,^[a]^ Michael R. Gau,^[a]^ Patrick J. Carroll,^[a]^ and Eric J. Schelter*^[a]^

^[a]^ Department of Chemistry, University of Pennsylvania, 231 S. 34^th^ St., Philadelphia, PA 19104, U.S.A.

^[b]^ Present address: Eramet, 10 Boulevard de Grenelle, 75015 Paris, France.

^[c]^ Department of Earth and Environmental Science, University of Pennsylvania, Philadelphia, PA 19104, U.S.A.

^[d]^ Department of Chemical and Biomolecular Engineering, University of Pennsylvania, Philadelphia, PA 19104, U.S.A.

*E-mails: thibault.cheisson@eramet.com, schelter@sas.upenn.edu

**Table of contents.**

**General considerations** **S2**

**Materials** **S2**

**Synthetic and Characterization Details** **S3**

**UV-Vis Spectroscopy**  **S17**

**Kinetic Experiments** **S18**

**NMR-Scale Reactivity Studies** **S21**

**X-ray crystallography** **S27**

**Computational Details** **S30**

**References** **S40**

**General considerations.**

All reactions and manipulations were performed under an inert N_2_ atmosphere using standard Schlenk techniques or in a drybox (Vacuum Atmospheres Co.) equipped with a molecular sieves 13X/Q5 Cu–0226S catalyst purifier system. Glassware was oven-dried for at least 3 h at 150 °C prior to use. Nuclear magnetic resonance (NMR) spectra: ^1^H, ^1^H–^1^H COSY, ^13^C{^1^H}, ^13^C–^1^H HSQC, ^15^N–^1^H HMBC, ^1­9^F{^1^H} and ^29^Si–^1^H INEPT were collected on a Bruker AVIII-400 Fourier transform NMR spectrometer at 298K. Chemical shifts are reported in parts per million (ppm) and referenced against characteristic solvent peaks (^1^H and ^13^C) or appropriate external standards (^15^N, ^19^F and ^29^Si). The following abbreviations are used; b, broad; s, singlet; d, doublet; t, triplet. Elemental analyses were performed on a Costech ECX 4010 Analyzer.

**Materials.**

Benzene, dichloromethane (DCM), diethyl ether (Et_2_O), ­*n*-hexane, *n*-pentane, tetrahydrofuran (THF) and toluene were dried using a commercial two-column Q5 reactant- and neutral alumina-packed system and stored over 3Å molecular sieves. Hexamethyldisiloxane (TMS_2_O) was distilled over calcium hydride and stored over 3Å molecular sieves. DCM-d_2_ and C_6_D_6_ (Cambridge Isotopes) were dried over 3Å molecular sieves prior to use. H_3_TriNOx was prepared as previously described.^1^ (Trimethylsilyl)methyllithium solution (1.0M in pentane) (Sigma) was dried and recrystallized from *n*-hexane prior to use. (Chlorotriisopropyxtitanium(IV) (Sigma), trimethylsilyl trifluoromethanesulfonate (Sigma), silver trifluoromethanesulfonate (Sigma) and *N*-methylmorpholine *N*-oxide (Sigma) were used as received.

**Synthetic and Characterization Details.**

| **[Ti(TriNOx)]Cl (1_Cl_).** This compound was previously prepared from H_3_TriNOx, TiCl_4_(THF)_2_, and NEt_3_ in 53% yield.^2^ An alternative synthesis from TiCl(O*^i^*Pr)_3_ was developed for the present report: H_3_TriNOx (1.11 g, 2.02 mmol) and TiCl(O*^i^*Pr)_3_ (525 mg, 2.01 mmol) were dissolved in benzene (50 mL) in a Schlenk flask. The reaction mixture was heated to 50 °C for 16 h without stirring providing a yellow solution and pale yellow crystalline material. The mixture was filtered and washed successively with benzene (3×3 mL) and Et_2_O (2×5 mL). The solid was dried under reduced pressure to provide [TiCl(TriNOx)] (**1_Cl_**) as pale-yellow crystalline flakes (1.10 g, 1.74 mmol, 86%). |  |
| --- | --- |
| ^15^N–^1^H HMBC NMR (CD_2_Cl_2_, 40.5 MHz, 300 K) δ 149.3 (*N*O(*^t^*Bu)).  Other spectroscopic data were identical to literature.^2^  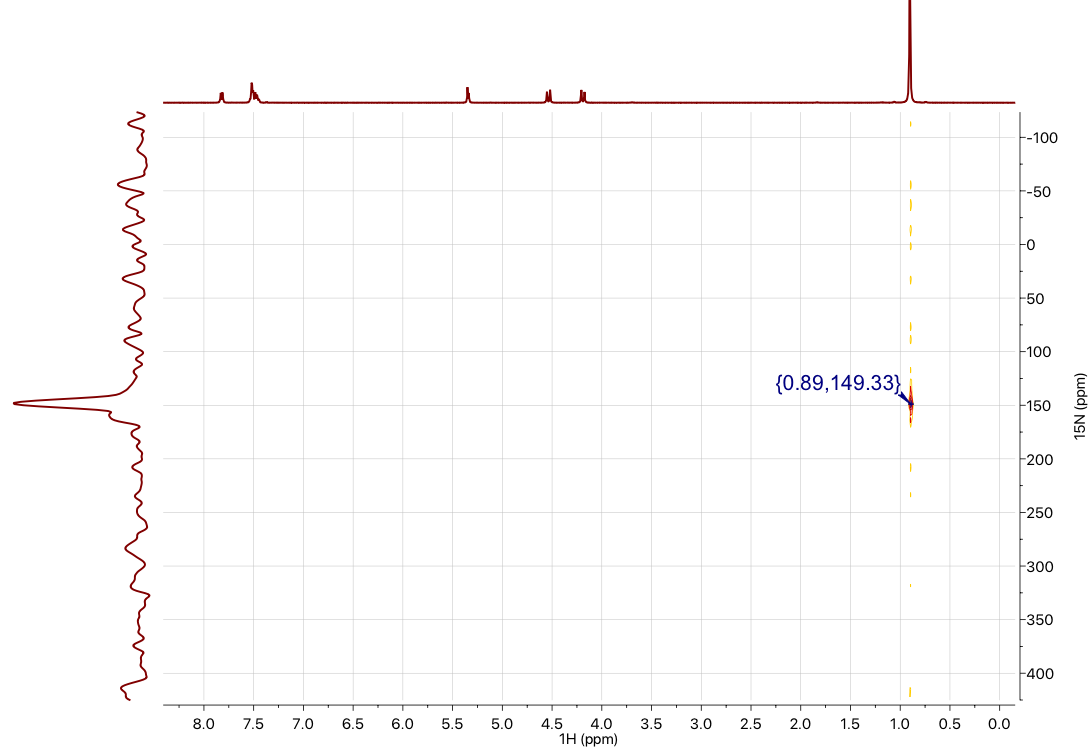  Figure S ^15^N–^1^H HMBC NMR (CD_2_Cl_2_, 40.5 MHz, 300 K) spectrum of 1_Cl_. | |
| **[Ti(CH_2_SiMe_3_)(TriNOx)] (2).** [Ti(TriNOx)]Cl (87.4 mg, 0.14 mmol) was suspended in toluene (2 mL). In a separate vial, LiCH_2_SiMe_3_ (13.1 mg, 0.14 mmol, 1 equiv) was dissolved in *n*-hexane (3 mL). The two solutions were cooled down to −25 °C for 1 hour. The solution of LiCH_2_SiMe_3_ was added dropwise to the stirring solution of [Ti(TriNOx)]Cl over 2 minutes. Over the course of the addition, the mixture turned yellow and clear. The mixture was stirred at room temperature for 15 minutes leading to a bright yellow solution and a fine white precipitate of LiCl. The mixture was filtered and washed with *n*-hexane (2 mL) and stored at −25 °C. After one day, a first batch of yellow crystals was collected and dried (42.8 mg, 63 µmol, 45%). The mother liquor volume was concentrated to ~2 mL followed by overnight storage at −25°C to afford a second batch of crystals (15.2 mg, 22 µmol, 61% total yield). |  |

^1^H NMR (C_6_D_6_, 400 MHz, 300 K) δ 7.62 (d, ^3^*J*_HH_= 8.0 Hz, 3H, Ar*H*), 7.06 (t, ^3^*J*_HH_= 8.0 Hz, 3H, Ar*H*), 7.00–6.87 (m, 6H, Ar*H*), 4.23 (d, ^2^*J*_HH_= 11.8 Hz, 3H, NC*H*_2_), 2.34 (d, ^2^*J*_HH_= 11.7 Hz, 1H, C*H*_2_Si), 2.32 (s, ^2^*J*_HH_= 11.8 Hz, 3H, NC*H*_2_), 1.38 (s, ^2^*J*_HH_= 11.7 Hz, 1H, C*H*_2_Si), 0.92 (s, 27H, C(C*H*_3_)_3_), 0.64 (s, 9H, Si(C*H*_3_)_3_).

^13^C{^1^H} NMR (C_6_D_6_, 125.7 MHz, 300 K) δ 149.8 (s, Ar), 133.8 (s, Ar), 133.1 (s, Ar), 129.2 (s, Ar), 127.6 (s, Ar), 125.8 (s, Ar), 71.2 (s, *C*H_2_Si), 64.8 (s, *C*(CH_3_)_3_), 60.2 (s, N*C*H_2_), 27.3 (s, C(*C*H_3_)_3_), 3.5 (s, Si(*C*H_3_)_3_).

^15^N–^1^H HMBC NMR (C_6_D_6_, 40.5 MHz, 300 K) δ 157.6 (*N*O(*^t^*Bu)).

^29^Si–^1^H INEPT NMR (C_6_D_6_, 79.5 MHz, 300 K) δ −0.03 (s, CH_2_*Si*Me_3_).

Anal. Cald. for C_37_H_56_N_4_O_3_SiTi: C, 65.27; H, 8.29; N, 8.23. Found: C, 65.59; H, 8.03; N, 8.44.


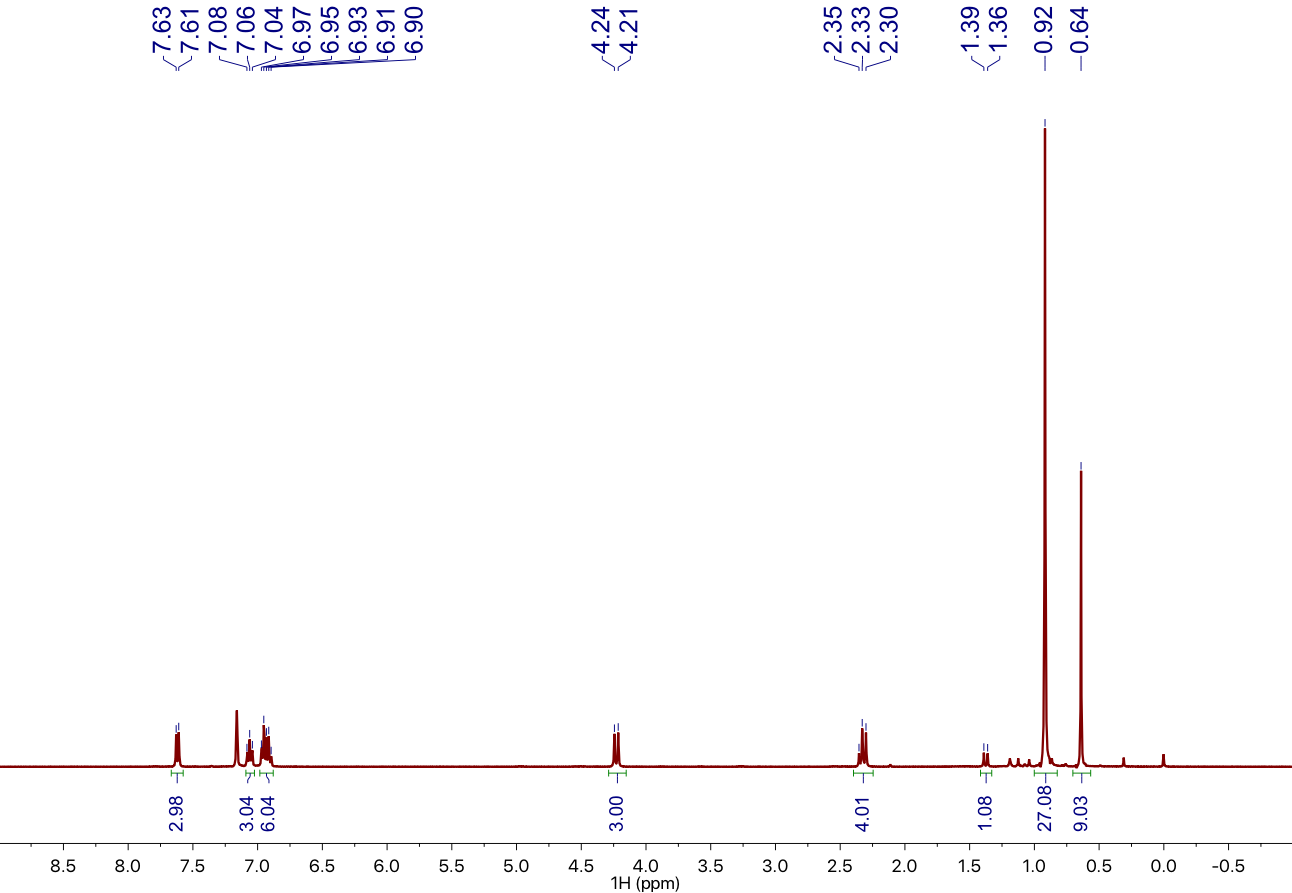


Figure S ^1^H NMR (C_6_D_6_, 400 MHz, 300 K) spectrum of 2.


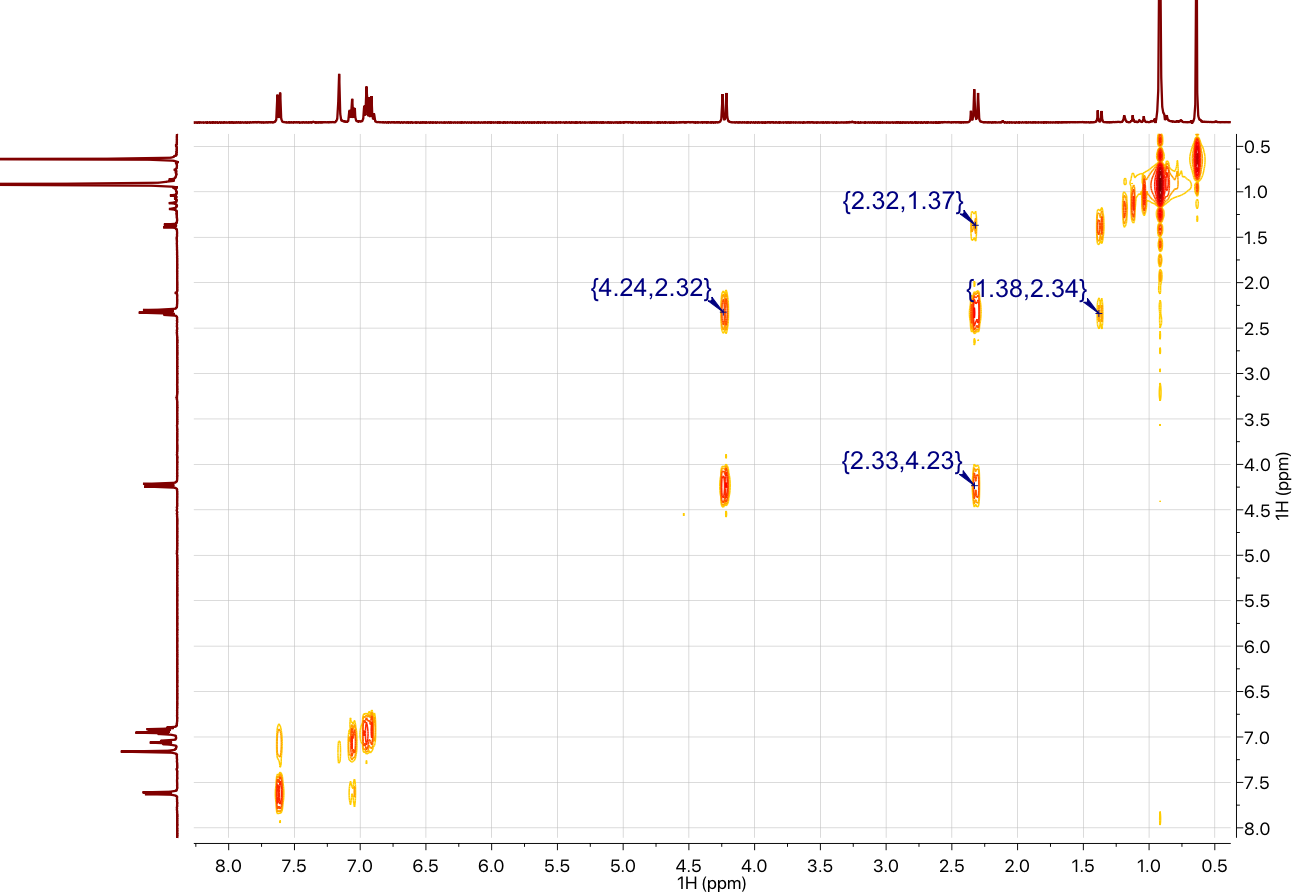


Figure S ^1^H–^1^H COSY NMR (C_6_D_6_, 500 MHz, 300 K) spectrum of 2.


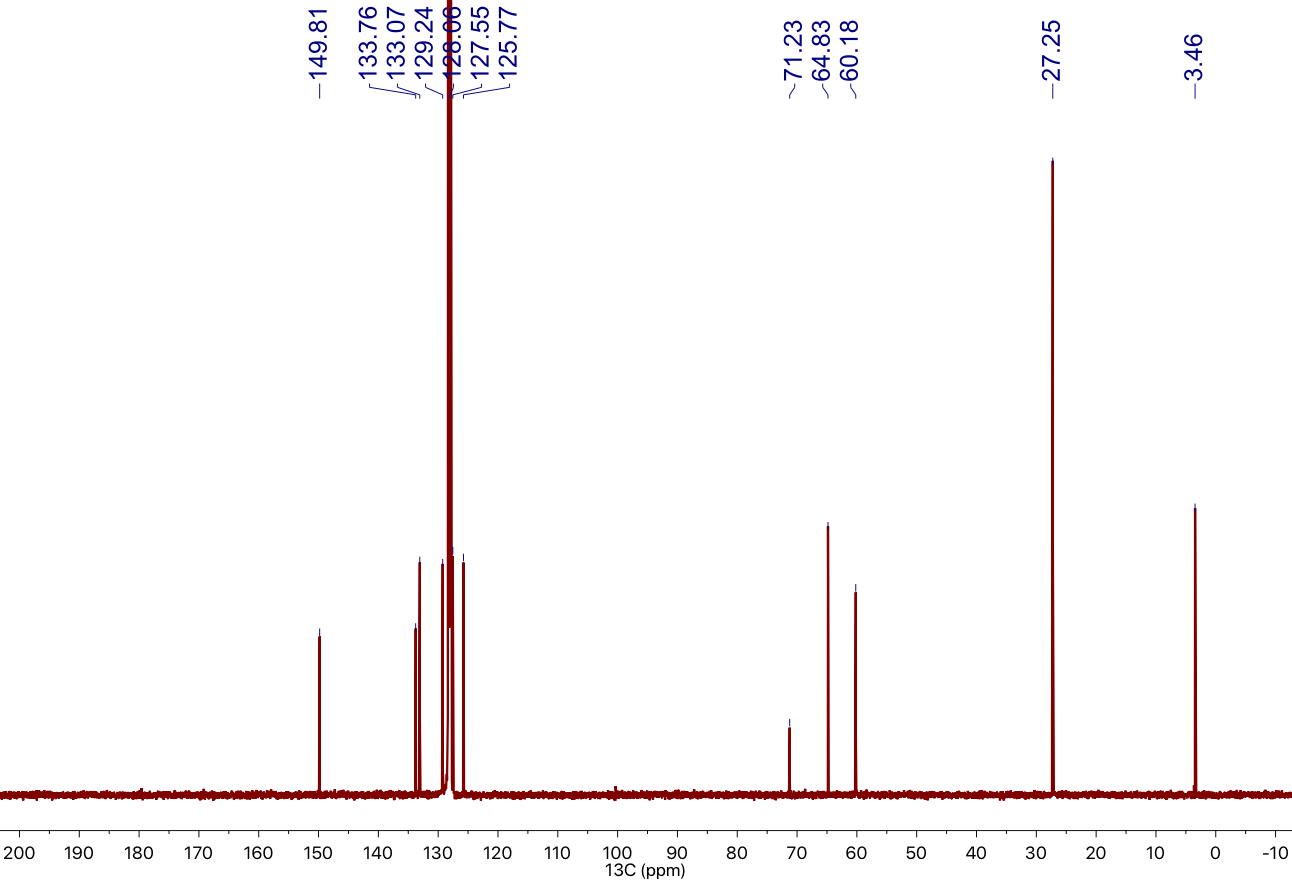


Figure S ^13^C{^1^H} NMR (C_6_D_6_, 125.7 MHz, 300 K) spectrum of 2.


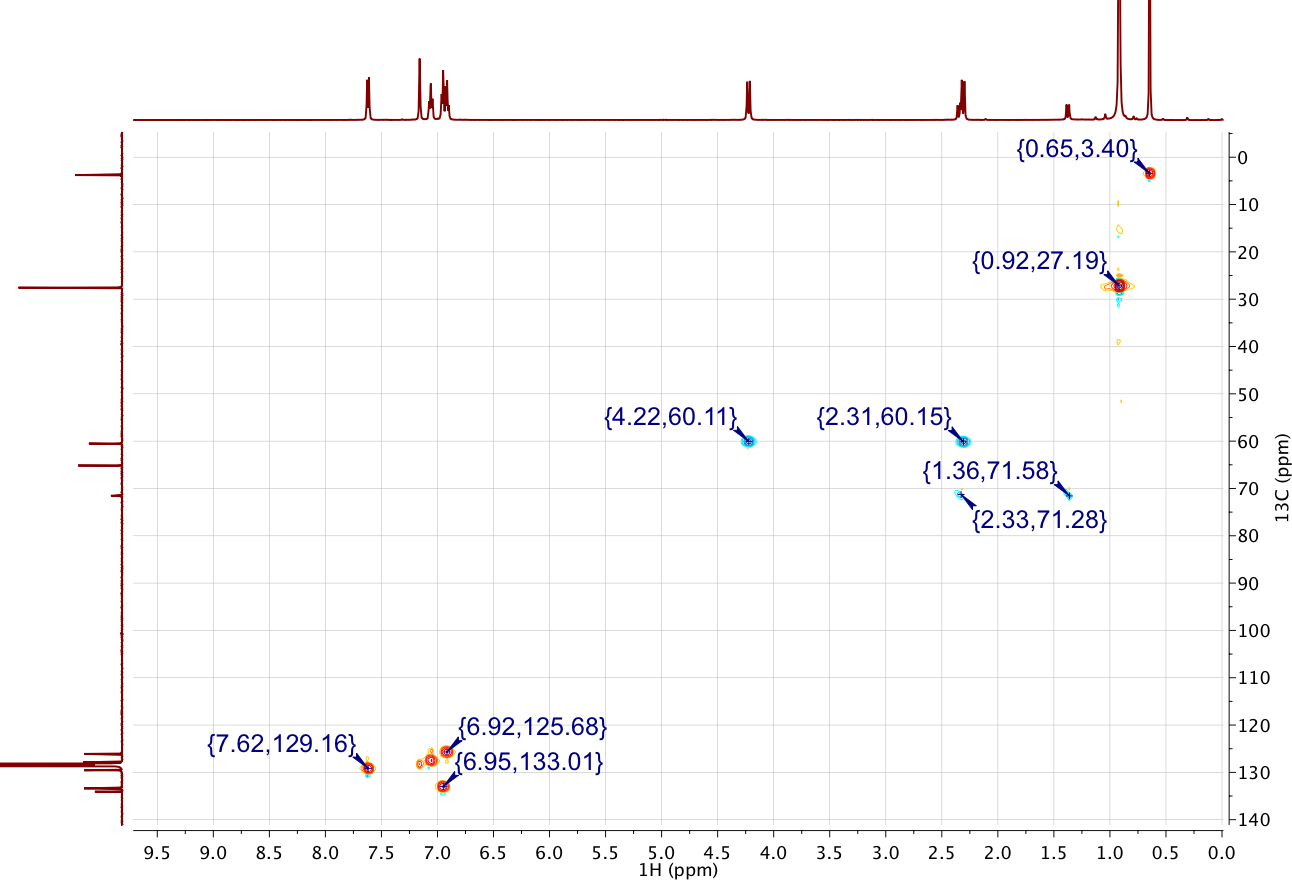


Figure S ^13^C–^1^H HSQC NMR (C_6_D_6_, 125.7 MHz, 300 K) spectrum of 2.


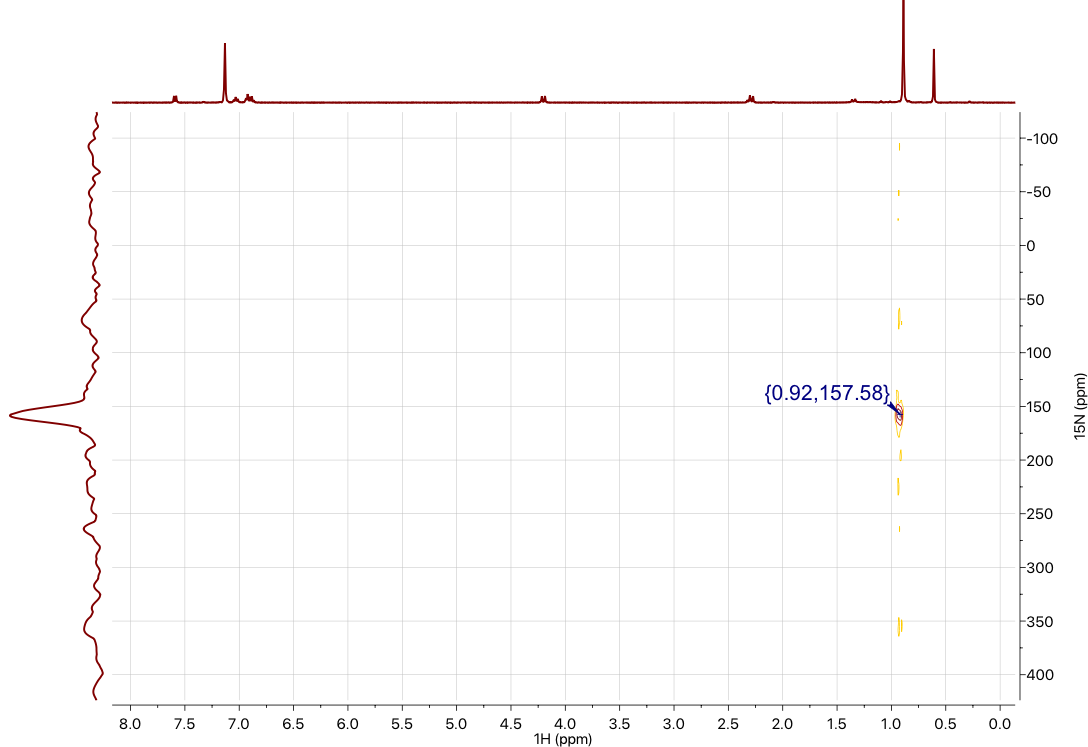


Figure S ^15^N–^1^H HMBC NMR (C_6_D_6_, 40.5 MHz, 300 K) spectrum of 2.


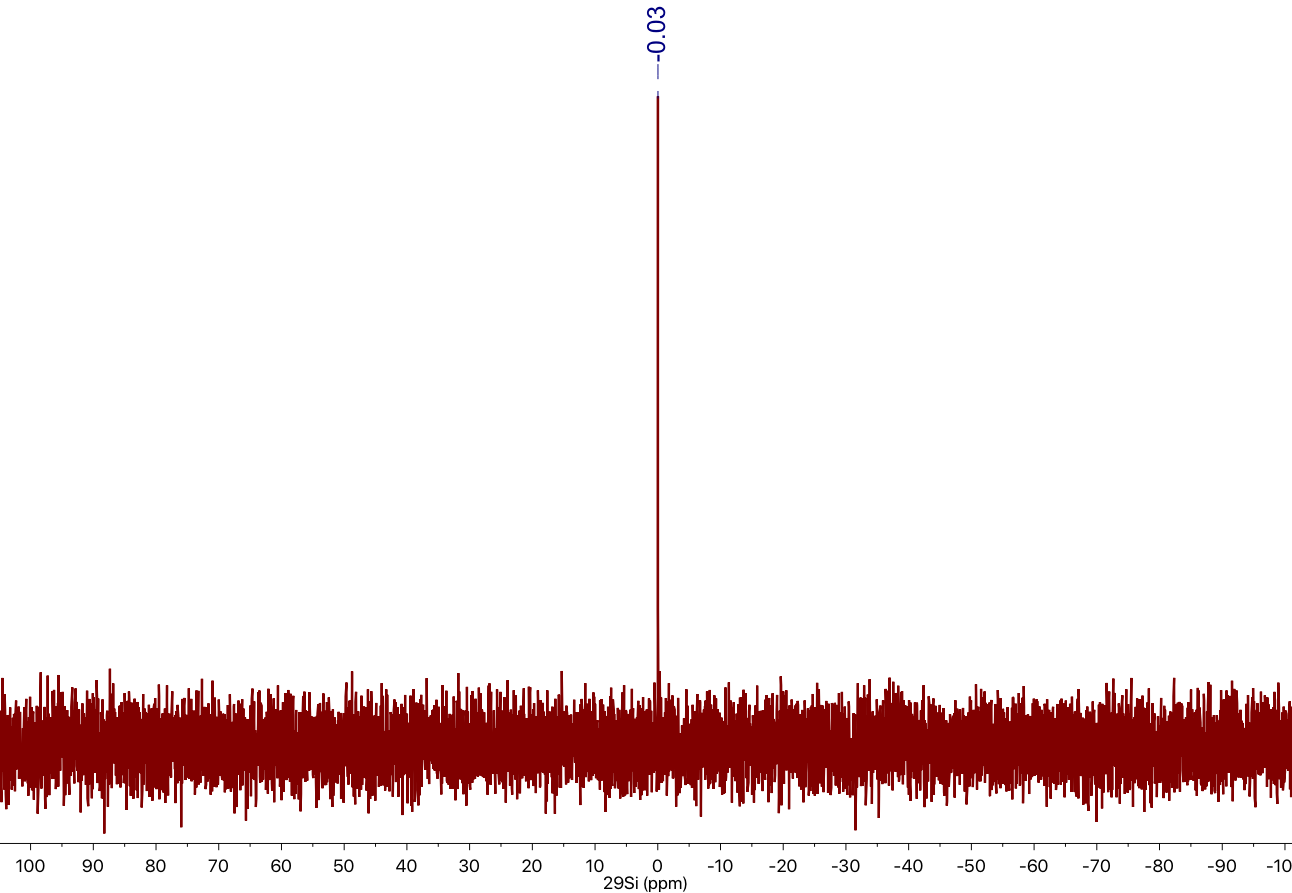


Figure S ^29^Si–^1^H INEPT NMR (C_6_D_6_, 79.5 MHz, 300 K) spectrum of 2.

| **[Ti(OCH_2_SiMe_3_)(TriNOx*)] (3).** [Ti(CH_2_SiMe_3_)(TriNOx)] (102 mg, 0.15 mmol) was dissolved in toluene (2 mL) and stirred at 90 °C for 30 minutes. The volatiles were evaporated under high-vacuum to give a pale yellow solid in quantitative yield (103 mg). Crystalline material was obtained by slow evaporation of a benzene/(SiMe_3_)_2_O solution. |  |
| --- | --- |

^1^H NMR (C_6_D_6_, 500 MHz, 300 K) δ 7.69 (d, ^3^*J*_HH_= 8.1 Hz, 1H, Ar*H*), 7.48 (d, ^3^*J*_HH_= 7.6 Hz, 1H, Ar*H*), 7.27 (t, ^3^*J*_HH_= 7.6 Hz, 1H, Ar*H*), 7.22 (d, ^3^*J*_HH_= 7.4 Hz, 1H, Ar*H*), 7.09–7.01 (m, 2H, Ar*H*), 7.01 (m, 5H), 6.86 (td, ^3^*J*_HH_= 7.4 Hz, ^4^*J*_HH_= 2.0 Hz, 1H, Ar*H*), 4.96 (ABq, ∆δ_AB_ = 0.05 Hz, ^2^*J*_HH_= 14.2 Hz, 2H, OC*H*_2_Si), 4.54 (at, ^2^*J*_HH_= 12.1 Hz, 2H, NC*H*_2_), 3.88 (d, ^2^*J*_HH_= 10.5 Hz, 1H, NC*H*_2_), 2.56 (d, ^2^*J*_HH_= 12.5 Hz, 1H, NC*H*_2_), 2.47 (d, ^2^*J*_HH_= 11.3 Hz, 1H, NC*H*_2_), 2.41 (d, ^2^*J*_HH_= 10.5 Hz, 1H, NC*H*_2_), 1.12 (s, 9H, C(C*H*_3_)_3_), 1.04 (s, 9H, C(C*H*_3_)_3_), 0.86 (s, 9H, C(C*H*_3_)_3_), 0.31 (s, 9H, Si(C*H*_3_)_3_).

^13^C{^1^H} NMR (C_6_D_6_, 125.7 MHz, 300 K) δ 152.2 (s, Ar), 150.3 (s, Ar), 149.1 (s, Ar), 136.4 (s, Ar), 135.8 (s, Ar), 134.5 (s, Ar), 132.0 (s, Ar), 131.6 (s, Ar), 131.5 (s, Ar), 129.3 (s, Ar), 128.9 (s, Ar), 128.1 (s, Ar), 127.8 (s, Ar), 127.4 (s, Ar), 127.0 (s, Ar), 126.7 (s, Ar), 124.1 (s, Ar), 123.5 (s, Ar), 71.4 (s, O*C*H_2_Si), 65.0 (s, *C*(CH_3_)_3_), 63.3 (s, *C*(CH_3_)_3_), 61.4 (s, *C*(CH_3_)_3_), 61.0 (s, N*C*H_2_), 60.5 (s, N*C*H_2_), 59.6 (s, N*C*H_2_), 31.7 (s, C(*C*H_3_)_3_), 27.8 (s, C(*C*H_3_)_3_), 25.9 (s, C(*C*H_3_)_3_), −2.4 (s, Si(*C*H_3_)_3_).

^15^N–^1^H HMBC NMR (C_6_D_6_, 40.5 MHz, 300 K) δ 269.9 (*N*(*^t^*Bu)), 211.7 (κ^1^-*N*O(*^t^*Bu)), 147.1 (κ^2^-*N*O(*^t^*Bu)).

^29^Si–^1^H INEPT NMR (C_6_D_6_, 79.5 MHz, 300 K) δ −2.57 (s, OCH_2_*Si*Me_3_).

Anal. Cald. for C_37_H_56_N_4_O_3_SiTi: C, 65.27; H, 8.29; N, 8.23. Found: C, 65.04; H, 7.95; N, 8.01.


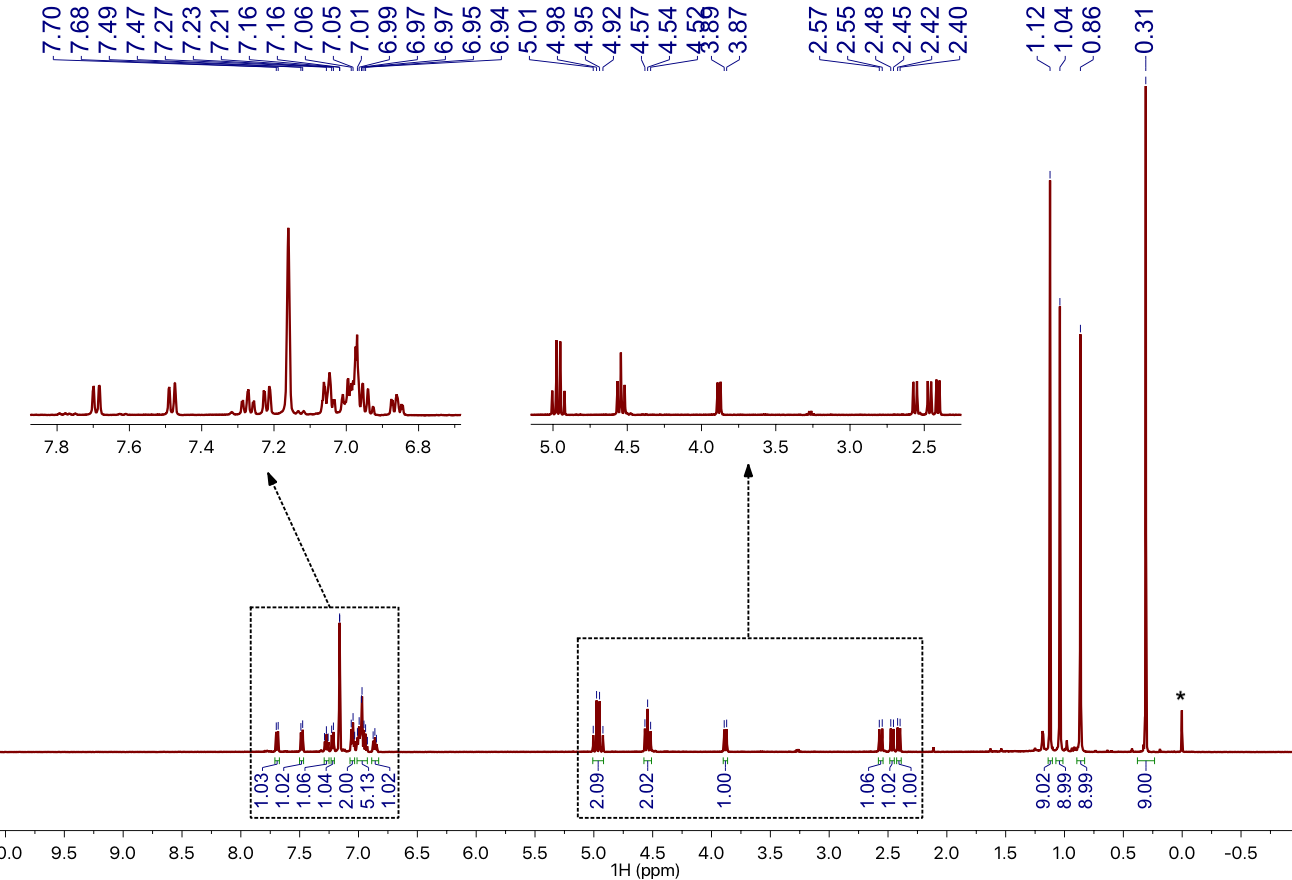


Figure S ^1^H NMR (C_6_D_6_, 500 MHz, 300 K) spectrum of 3. * denotes an impurity of SiMe_4_.


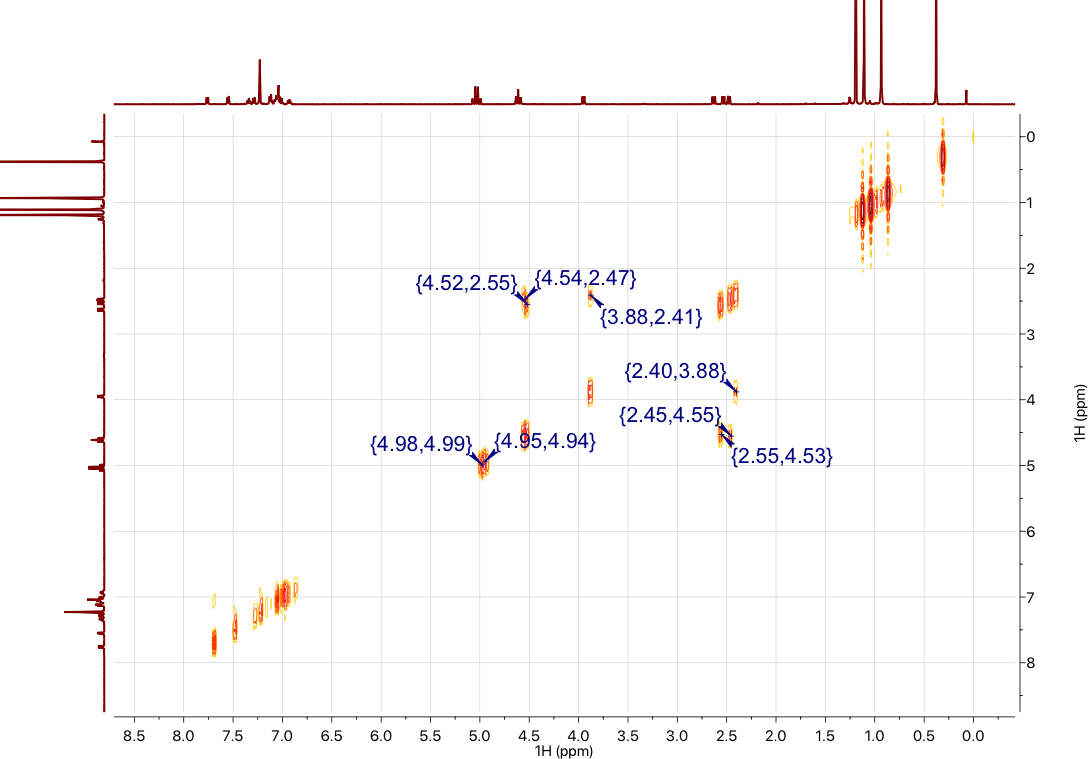


Figure S ^1^H–^1^H COSY NMR (C_6_D_6_, 500 MHz, 300 K) spectrum of 3.


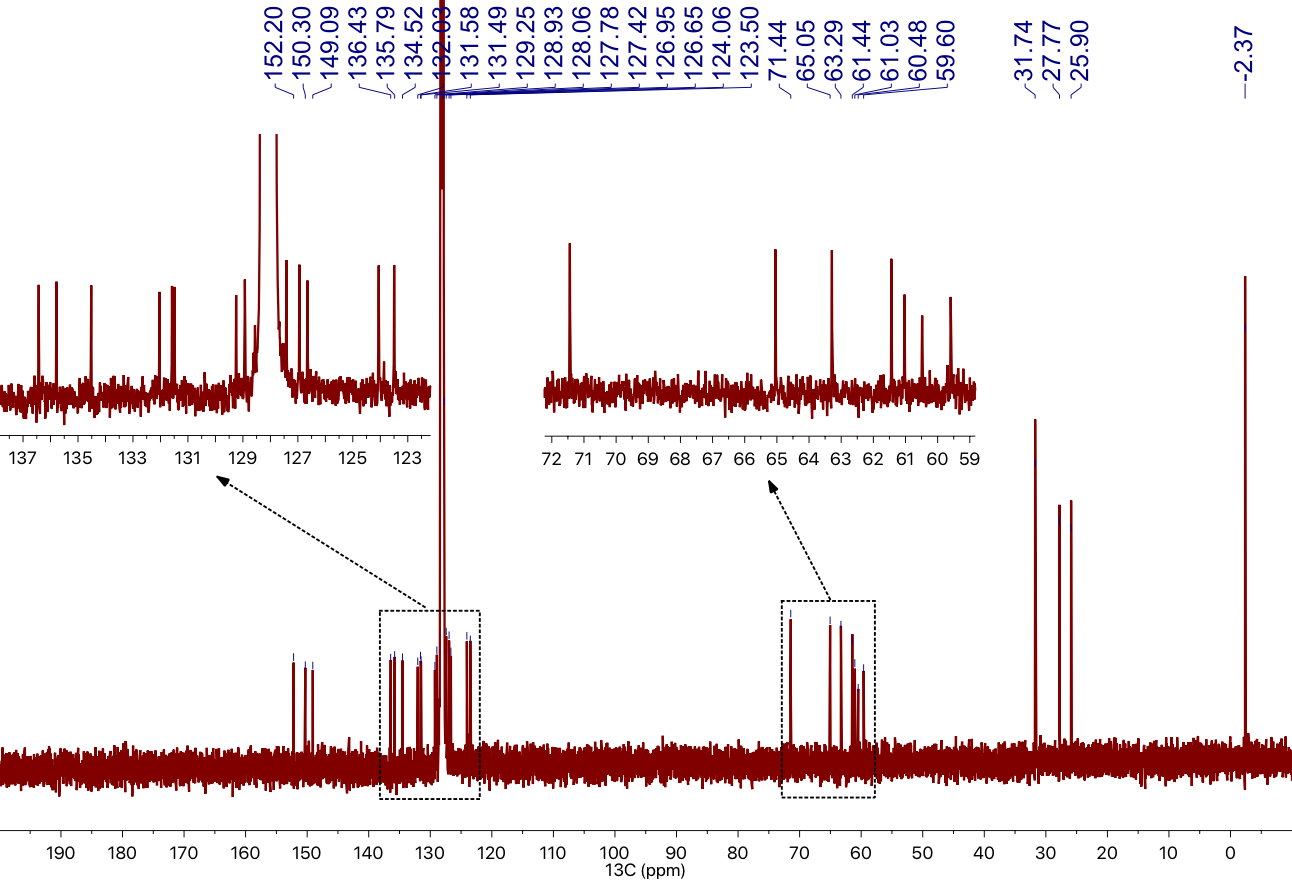


Figure S ^13^C{^1^H} NMR (C_6_D_6_, 125.7 MHz, 300 K) spectrum of 3.


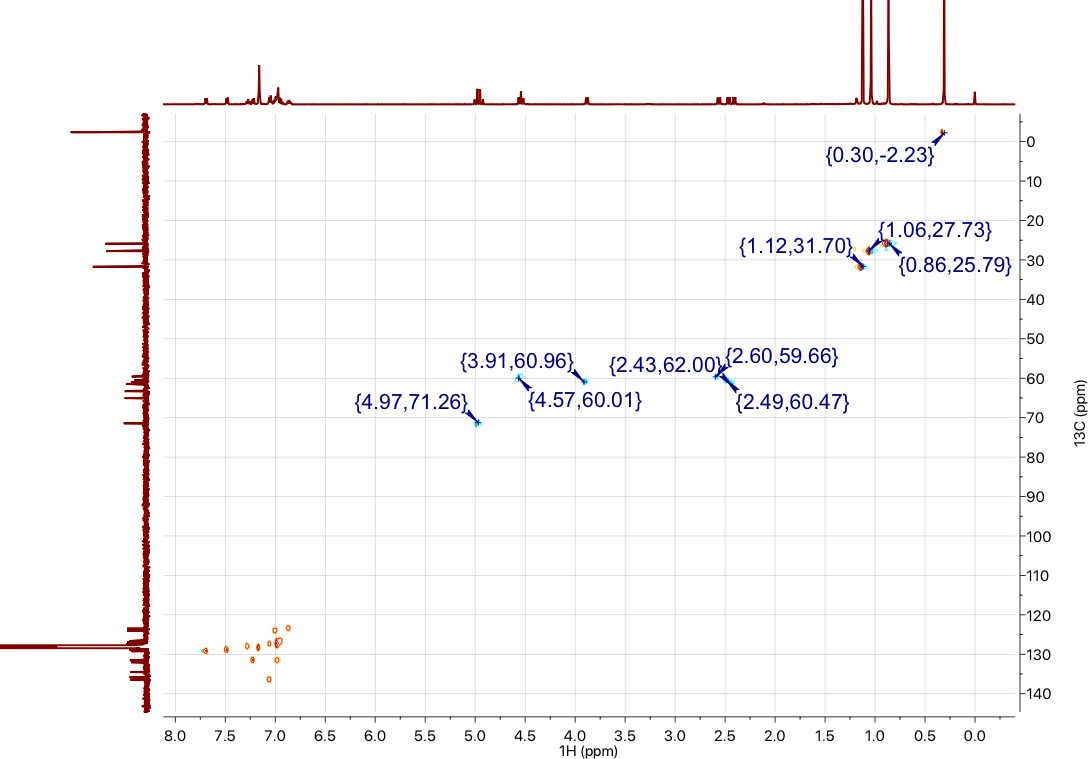


Figure S ^13^C–^1^H HSQC NMR (C_6_D_6_, 125.7 MHz, 300 K) spectrum of 3.


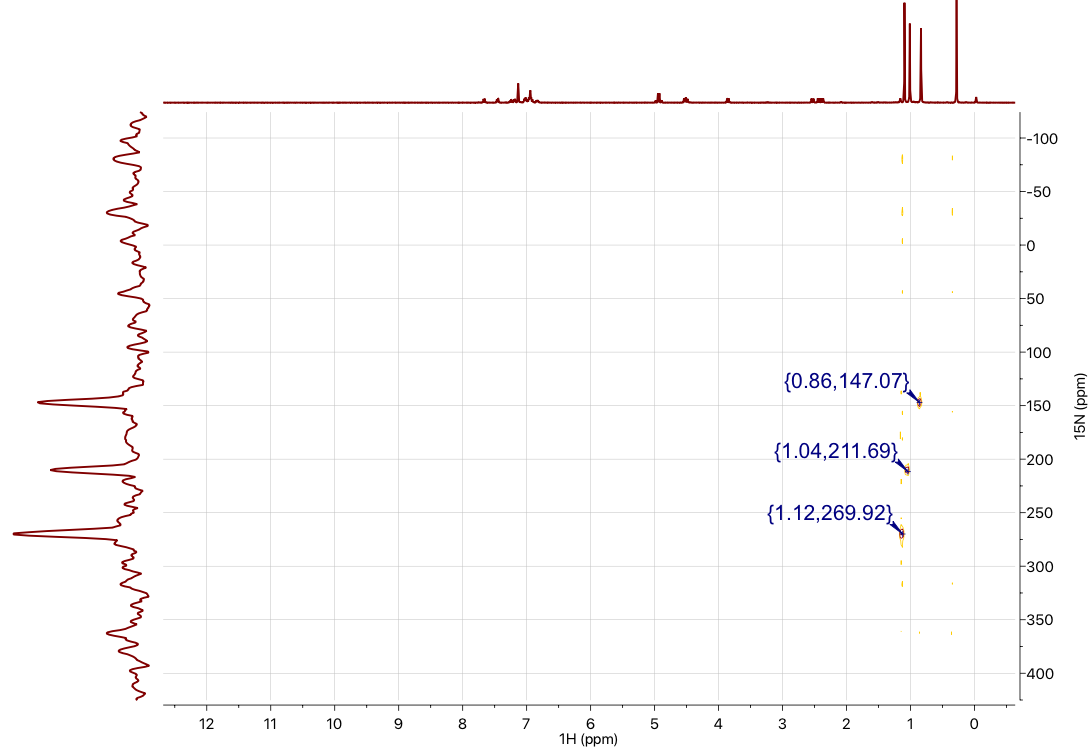


Figure S ^15^N–^1^H HMBC NMR (C_6_D_6_, 40.5 MHz, 300 K) spectrum of 3.


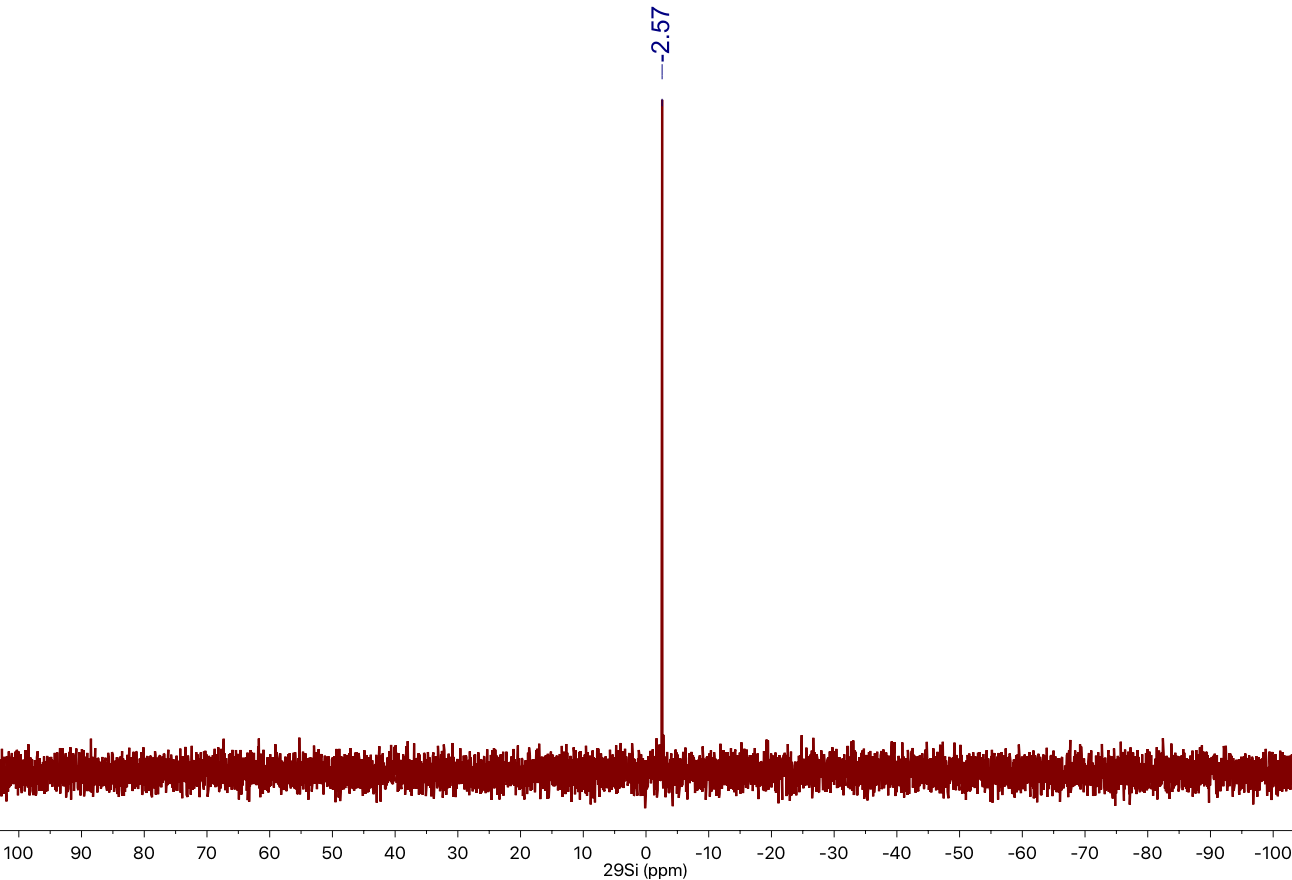


Figure S ^29^Si–^1^H INEPT NMR (C_6_D_6_, 79.5 MHz, 300 K) spectrum of 3.

| **[Ti(TriNOx*)]OTf (4_OTf_).** [Ti(OCH_2_SiMe_3_)(TriNOx*)] (65 mg, 95 µmol) was dissolved in CH_2_Cl_2_ (4 mL) and excess SiMe_3_OTf (~220 mg, 10 equiv) was added dropwise leading to a color change to red. The mixture was heated at 50 °C for 30 minutes giving a dark red solution. The volatiles were evaporated under reduced pressure and the solid was suspended in a mixture of *n*-pentane and toluene (1:1, 10 mL). The precipitate was filtered and washed with *n*-pentane (5 mL) and dried under reduced pressure to give a pink solid (57 mg, 78 µmol, 82%). |  |
| --- | --- |

^1^H NMR (CD_2_Cl_2_, 400 MHz, 300 K) δ 7.77 (d, ^3^*J*_HH_= 8.0 Hz, 1H, Ar*H*), 7.70–7.42 (m, 9H, Ar*H*), 7.28 (t, ^3^*J*_HH_= 7.4 Hz, 1H, Ar*H*), 7.05 (d, ^3^*J*_HH_= 8.2 Hz, 1H, Ar*H*), 4.92 (d, ^2^*J*_HH_= 13.0 Hz, 1H, NC*H*_2_), 4.07 (d, ^2^*J*_HH_= 13.2 Hz, 1H, NC*H*_2_), 4.01 (d, ^2^*J*_HH_= 12.9 Hz, 1H, NC*H*_2_), 3.82 (d, ^2^*J*_HH_= 13.0 Hz, 1H, NC*H*_2_), 3.78 (d, ^2^*J*_HH_= 13.2 Hz, 1H, NC*H*_2_), 3.74 (d, ^2^*J*_HH_= 12.9 Hz, 1H, NC*H*_2_), 1.13 (s, 9H, C(C*H*_3_)_3_), 0.94 (s, 9H, C(C*H*_3_)_3_), 0.81 (s, 9H, C(C*H*_3_)_3_).

^13^C{^1^H} NMR (C_6_D_6_, 125.7 MHz, 300 K) δ 142.3 (s, Ar), 141.3 (s, Ar), 141.1 (s, Ar), 133.4 (s, Ar), 133.3 (s, Ar), 133.1 (s, Ar), 132.7 (s, Ar), 131.7 (s, Ar), 131.6 (s, Ar), 130.9 (s, Ar), 130.8 (s, Ar), 130.66 (s, Ar), 130.63 (s, Ar), 130.62 (s, Ar), 130.3 (s, Ar), 130.0 (s, Ar), 128.8 (s, Ar), 127.4 (s, Ar), 121.3 (q, ^1^*J*_CF_= 322 Hz, SO_3_*C*F_3_) 70.0 (s, *C*(CH_3_)_3_), 69.3 (s, *C*(CH_3_)_3_), 66.4 (s, *C*(CH_3_)_3_), 61.7 (s, N*C*H_2_), 60.2 (s, N*C*H_2_), 58.8 (s, N*C*H_2_), 33.6 (s, C(*C*H_3_)_3_), 27.2 (s, C(*C*H_3_)_3_), 27.1 (s, C(*C*H_3_)_3_).

^15^N–^1^H HMBC NMR (CD_2_Cl_2_, 40.5 MHz, 300 K) δ 317.4 (*N*(*^t^*Bu)), 162.9 (*N*O(*^t^*Bu)), 147.3 (*N*O(*^t^*Bu)).

^19^F{^1^H} NMR (CD_2_Cl_2_, 376.3 MHz, 300 K) δ −78.8 (SO_3_C*F*_3_).

Anal. Cald. for C_34_H_45_F_3_N_4_O_5_STi: C, 56.20; H, 6.24; N, 7.84. Found: C, 56.56; H, 6.35; N, 7.46.


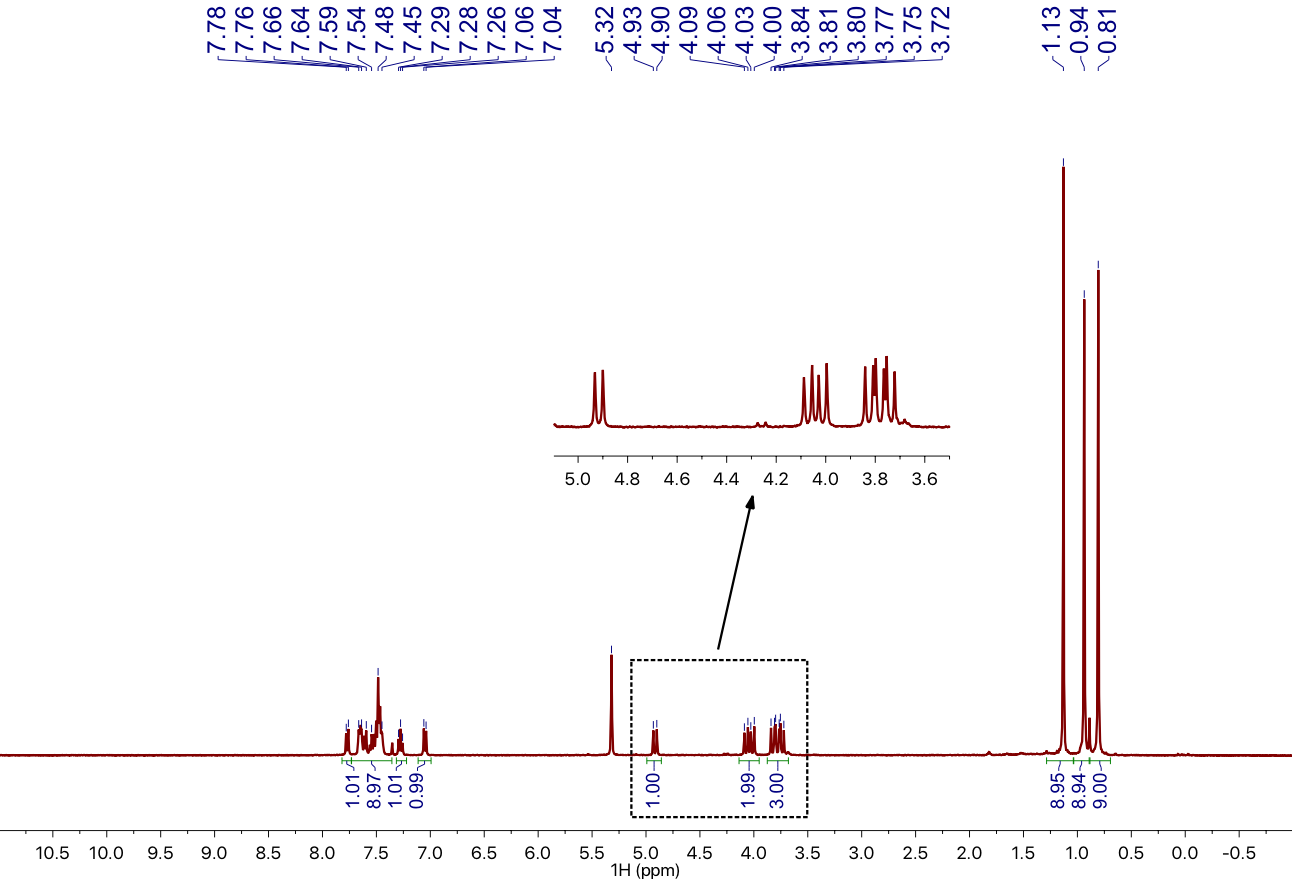


Figure S ^1^H NMR (CD_2_Cl_2_, 400 MHz, 300 K) spectrum of 4_OTf_.


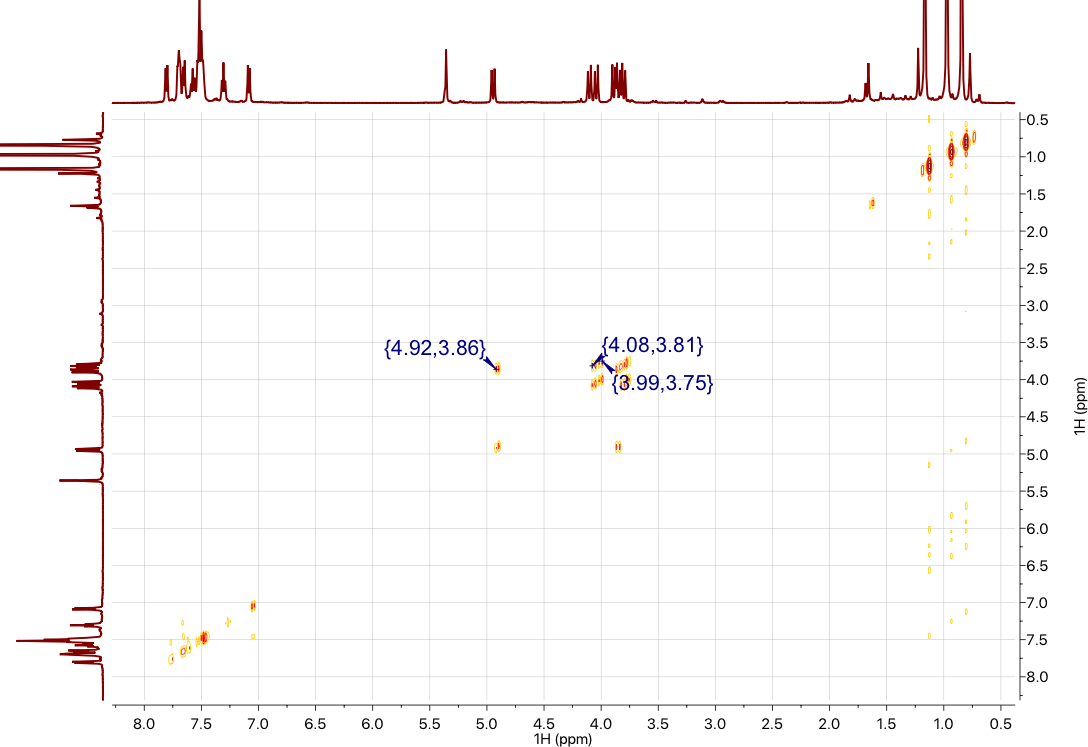


Figure S^1^H–^1^H COSY NMR (CD_2_Cl_2_, 500 MHz, 300 K) spectrum of 4_OTf_.


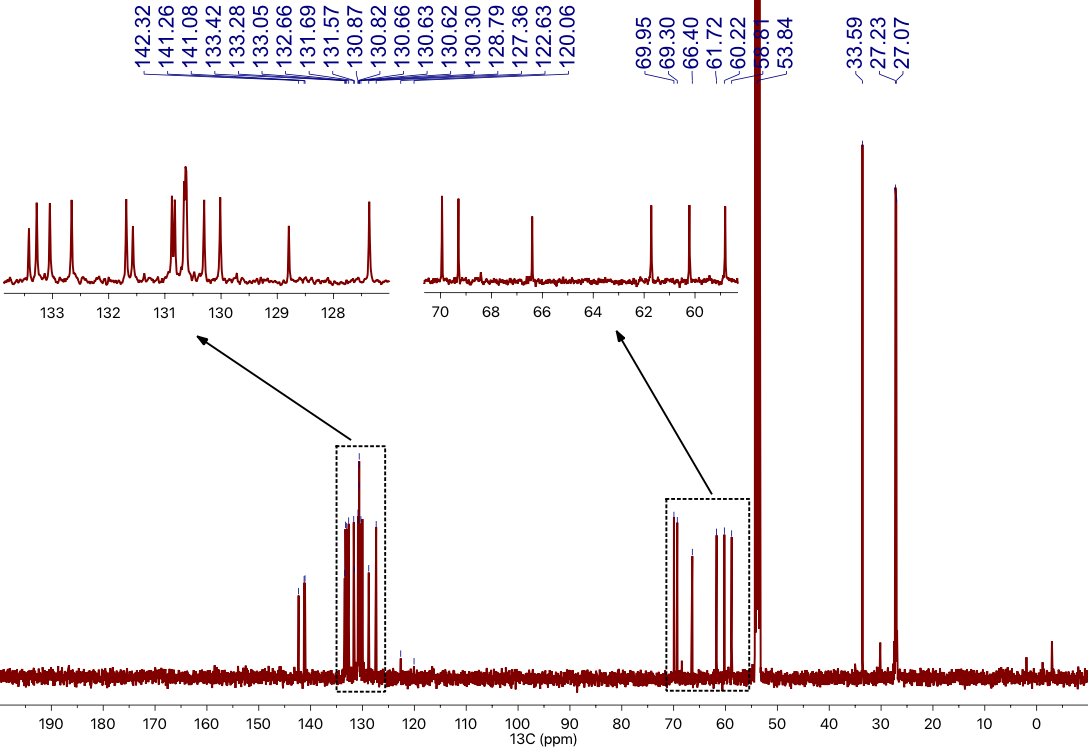


Figure S ^13^C{^1^H} NMR (CD_2_Cl_2_, 125.7 MHz, 300 K) spectrum of 4_OTf_.


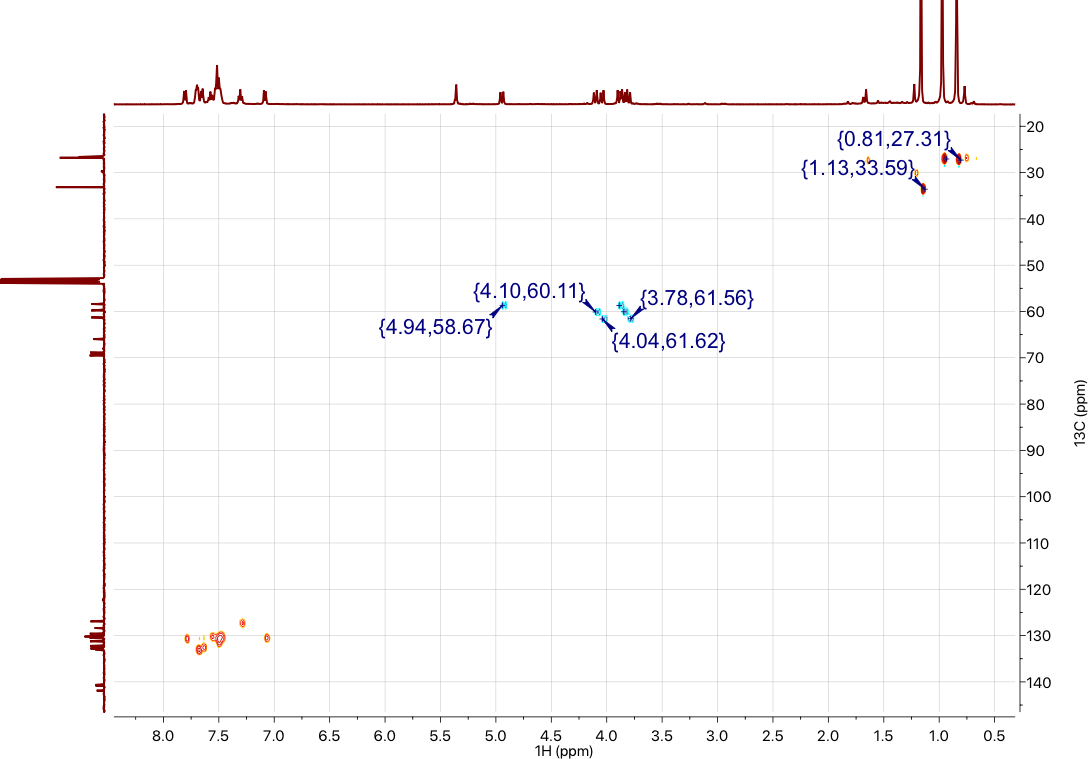


Figure S^13^C–^1^H HSQC NMR (CD_2_Cl_2_, 125.7 MHz, 300 K) spectrum of 4_OTf_.


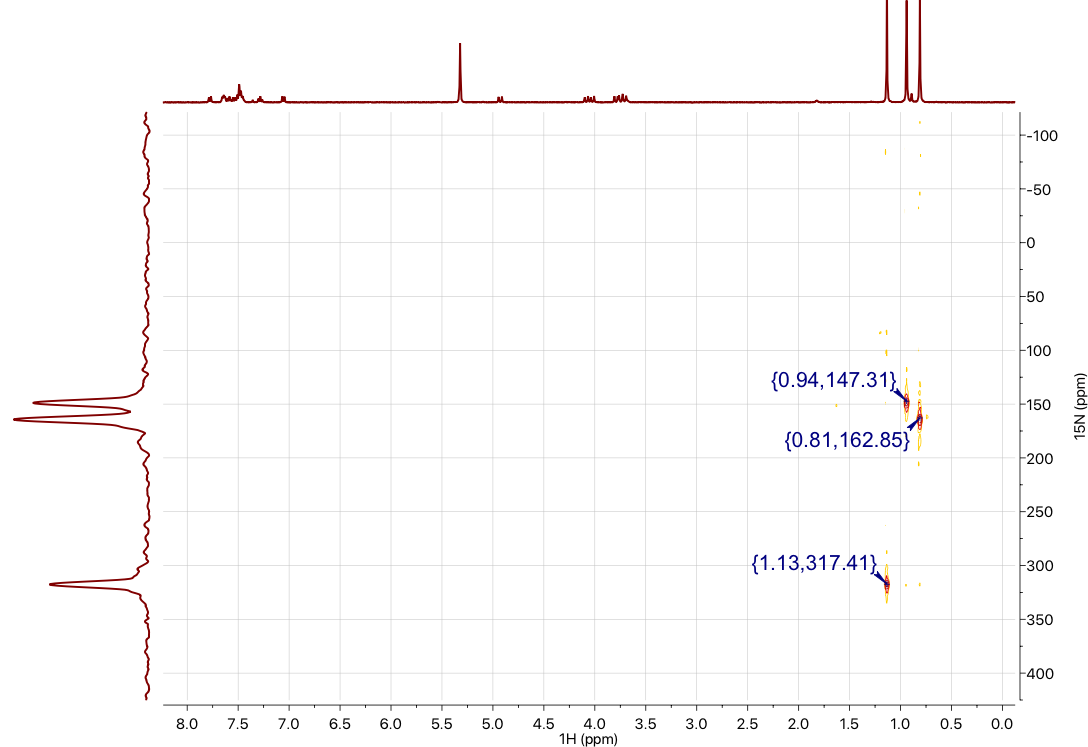


Figure S^15^N–^1^H HMBC NMR (CD_2_Cl_2_, 40.5 MHz, 300 K) spectrum of 4_OTf_.


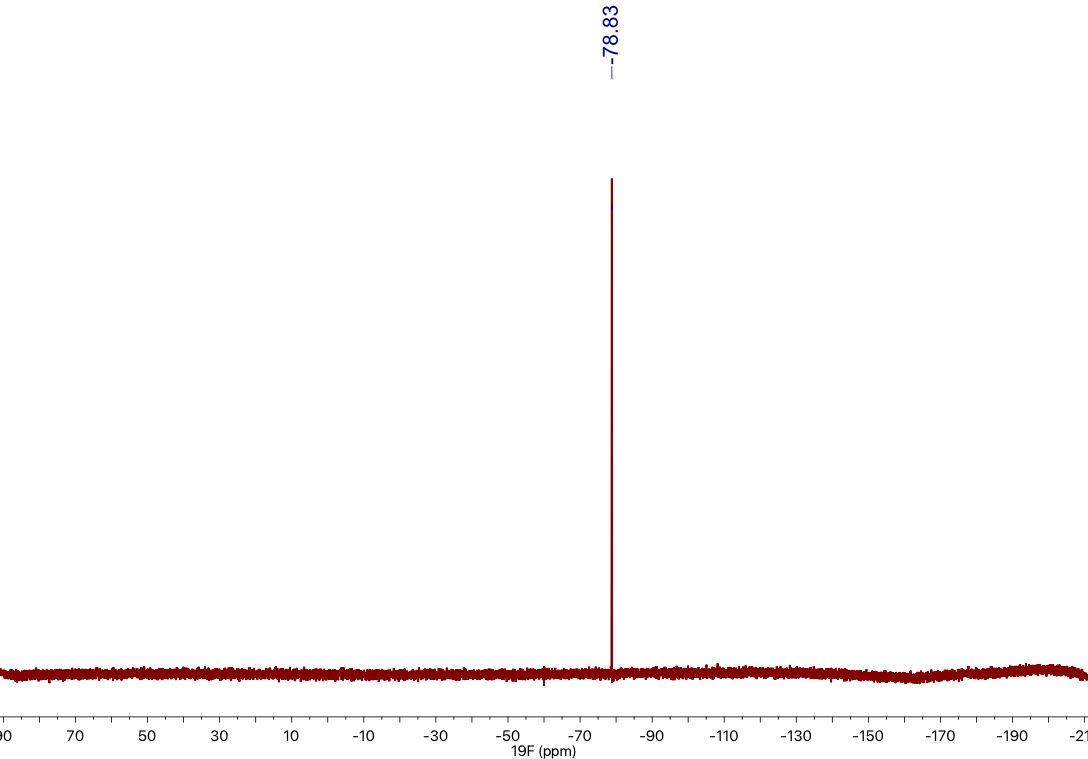


Figure S^19^F{^1^H} NMR (CD_2_Cl_2_, 376.3 MHz, 300 K) spectrum of 4_OTf_.

| **[Ti(TriNOx)]OTf (1_OTf_).** Method A: [Ti(TriNOx)]Cl (20 mg, 32 µmol) and AgOTf (8.2 mg, 32 µmol) were vigorously stirred in CH_2_Cl_2_ (10 mL) in the dark. After 10 minutes, the precipitate of AgCl was filtered and washed with CH_2_Cl_2_ (2 mL). The filtrate was evaporated under reduced pressure to afford a pale yellow powder of the desired compound (20.4 mg, 27 µmol, 86%). Single crystals suitable for X-ray crystallography were obtained by gas diffusion of Et_2_O into a concentrated CH_2_Cl_2_ solution. |  |
| --- | --- |

Method B: Solid N-methylmorpholine N-oxide (16.4 mg, 0.14 mmol, 3 equiv) was added to a precooled (−20 °C) solution of [Ti(TriNOx*)]OTf (34 mg, 47 µmol, 1 equiv) in CH_2_Cl_2_ (2 mL). The mixture was stirred at room temperature for 5 minutes and at 50 °C for 1 hour. During the course of the reaction to mixture color changed from pink to yellow and some precipitate started to form. The volatiles were evaporated and the solid was suspended in THF (5 mL). The solid was filtered and washed successively with Et_2_O (5 mL) and *n*-pentane (5 mL) to afford a pale yellow powder (26.8 mg, 36 µmol, 77%).

^1^H NMR (CD_2_Cl_2_, 500 MHz, 300 K) δ 7.65 (d, ^3^*J*_HH_= 7.5 Hz, 3H, Ar*H*), 7.55–7.55 (m, 9H, Ar*H*), 4.26 (d, ^2^*J*_HH_= 12.7 Hz, 3H, NC*H*_2_), 3.70 (d, ^2^*J*_HH_= 12.7 Hz, 3H, NC*H*_2_), 0.89 (s, 9H, C(C*H*_3_)_3_).

^13^C{^1^H} NMR (CD_2_Cl_2_, 125.7 MHz, 315 K) δ 143.8 (s, Ar), 133.5 (s, Ar), 132.2 (s, Ar), 130.54 (s, Ar), 130.49 (s, Ar), 130.2 (s, Ar), 68.2 (s, *C*(CH_3_)_3_), 61.2 (s, N*C*H_2_), 27.2 (s, C(*C*H_3_)_3_). Due to the low solubility of the compound, the quadruplet associated with the triflate counter-anion was not observed.

^19^F{^1^H} NMR (CD_2_Cl_2_, 376.3 MHz, 300 K) δ −78.8 (s, C*F*_3_).

Anal. Cald. for C_34_H_45_F_3_N_4_O_6_STi: C, 54.99; H, 6.11; N, 7.54. Found: C, 55.20; H, 6.00; N, 7.23.


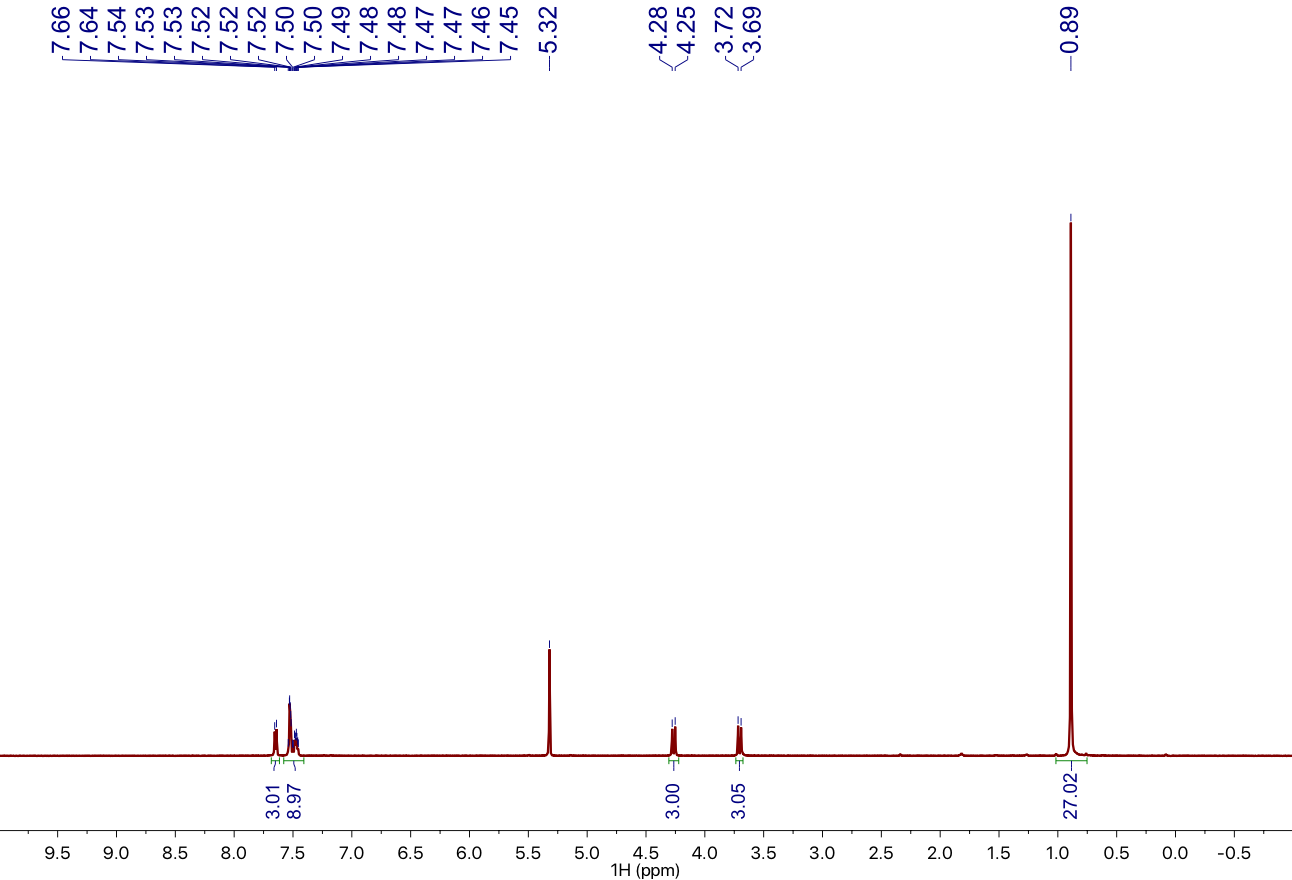


Figure S^1^H NMR (CD_2_Cl_2_, 500 MHz, 300 K) spectrum of 1_OTf_ (Method A).


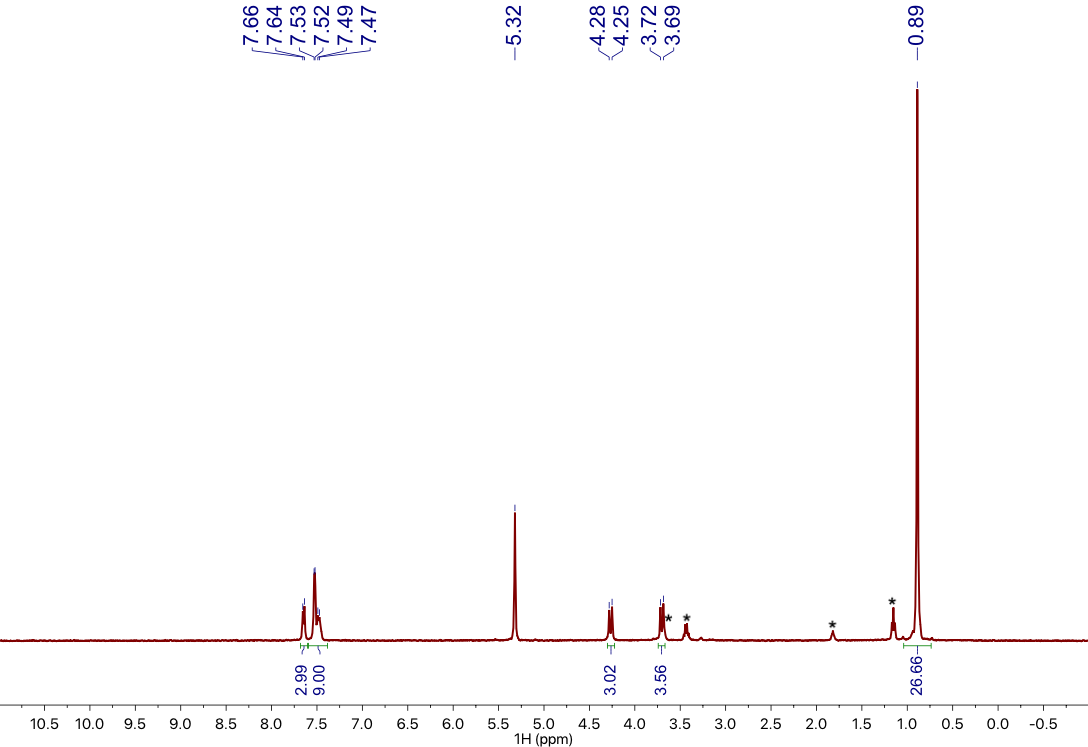


Figure S^1^H NMR (CD_2_Cl_2_, 400 MHz, 300 K) spectrum of 1_OTf_ (Method B). * denotes impurities of THF and Et_2_O.


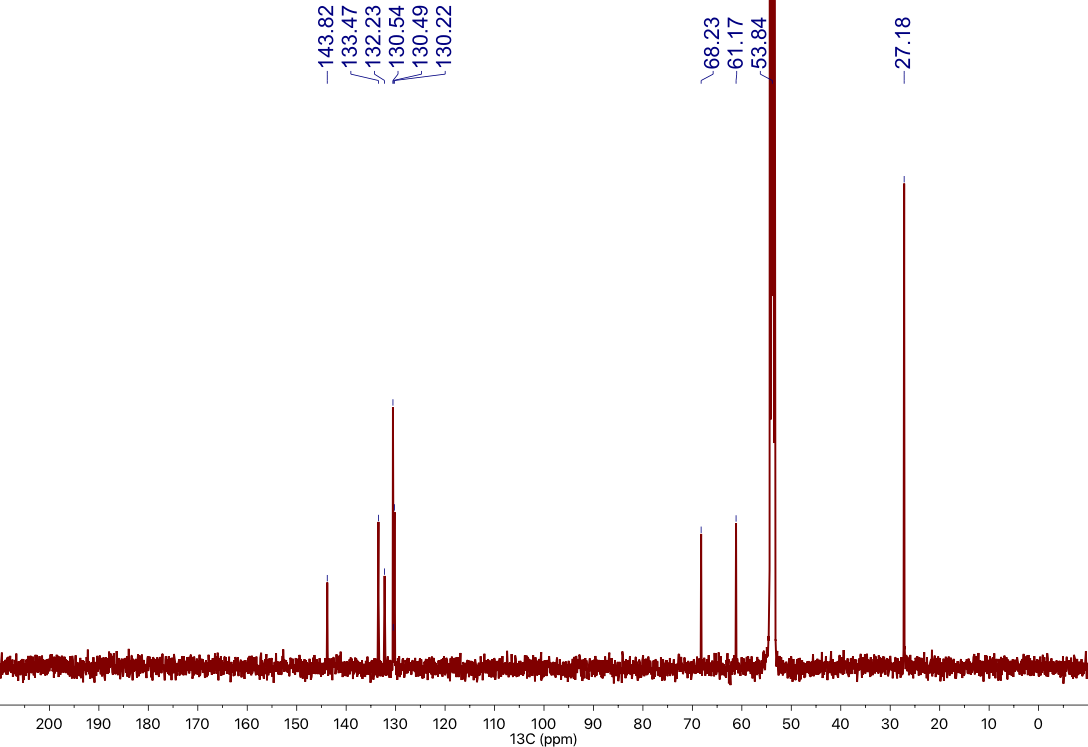


Figure S^13^C{^1^H} NMR (CD_2_Cl_2_, 125.7 MHz, 315 K) spectrum of 1_OTf_.


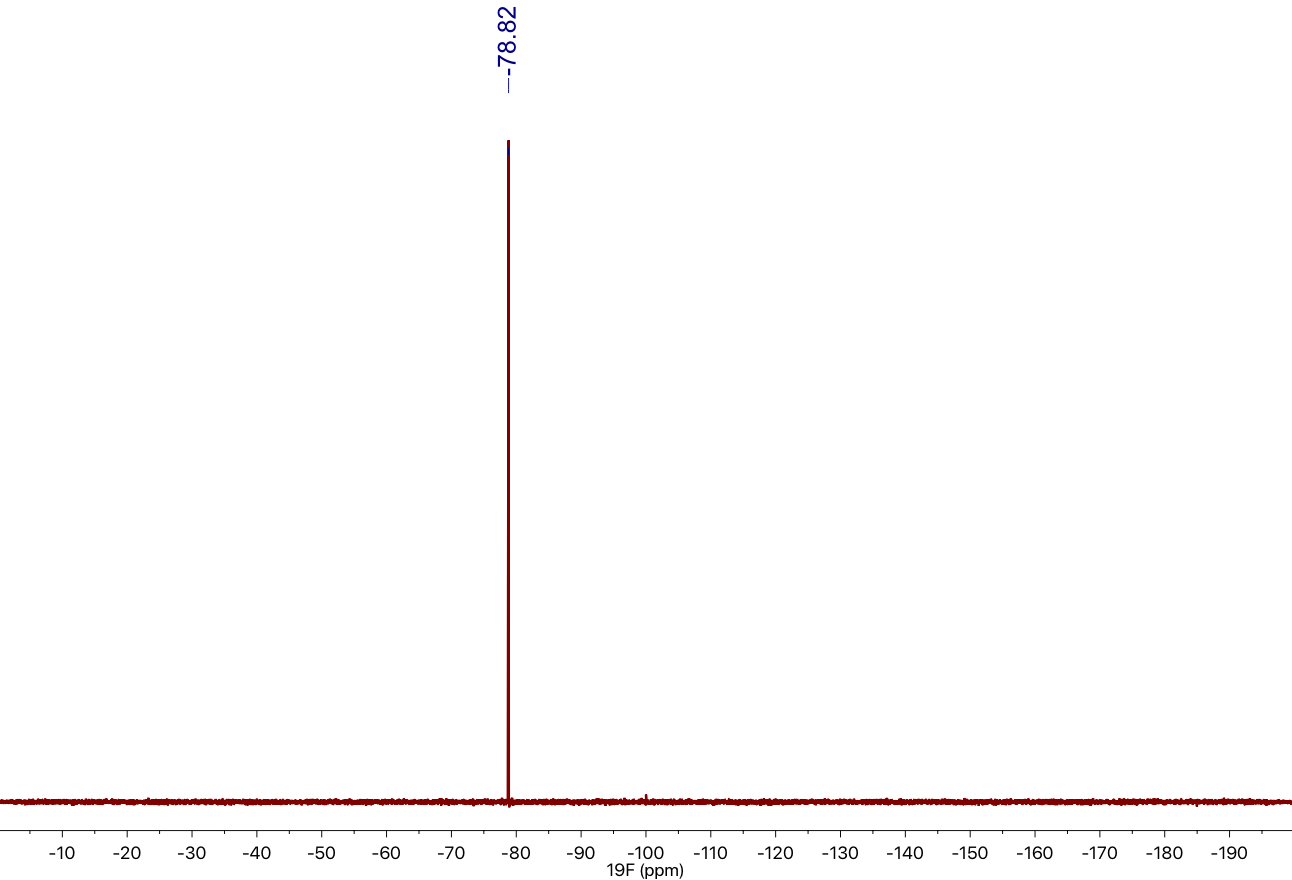


Figure S^19^F{^1^H} NMR (CD_2_Cl_2_, 376.3 MHz, 300 K) spectrum of 1_OTf_.

**UV-Visible Spectroscopy.**

UV-Visible spectra were obtained using a Perkin Elmer 905 UV-Vis-NIR spectrophotometer. Solutions of known concentration (0.073 mM in THF for **2**, 0.23 mM in THF for **3**, 0.10 mM for **1_OTf_** in DCM, 0.17 mM in THF for **4_OTf_**) were prepared in N2 atmosphere and transferred to an airtight quartz cell with a pathlength of 1 cm. Blanks were collected prior to each run.


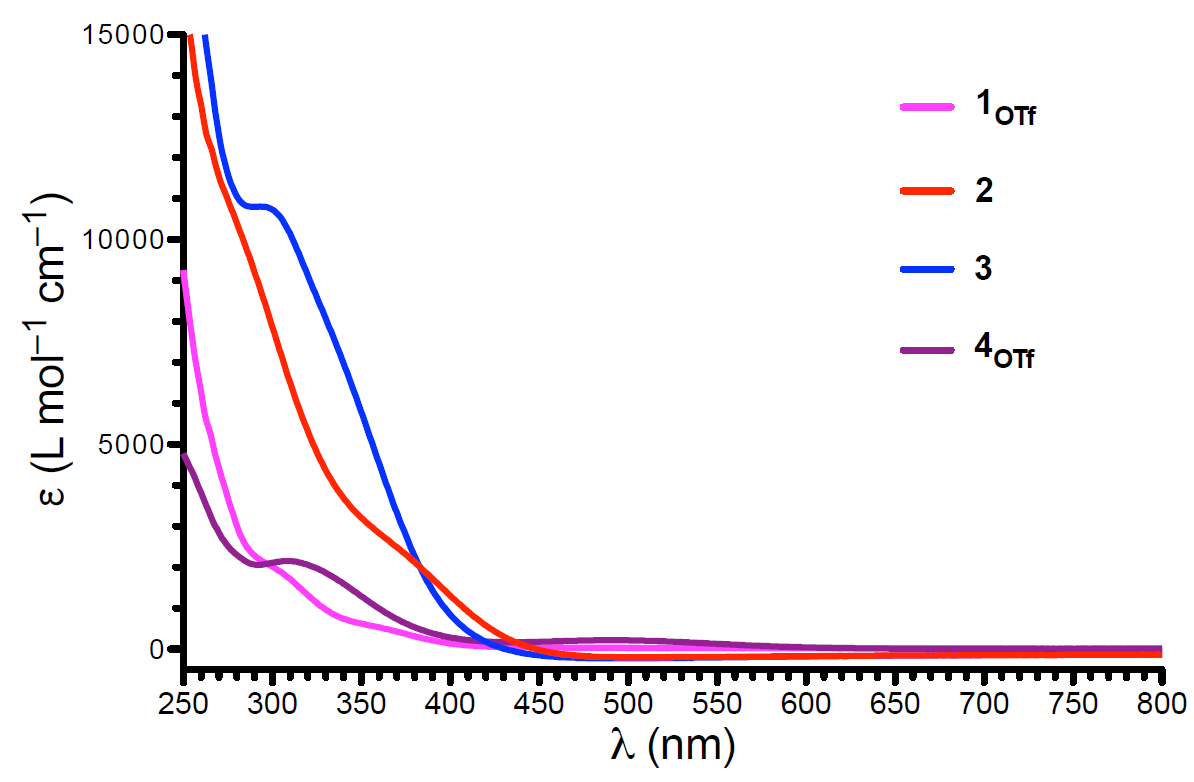


**Figure S** UV-Visible spectra of **1_OTf_** (pink) in DCM and **2** (blue), **3** (red) and **4_OTf_** (purple) in THF, all recorded at 300 K.

**Kinetic experiments.**

^1^H NMR spectra were collected on a Bruker UNI 400 Fourier Transform NMR spectrometer for the kinetics study. For each trial, NMR samples of know concentration of both **2** and ferrocene (as an internal standard) were prepared at room temperature and immediately inserted into the spectrometer; pre-heated to the experimental temperature (298, 313, 328, 343 K). In each case, the first spectrum was obtained exactly 5 minutes after sample insertion. Subsequent spectra were collected at varying intervals depending on experimental temperature, detailed in Figures S25-29. The resonances at δ = 4.23, 2.33 and .64 ppm (corresponding to the methylene and SiMe_3_ protons of **2**) were used to monitor **2** while the resonances at δ = 4.96, 4.53 and .31 ppm (corresponding to methylene and SiMe_3_ protons of **3**) were used to monitor the formation of **3**. With the integration of the resonance at δ = 4.00 (corresponding to the ferrocene internal standard) standardized to 10.00, the changing integrations of chosen resonances of **2** and **3** were converted into concentration. Plots of concentration versus time and ln(concentration) versus time were used to generate kinetics traces (Figure 2 in main text).


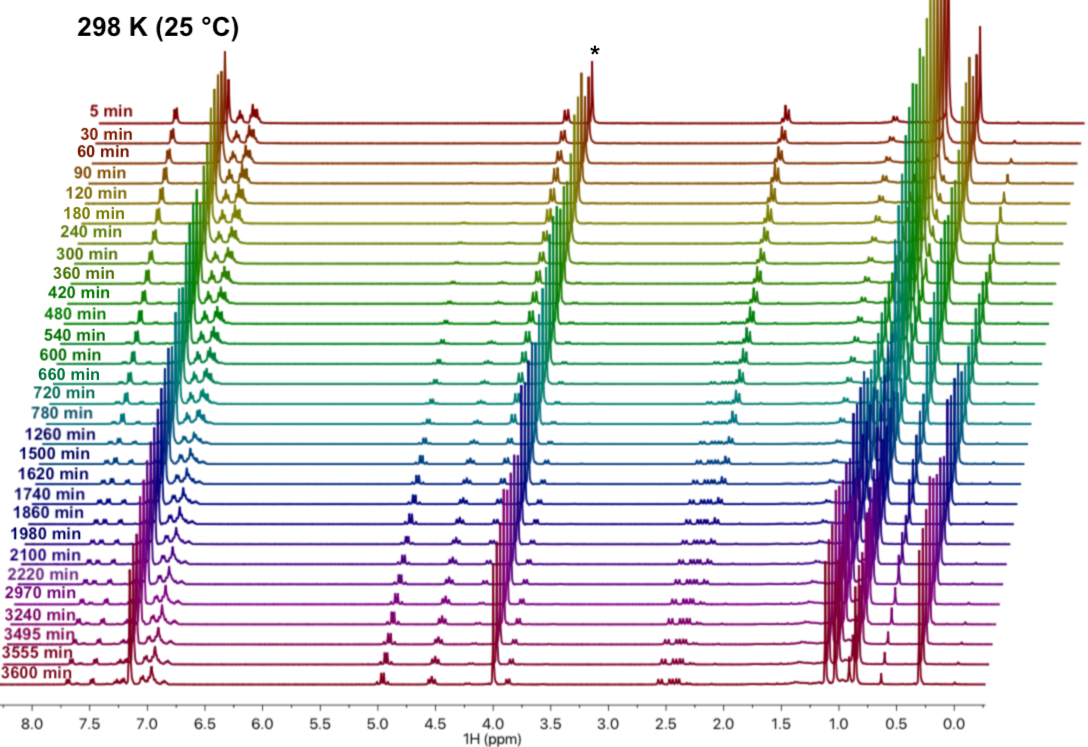


Figure S Kinetic ^1^H NMR spectra tracking the transformation of 2 to 3 at 298 K over a period of 60 hours with a ferrocene internal standard (*).


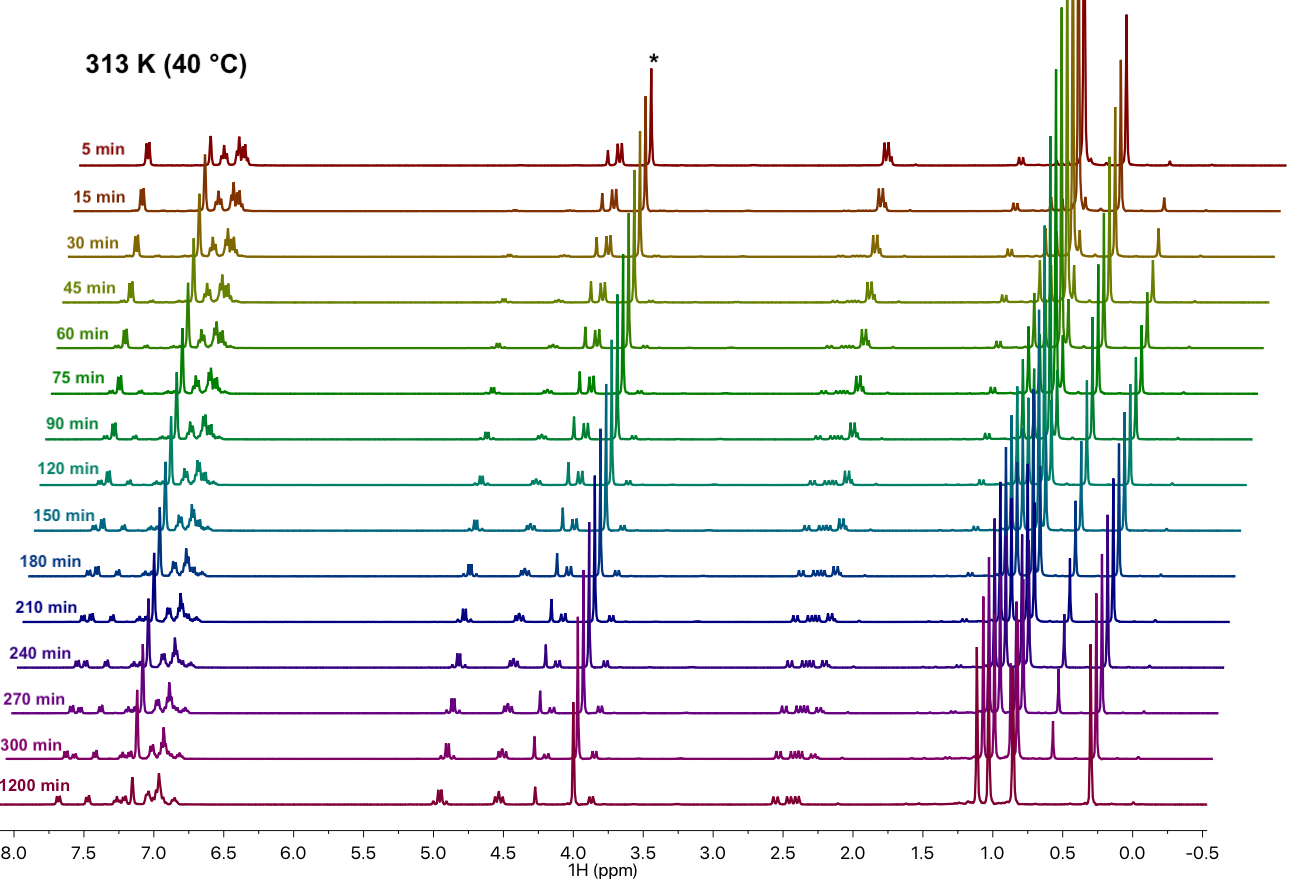


Figure S Kinetic ^1^H NMR spectra tracking the transformation of of 2 to 3 at 313 K with a ferrocene internal standard (*). Spectra were collected over a period of 5 hours with an additional point at 20 hours.


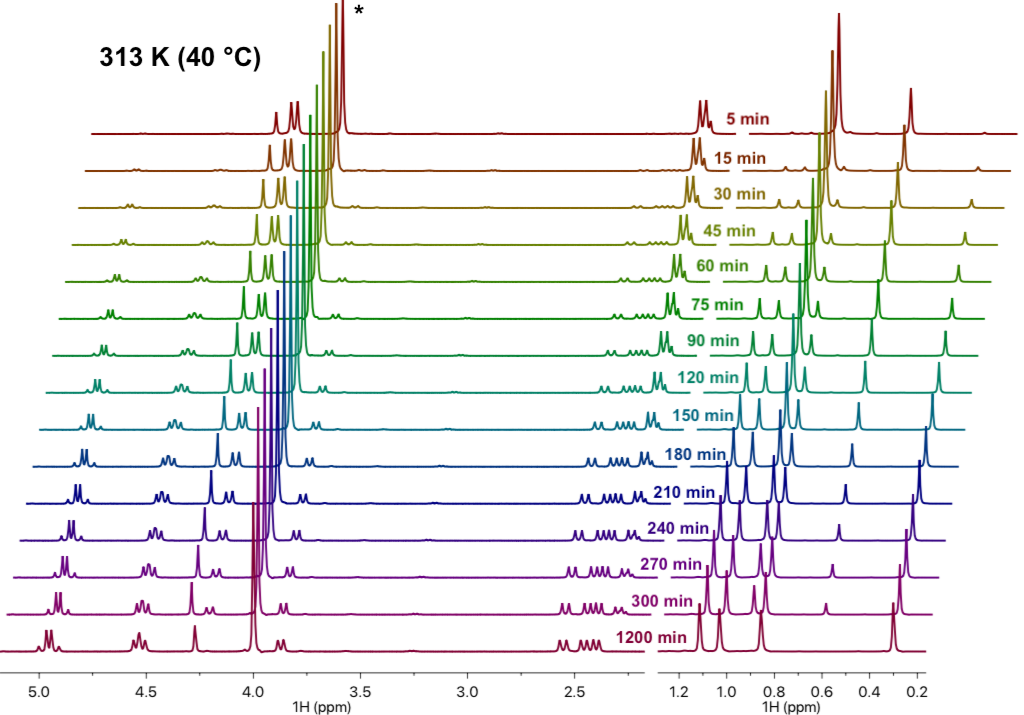


Figure S Close-up on the benzylic and *tert*-butyl groups ^1^H NMR regions tracking the transformation of 2 to 3 at 313 K with a ferrocene internal standard (*).


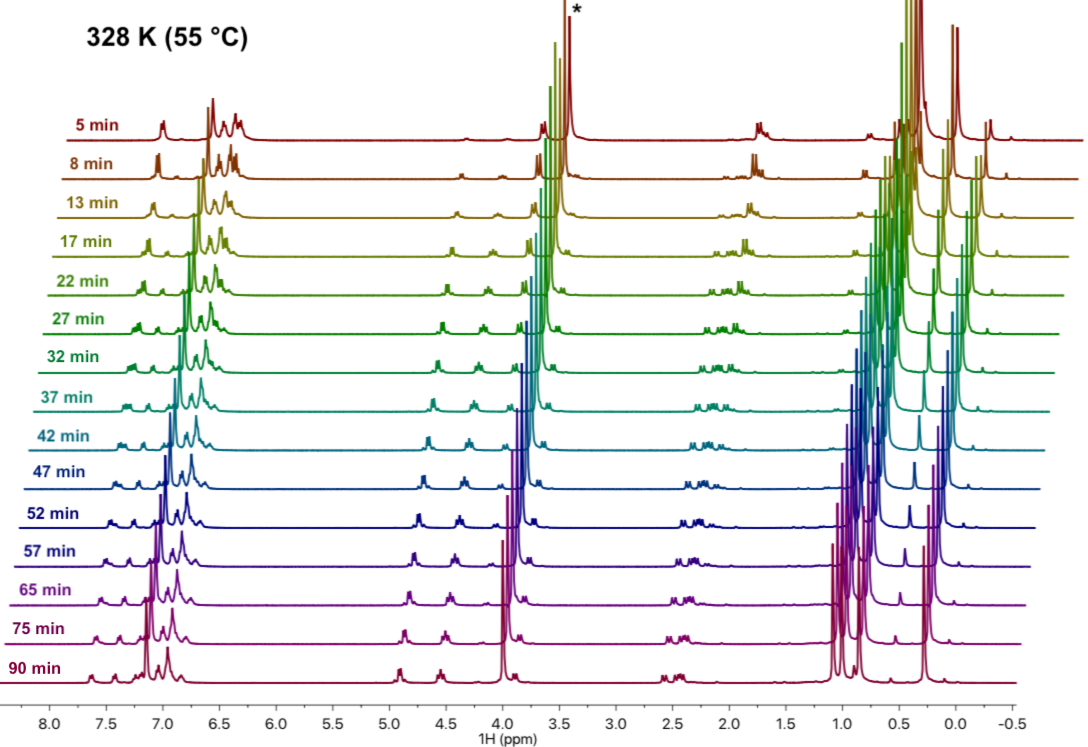


Figure S Kinetic ^1^H NMR spectra tracking the transformation of of 2 to 3 at 328 K over a period of 90 minutes with a ferrocene internal standard (*).


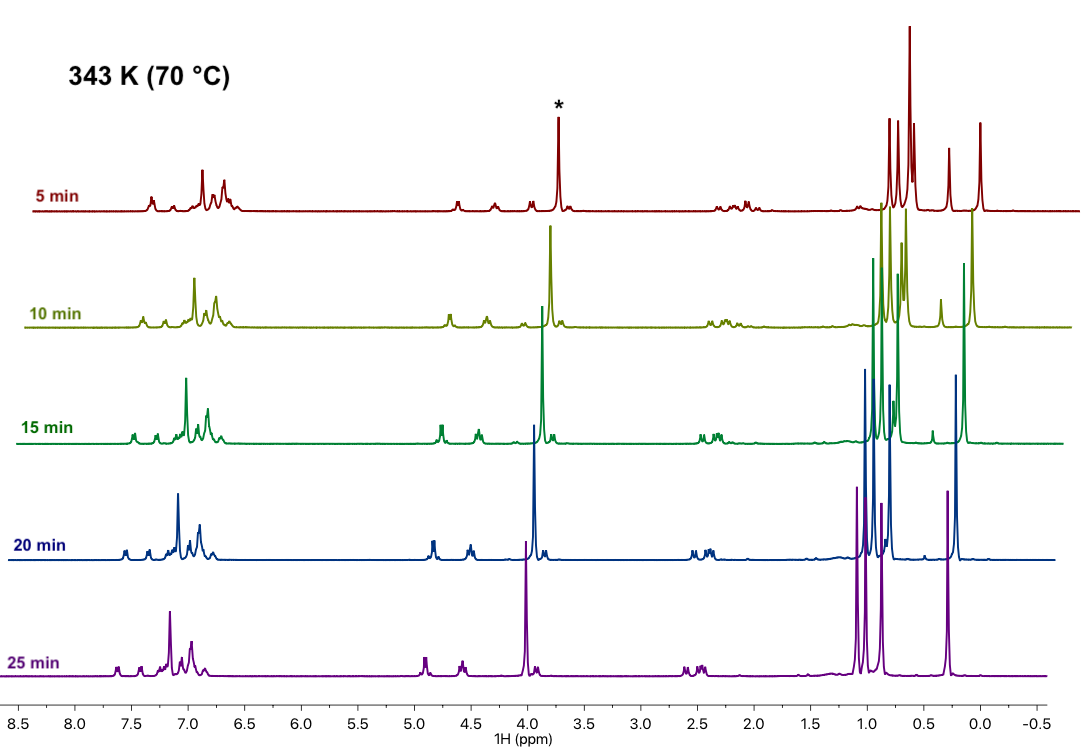


Figure S Kinetic ^1^H NMR spectra for the transformation of 2 to 3 at 343 K over a period of 25 minutes with a ferrocene internal standard (*).

**NMR-Scale Reactivity Studies.**

**• Conversion of [Ti(OCH_2_SiMe_3_)(TriNOx*)] (3) to [TiCl(TriNOx*)] (4_Cl_): 3** (10.2 mg, 15 µmol, 1 equiv) was dissolved in C_6_D_6_ (0.5 mL) and transferred to a J-Young NMR tube. SiMe_3_Cl (~30 µL, ~15 equiv) was added and the tube sealed and immerged in an oil bath preheated at 85 °C and periodically analyzed as depicted below.


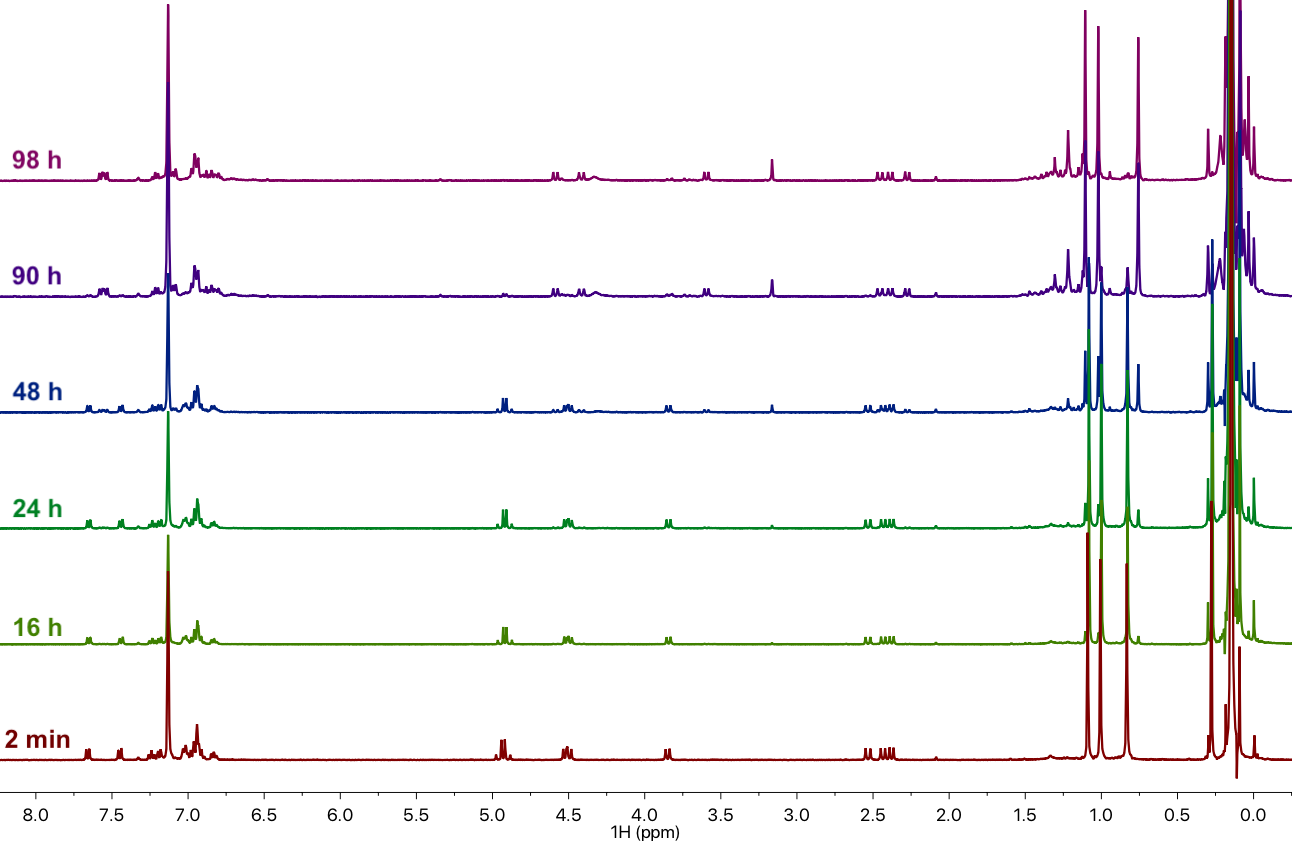


Figure S Reaction of [Ti(OCH_2_SiMe_3_)(TriNOx*)] (3) and SiMe_3_Cl in C_6_D_6_ at 85 °C to form [TiCl(TriNOx*)] (4_Cl_) and SiMe_3_OCH_2_SiMe_3_.

After 5 days, the content of the NMR tube was transferred to a 4 mL vial and vapors of *n*-pentane were diffused, affording after 2 days, small orange crystals suitable for X-ray crystallography. Due to the slow conversion, larger scale synthesis of [TiCl(TriNOx*)] (**4_Cl_**) were not pursued.


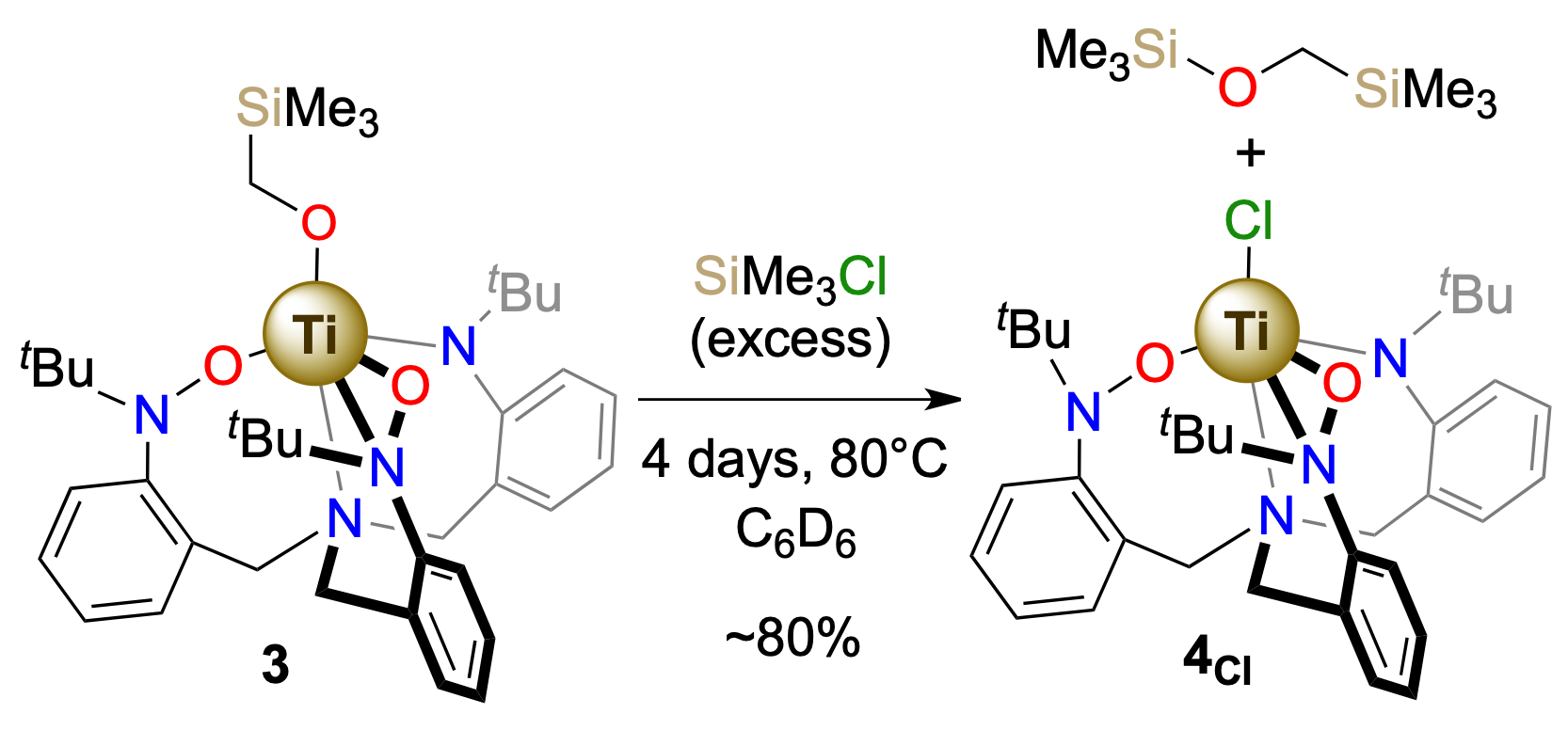


Spectroscopic data for **[**TiCl(TriNOx*)] **(4_Cl_):**

^1^H NMR (C_6_D_6_, 400 MHz, 300 K) δ 7.57 (d, ^3^*J*_HH_= 8.2 Hz, 1H, Ar*H*), 7.54 (d, ^3^*J*_HH_= 7.6 Hz, 1H, Ar*H*), 7.54 (aq, ^3^*J*_HH_= 7.5 Hz, 2H, Ar*H*), 7.09 (d, ^3^*J*_HH_= 7.4 Hz, 1H, Ar*H*), 7.03–6.76 (m, 7H, Ar*H*), 4.59 (d, ^2^*J*_HH_= 11.8 Hz, 1H, NC*H*_2_), 4.42 (d, ^2^*J*_HH_= 12.8 Hz, 1H, NC*H*_2_), 3.59 (d, ^2^*J*_HH_= 10.8 Hz, 1H, NC*H*_2_), 2.45 (d, ^2^*J*_HH_= 12.8 Hz, 1H, NC*H*_2_), 2.39 (d, ^2^*J*_HH_= 11.8 Hz, 1H, NC*H*_2_), 2.28 (d, ^2^*J*_HH_= 10.8 Hz, 1H, NC*H*_2_), 1.11 (s, 9H, C(C*H*_3_)_3_), 1.02 (s, 9H, C(C*H*_3_)_3_), 0.76 (s, 9H, C(C*H*_3_)_3_).

**• Identification of SiMe_3_OCH_2_SiMe_3_:** [Ti(OCH_2_SiMe_3_)(TriNOx*)] (**3**) (5.0 mg, 7 µmol, 1 equiv) and a ferrocene internal standard (1.2 mg) were dissolved in C_6_D_6_ (0.5 mL) and transferred to a J-Young NMR tube. 2 drops of SiMe_3_OTf were added and the tube sealed and immerged in an oil bath preheated at 60 °C for 30 minutes leading to the deposition of a pink precipitate of [Ti(TriNOx*)]OTf (**4_OTf_**). The solid was filtered and the filtrate analyzed by ^1^H NMR spectroscopy as depicted below. The conversion of SiMe_3_OCH_2_SiMe_3_ against the ferrocene internal standard was ~90%. Spectroscopic data for SiMe_3_OCH_2_SiMe_3_ are in agreement with the literature.


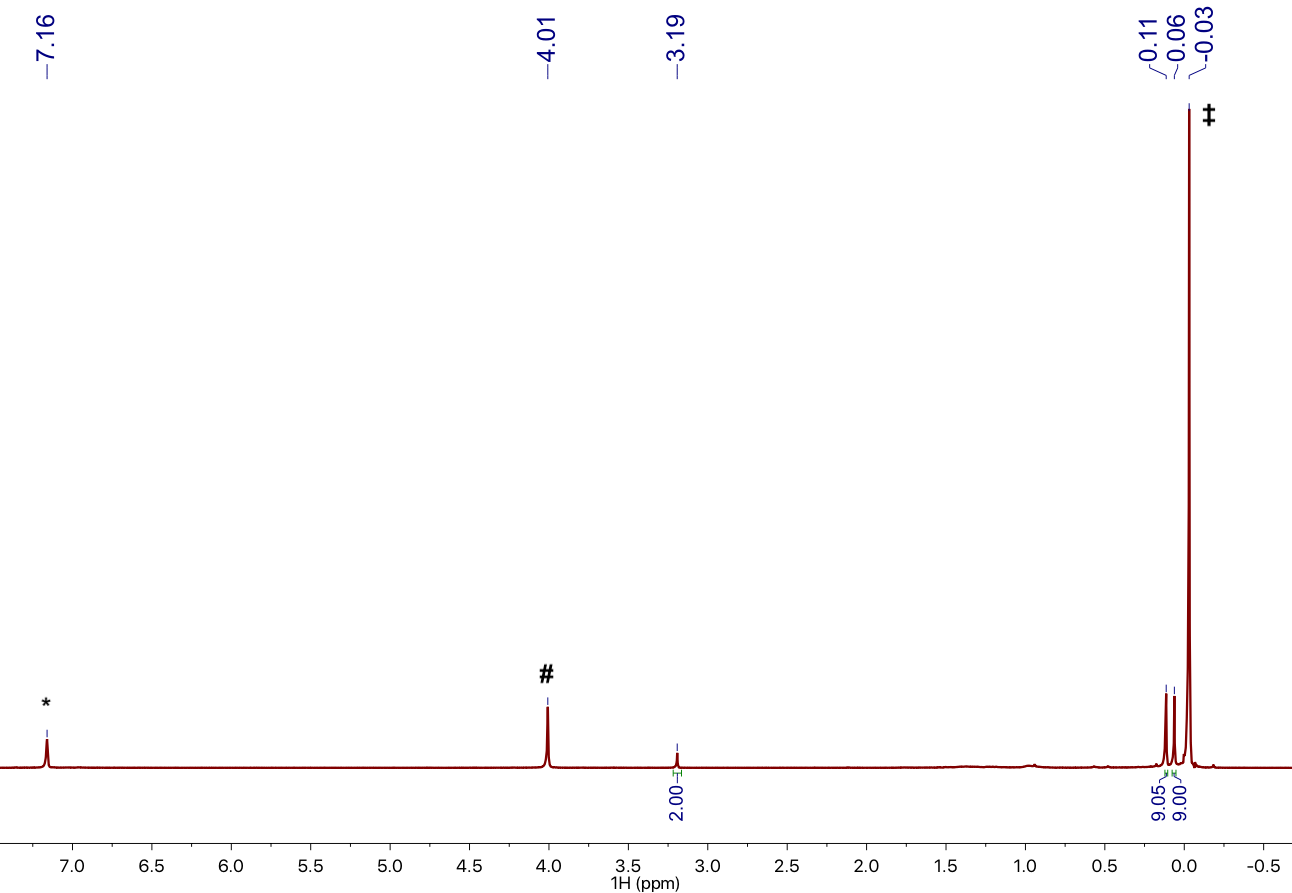


Figure S ^1^H NMR (C_6_D_6_ (*), 400 MHz, 300 K) spectra of the reaction mixture filtrate demonstrating the formation of SiMe_3_CH_2_OSiMe_3_ with a ferrocene internal standard (#) and excess SiMe_3_OTf (‡).

**• Conversion of [Ti(TriNOx*)]OTf (4_OTf_) to [Ti(TriNOx)]OTf (1_OTf_) with NMe_3_O:** To a solution of **4_OTf_** (~12 mg) in CD_2_Cl_2_ was added a few needles of sublimed trimethylamine N-oxide (NMe_3_O) leading to a color change from pink to red. The mixture was charged in a J-Young NMR tube and analyzed by ^1^H NMR spectroscopy. The tube was then heated at 50 °C in an oil bath for 30 minutes during which time, the red color faded to leave a yellow solution.


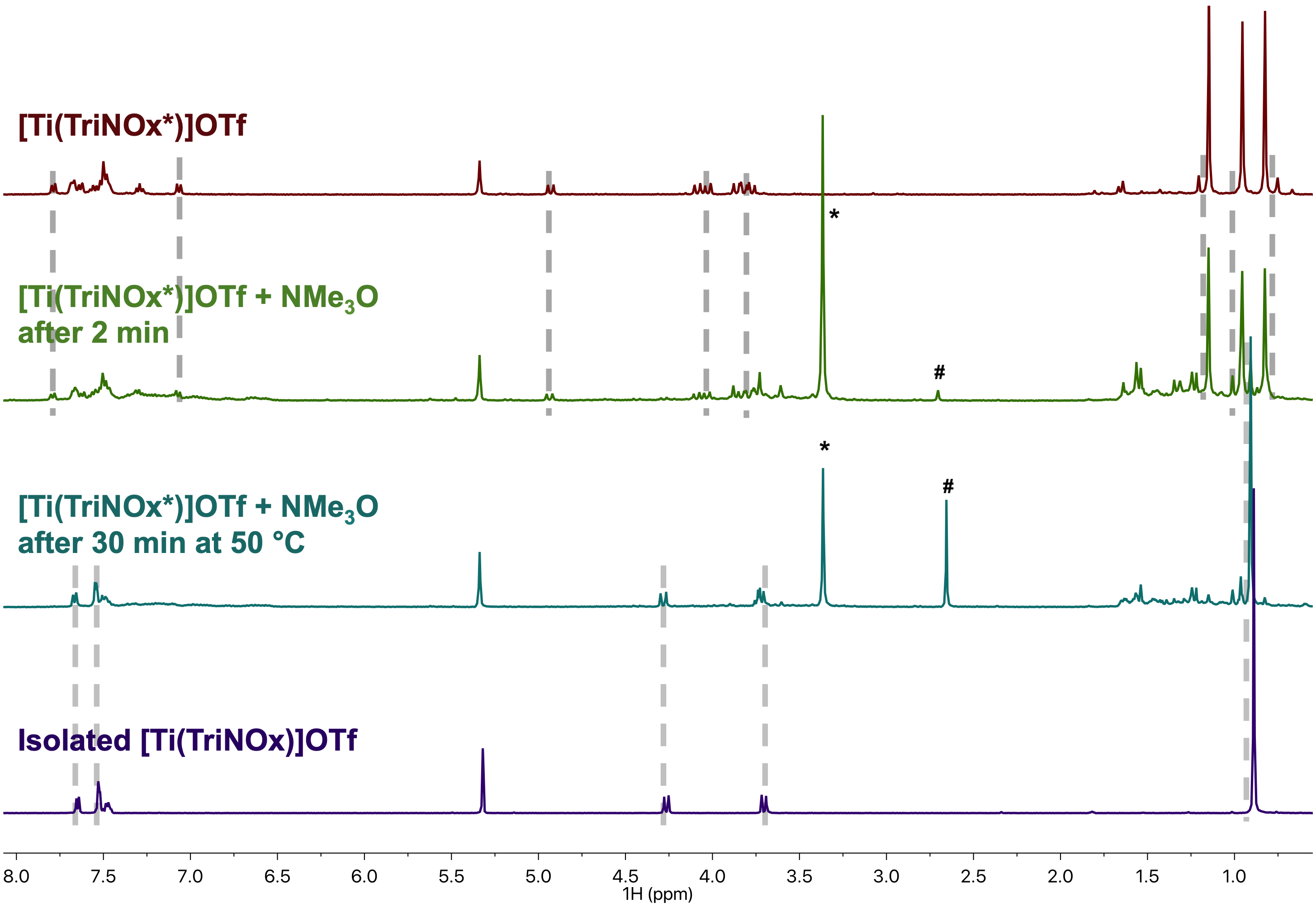


Figure S Formation of [Ti(TriNOx)]OTf (1_OTf_) from [Ti(TriNOx*)]OTf (4_OTf_) and trimethylamine N-oxide (*) and comparison with the original samples. The produced trimethylamine is denoted by #.

**• Conversion of [Ti(TriNOx*)]OTf (4_OTf_) to [Ti(TriNOx)]OTf (1_OTf_) with N-methyl-morpholine N-oxide:** To a solution of **4_OTf_** (~8 mg) in CD_2_Cl_2_ was added a few crystals of freshly sublimed N-methylmorpholine N-oxide (NMO) leading to a color change from pink to red. The mixture was charged in a J-Young NMR tube and analyzed by ^1^H NMR spectroscopy. The tube was then heated at 50 °C in an oil bath for 30 minutes during which time, the red color faded to leave a yellow solution.


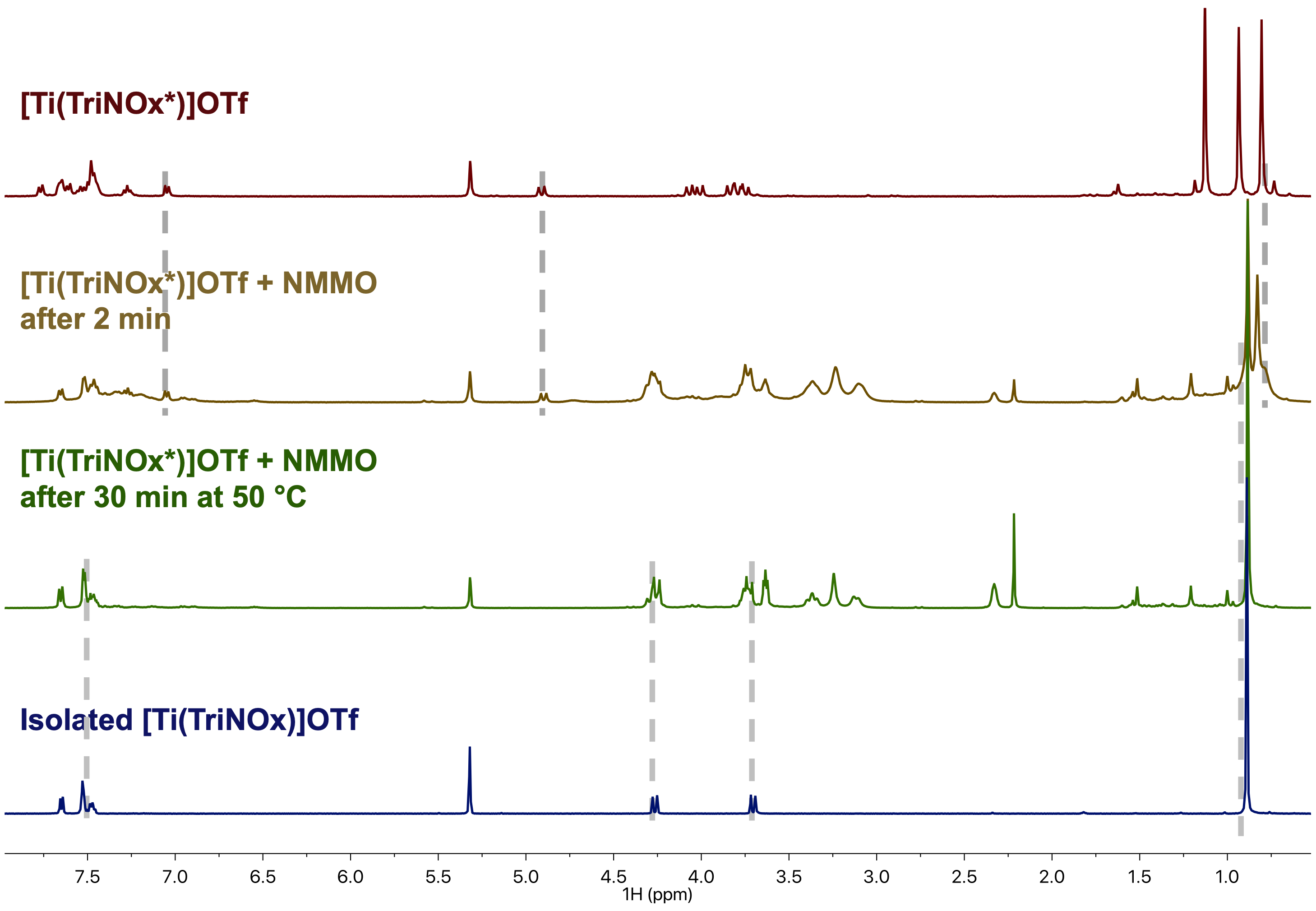


Figure S Formation of [Ti(TriNOx)]OTf (1_OTf_) from [Ti(TriNOx*)]OTf (4_OTf_) and N-methyl-morpholine N-oxide (NMO) and comparison with the original samples.


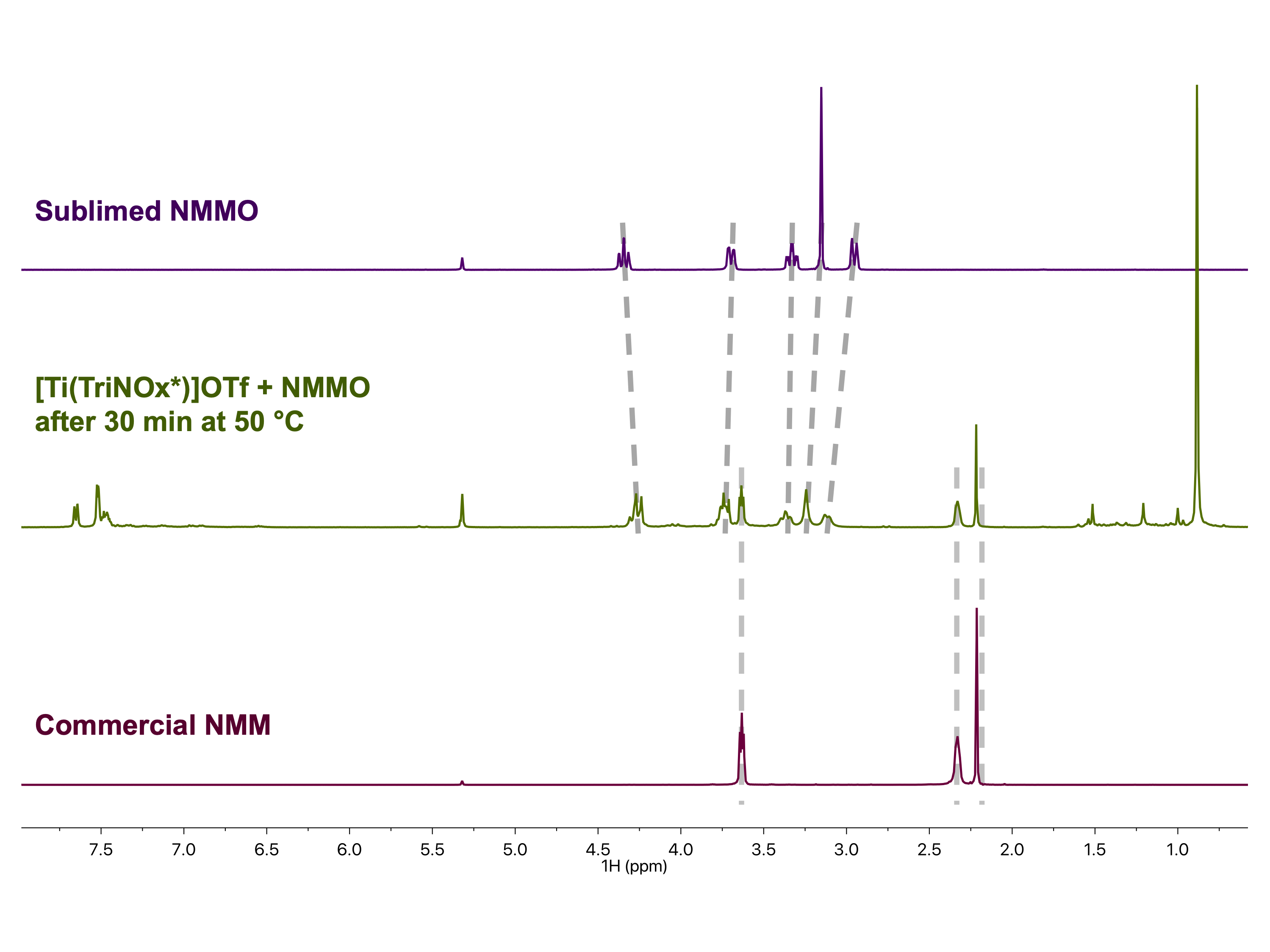


Figure S ^1^H NMR spectrum of the final reaction mixture demonstrating the presence of unreacted N-methyl-morpholine N-oxide (NMO) and the formation of N-methylmorpholine (NMM).

**• Conversion of [Ti(TriNOx)]OTf (1_OTf_) to [Ti(CH_2_SiMe_3_)(TriNOx)] (2):** **1_OTf_** (11.7 mg, 16 µmol, 1 equiv) was suspended in C_6_D_6_ (0.5 mL) with a drop of Et_2_O and LiCH_2_SiMe_3_ (1.5 mg, 16 µmol, 1 equiv) was added as a solid. The solution was agitated for 2 minutes and gradually turned to intense yellow. The precipitate of LiOTf was filtered over a Celite-packed pipette filter and washed with C_6_D_6_(0.2 mL). The filtrate was transferred to a J-Young NMR tube and analyzed by ^1^H NMR spectroscopy as depicted below.


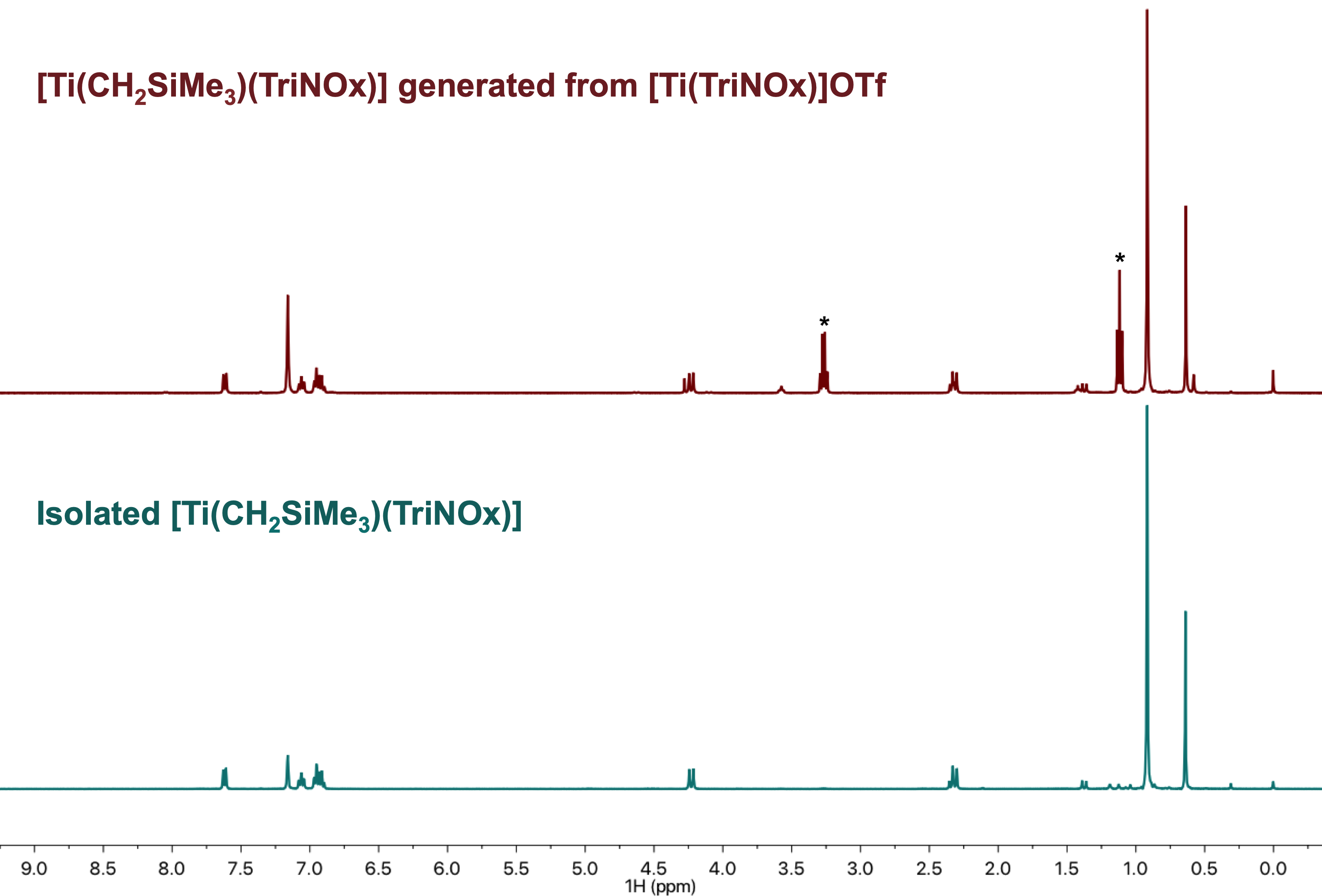


Figure S Formation of [Ti(CH_2_SiMe_3_)(TriNOx)] (2) from [Ti(TriNOx)]OTf (1_OTf_) (top) and comparison with an independently synthesized sample (bottom). * denotes an impurity of Et_2_O.

**• Control reaction between NMO and LiCH_2_SiMe_3_:** LiCH_2_SiMe_3_ (5 mg, 53 μmol) and NMO (6 mg, 51 μmol, 1 equiv) were dissolved in THF-*d_8_* in a J-Young NMR tube and actively mixed overnight at room temperature. White precipitate formed over the course of the reaction, and ^1^H NMR analysis of the mixture indicated the formation of tetramethylsilane as the major product of the reaction. Importantly, no indication of SiMe_3_CH_2_OLi product can be evidenced.

 Figure S ^1^H NMR spectrum of NMO starting material (Top) and the control reaction (Bottom) between LiCH_2_TMS and NMO after 16 h at 298 K in THF-*d_8_* in a J-Young NMR tube.

**X-ray crystallography.**

X-ray intensity data were collected on a Bruker APEXII^3^ CCD area detector or a Bruker APEXIII^4^ D8QUEST CMOS area detector, both employing graphite-monochromated Mo-*K*_α_ radiation (*λ* = 0.71073 Å) at 100(1) K. Rotation frames were integrated using SAINT,^5^ producing a listing of unaveraged *F*^2^ and *σ*(*F*^2^) values which were then passed to the SHELXT program package^6^ for further processing and structure solution. The intensity data were corrected for Lorentz and polarization effects and for absorption using SADABS or TWINABS.^7^ Refinement was performed by full-matrix least squares based on *F*^2^ using SHELXL-2014.^8^ All of the reflections were used during refinement. Non-hydrogen atoms were refined anisotropically and hydrogen atoms were refined using a riding model. Crystallographic parameters are summarized in Table S1.

Table S1*.* Crystallographic Data for 1_OTf_, 2, 3 and 4_Cl_.

| Compounds | **1_OTf_** | **2** | **3** | **4_Cl_** |
| --- | --- | --- | --- | --- |
| Empirical Formula | C_34_H_45_F_3_N_4_O_6_STi | C_37_H_54_N_4_O_3_SiTi | C_37_H_54_N_4_O_3_SiTi | C_33_H_45_N_4_O_2_TiCl |
| Formula Weight | 742.70 | 680.84 | 680.84 | 613.07 |
| *M*_r_ | $R3$ | $P\bar{1}$ | $P2_{1}/n$ | $P2_{1}2_{1}2_{1}$ |
| *a* [Å] | 15.1152(5) | 10.0038(6) | 9.7914(5) | 10.0685(5) |
| *b* [Å] | 15.1152(5) | 12.1930(8) | 19.5177(13) | 17.2900(8) |
| *c* [Å] | 12.8882(10) | 16.8954(11) | 19.616(2) | 19.9278(8) |
| *α* [°] | 90 | 81.798(2) | 90 | 90 |
| *β* [°] | 90 | 78.036(2) | 93.051(4) | 90 |
| *γ* [°] | 120 | 65.845(2) | 90 | 90 |
| *V* [Å^3^] | 2550.1(2) | 1835.7(2) | 3743.4(5) | 3121.1(3) |
| *Z* | 3 | 2 | 4 | 4 |
| *ρ*_calcd_, [g cm^−3^] | 1.451 | 1.232 | 1.208 | 1.305 |
| *μ* [mm^−1^] | 0.381 | 0.306 | 0.300 | 0.396 |
| 2*θ* range [deg] | 6.982 – 54.896 | 5.96 – 55.148 | 5.894 – 55.16 | 6.08 – 53.08 |
| No. unique data | 29062 | 82503 | 180021 | 85259 |
| *R*_int_ | 0.0796 | 0.0557 | 0.0729 | 0.0987 |
| *R_1_* (all data) ^[a]^ | 0.0508 | 0.0488 | 0.0576 | 8.10 |
| *wR_2_* (all data) ^[b]^ | 0.1264 | 0.0906 | 0.1388 | 18.18 |
| GoF ^[c]^ | 1.129 | 1.069 | 1.040 | 1.251 |
| peak/hole [eÅ^−3^] | 1.3/–0.5 | 0.44/–0.51 | 2.01/–0.83 | 0.40/–0.43 |

^[a]^ *R*_1_ = *Σ*||*F*_o_|–|*F*_c_||/*Σ*|*F*_o_|. ^[b]^ *wR*_2_ = [*Σ*[*w*(*F*_o_^2^–*F*_c_^2^)^2^]/*Σ*[*w*(*F*_o_^2^)^2^]]^½^. ^[c]^ Goodness-of-fit [*Σ*[*w*(*F*_o_^2^–*F*_c_^2^)^2^]/(*N*_obs_–*N*_params_)]^½^, based on all data.


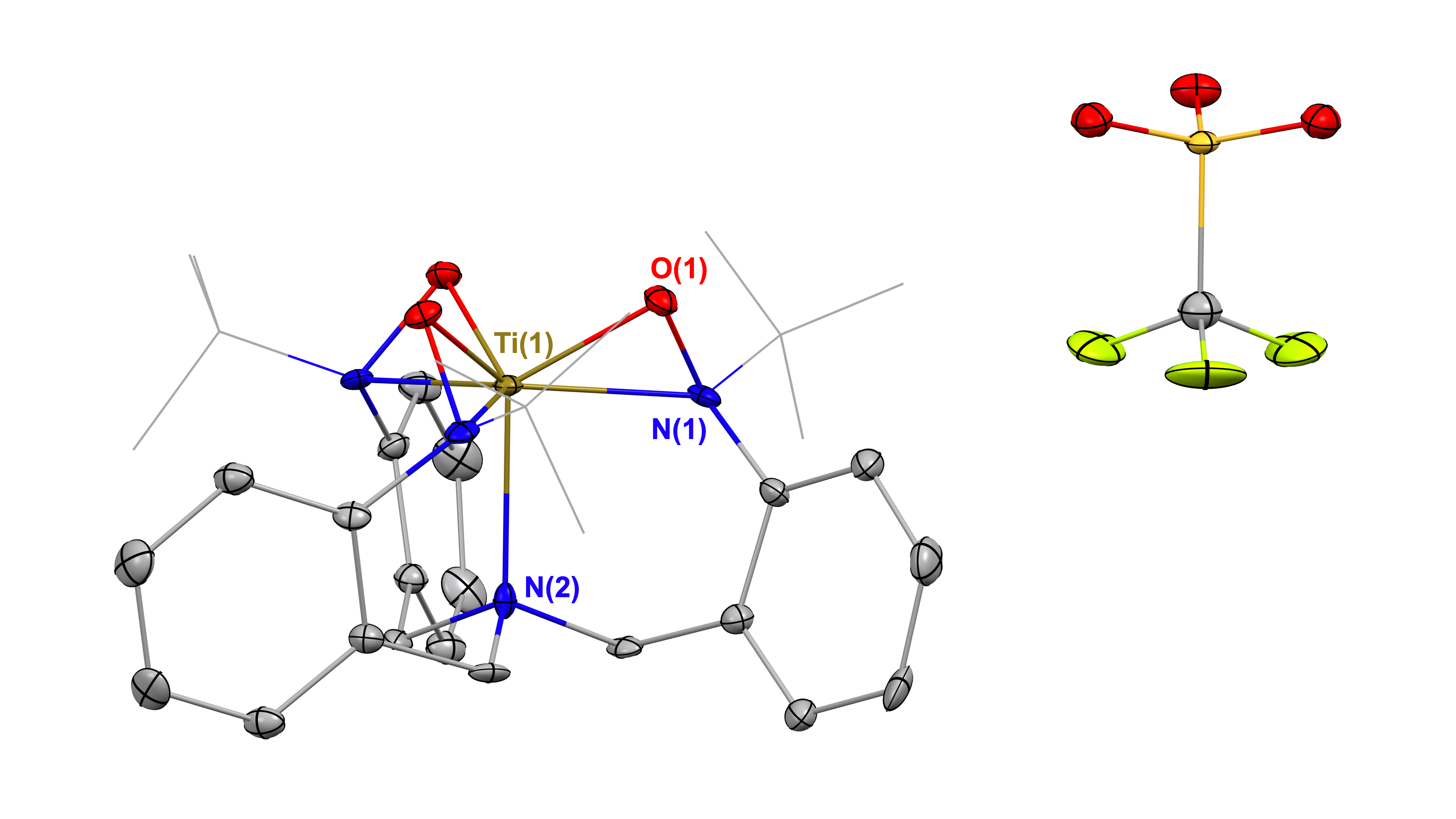


**Figure S** Thermal ellipsoid plot (30% probability) of **1_OTf_**. Hydrogen atoms are omitted, *tert*-butyl groups are depicted with a wireframe model for clarity. Selected bond lengths (Å): Ti(1)–O(1) 1.866(6), Ti(1)–N(1) 2.184(7), Ti(1)–N(2) 2.302(7).


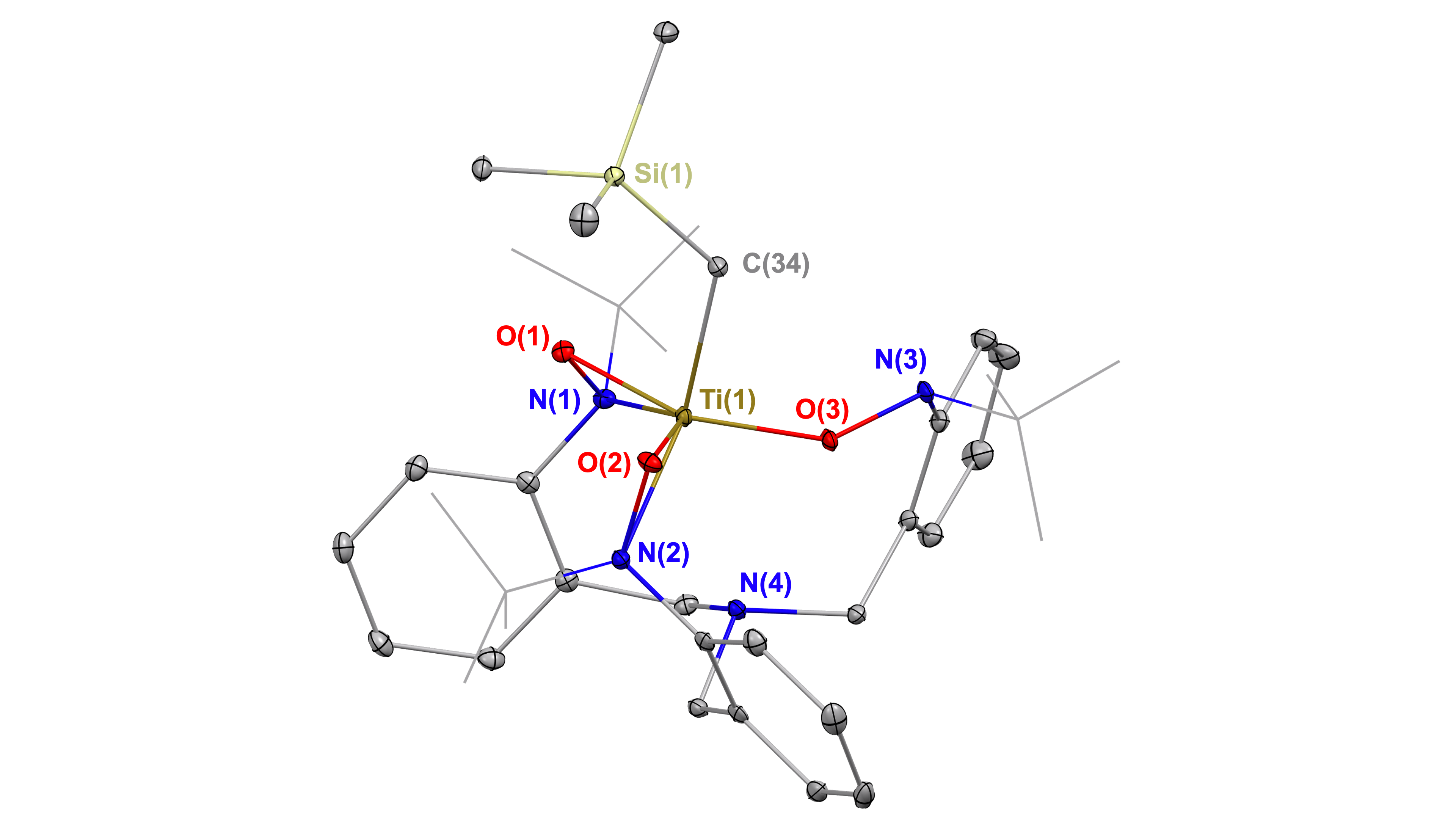


**Figure S** Thermal ellipsoid plot (30% probability) of **2**. Hydrogen atoms are omitted, *tert*-butyl groups are depicted with a wireframe model for clarity. Selected bond lengths (Å): Ti(1)–O(1) 1.8998(11), Ti(1)–O(2) 1.8941(11), Ti(1)–O(3) 1.8274(11), Ti(1)–N(1) 2.3150(13), Ti(1)–N(2) 2.1849(13), Ti(1)–N(4) 3.0649(15), Ti(1)–C(34) 2.1396(16).


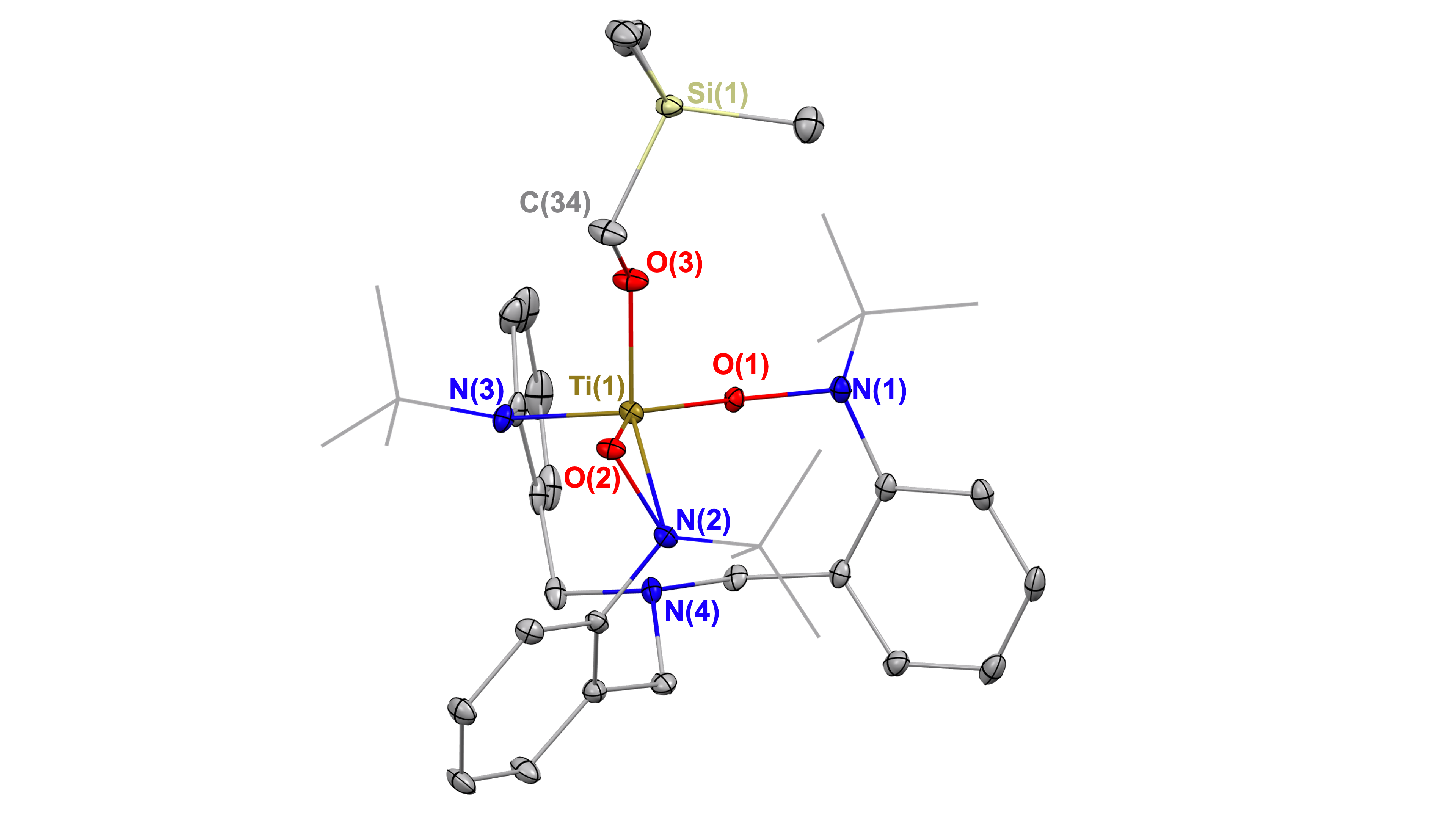


**Figure S** Thermal ellipsoid plot (30% probability) of **3**. Hydrogen atoms are omitted, *tert*-butyl groups are depicted with a wireframe model for clarity. Selected bond lengths (Å): Ti(1)–O(1) 1.8352(15), Ti(1)–O(2) 1.8968(16), Ti(1)–O(3) 1.8437(17), Ti(1)–N(2) 2.2084(19), Ti(1)–N(3) 1.950(2), Ti(1)–N(4) 2.8681(19).


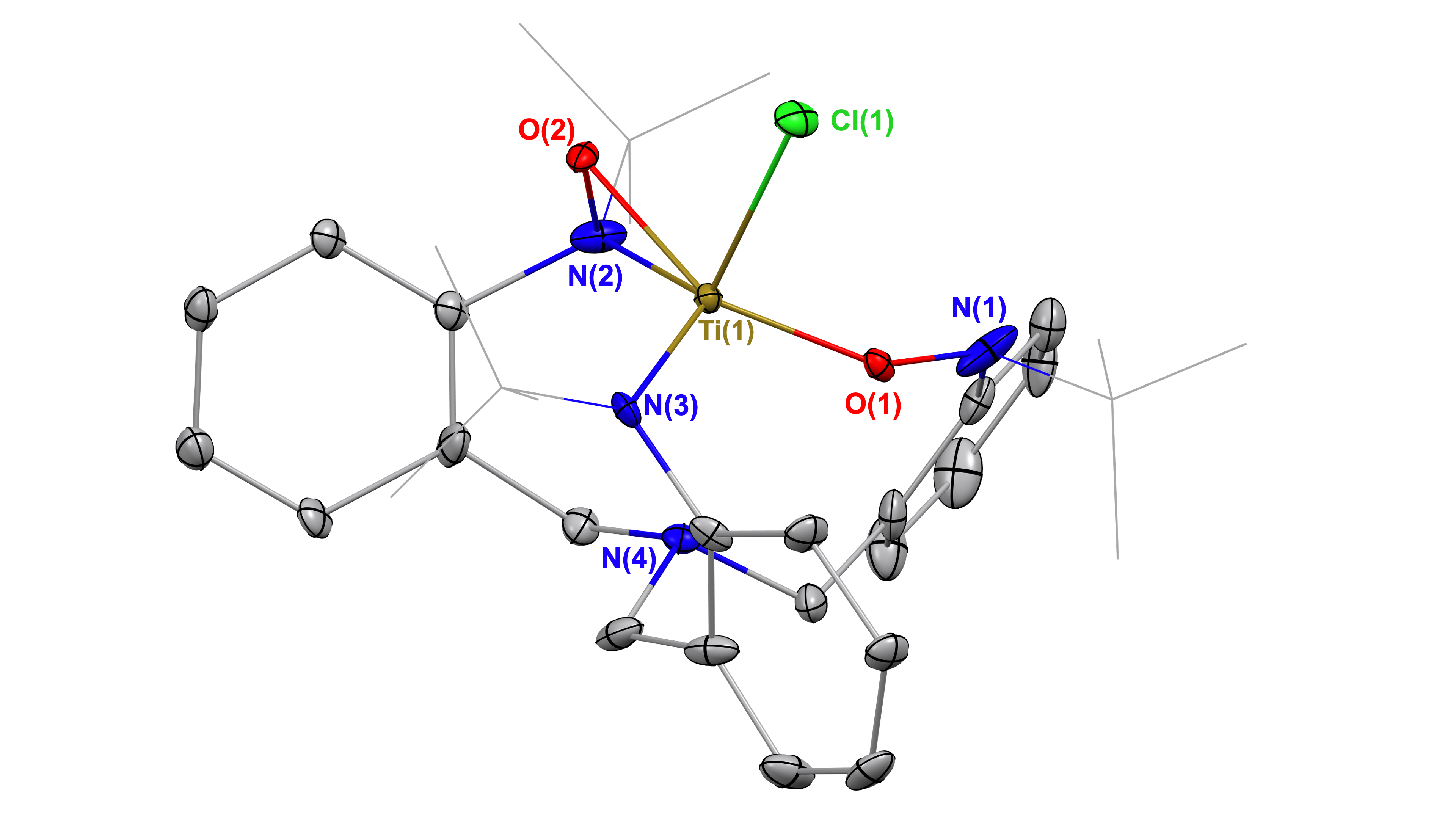


**Figure S** Thermal ellipsoid plot (30% probability) of **4_Cl_**. Hydrogen atoms are omitted, *tert*-butyl groups are depicted with a wireframe model for clarity. Selected bond lengths (Å): Ti(1)–Cl(1) 2.353(3), Ti(1)–O(1) 1.798(7), Ti(1)–O(2) 1.890(6), Ti(1)–N(2) 2.155(5), Ti(1)–N(3) 1.907(9).

Computational Details and Supplementary Data.

Density Functional Theory (DFT) calculations were carried out using the Gaussian ‘09 suite (revision D.01).^9^ The hybrid functional combining Becke’s 3-parameter exchange functional combined with the Lee-Yang-Parr correlation functional (B3LYP) was employed.^10-13^ Dispersion effects were accounted using the D3 empirical correction proposed by Grimme.^14^ Pople’s 6-31G* was employed for light atoms (H, C, N, O, and Si)^15^ while the def2-TZVP basis set was utilized for titanium atoms.^16, 17^ Structures were optimized without constraints; convergence criteria were kept to their default values. All stationary points were verified to possess 0 (reaction intermediates) or 1 imaginary frequency (transition states) by analytical vibrational frequency calculations. Transitions states were confirmed to link reagents and products by intrinsic reaction coordinate (IRC) calculations. Thermochemistry calculations were performed at 298.15 K.

Table S2. Thermochemistry data for computed structures (in Hartrees at 298 K)

| **Compounds** | **SCF**^a^ | **E**^b^ | **H**^c^ | **G**^d^ |
| --- | --- | --- | --- | --- |
| **2** | -3027.320991 | -3026.396895 | -3026.395951 | -3026.524392 |
| **TS1** | -3027.278606 | -3026.356865 | -3026.355921 | -3026.485761 |
| **TS1’** | -3027.272497 | -3026.350376 | -3026.349432 | -3026.477912 |
| **TS1’’** | -3027.232096 | -3026.310525 | -3026.309581 | -3026.44036 |
| **3** | -3027.397674 | -3026.47239 | -3026.471445 | -3026.602259 |
| **4^+^** | -2503.410593 | -2904.705063 | -2904.703175 | -2904.846679 |
| **NMO** | -402.2430038 | -402.067068 | -402.066124 | -402.105221 |
| **4_NMO_^+^** | -2905.690829 | -2904.739483 | -2904.738539 | -2904.866567 |
| **TS2^+^** | -2905.654725 | -2904.70607 | -2904.705126 | -2904.831024 |
| **1^+^** | -2578.614129 | -2577.83595 | -2577.835006 | -2577.940055 |
| **NMM** | -327.1001896 | -326.929781 | -326.928836 | -326.966655 |
| **H_2_NO^−^** | -131.0427544 | -131.016191 | -131.015247 | -131.041174 |
| **HOO^−^** | -150.8869847 | -150.871533 | -150.870589 | -150.896301 |

a) SCF Energies from single-point; b) Sum of electronic and thermal Energies; c) Sum of electronic and thermal Enthalpies; d) Sum of electronic and thermal Free Energies.

**Table S3.** Lowest frequencies (cm^-1^) of computed structures.

| **Compounds** | Freq 1 | Freq 2 | Freq 3 |
| --- | --- | --- | --- |
| **2** | 24.9167 | 36.667 | 39.8491 |
| **TS1** | -512.3400 | 20.6868 | 33.7401 |
| **TS1’** | -474.9911 | 28.1952 | 29.0060 |
| **TS1’’** | -117.8461 | 15.0707 | 30.7361 |
| **3** | 26.7202 | 28.6523 | 35.9554 |
| **4^+^** | 29.1646 | 32.2432 | 39.0379 |
| **NMO** | 179.9784 | 245.6674 | 263.3803 |
| **4_NMO_^+^** | 15.6000 | 22.9214 | 29.3456 |
| **TS2^+^** | -336.3161 | 27.1513 | 32.3545 |
| **1^+^** | 43.2615 | 45.0589 | 47.0808 |
| **NMM** | 162.1307 | 227.9400 | 272.7244 |
| **H_2_NO^−^** | 852.4047 | 1218.0072 | 1261.6599 |
| **HOO^−^** | 760.6106 | 1085.8495 | 3641.3020 |

**Table S4.** Atomic coordinates of computed structures.

**2**

| Ti -0.648488000 -0.145034000 -0.664422000 |
| --- |
| O 0.115761000 -1.262752000 -1.993982000 |
| O -2.225479000 -0.804192000 0.145595000 |
| O -0.259070000 1.469571000 0.122506000 |
| N 1.456480000 -0.959712000 -1.583579000 |
| N -1.340301000 -1.541936000 0.994883000 |
| N 0.248877000 2.763081000 -0.070915000 |
| N 1.689251000 -0.314273000 1.510318000 |
| C 2.948451000 -0.836803000 0.962339000 |
| H 3.687605000 -1.015220000 1.768837000 |
| H 3.368936000 -0.066185000 0.320065000 |
| C 2.771843000 -2.118272000 0.178051000 |
| C 3.340488000 -3.303833000 0.667788000 |
| H 3.912680000 -3.260987000 1.591773000 |
| C 3.177075000 -4.523728000 0.013952000 |
| H 3.627674000 -5.425362000 0.419582000 |
| C 2.411444000 -4.574563000 -1.151135000 |
| H 2.252685000 -5.518541000 -1.665557000 |
| C 1.845585000 -3.407007000 -1.660084000 |
| H 1.231620000 -3.444931000 -2.550982000 |
| C 2.039883000 -2.171928000 -1.024251000 |
| C 2.210930000 -0.368070000 -2.772881000 |
| C 1.481675000 0.910398000 -3.211010000 |
| H 2.096658000 1.439228000 -3.948230000 |
| H 1.318532000 1.586913000 -2.367240000 |
| H 0.518172000 0.686726000 -3.671967000 |
| C 2.272126000 -1.339462000 -3.967514000 |
| H 2.679880000 -0.810950000 -4.836542000 |
| H 1.271640000 -1.696231000 -4.228140000 |
| H 2.918846000 -2.199785000 -3.770827000 |
| C 3.636200000 -0.001788000 -2.329586000 |
| H 4.180643000 -0.864472000 -1.932904000 |
| H 3.631930000 0.796278000 -1.581994000 |
| H 4.190579000 0.364478000 -3.200536000 |
| C 1.188208000 -1.209966000 2.566088000 |
| H 1.891531000 -1.225804000 3.423037000 |
| H 1.180669000 -2.217021000 2.152307000 |
| C -0.182209000 -0.830552000 3.089360000 |
| C -0.280199000 -0.292958000 4.382320000 |
| H 0.626011000 -0.212384000 4.977926000 |
| C -1.489150000 0.158793000 4.906238000 |
| H -1.527619000 0.574924000 5.909101000 |
| C -2.638772000 0.095604000 4.118646000 |
| H -3.587998000 0.465711000 4.496430000 |
| C -2.570835000 -0.449720000 2.838701000 |
| H -3.453131000 -0.487488000 2.212722000 |
| C -1.362313000 -0.943438000 2.328525000 |
| C -1.759804000 -3.023402000 0.948945000 |
| C -1.722082000 -3.502502000 -0.508000000 |
| H -0.734631000 -3.363866000 -0.948644000 |
| H -2.451937000 -2.974797000 -1.124005000 |
| H -1.963681000 -4.571312000 -0.530127000 |
| C -3.188606000 -3.203802000 1.496449000 |
| H -3.499560000 -4.241891000 1.336260000 |
| H -3.893011000 -2.555334000 0.968570000 |
| H -3.253050000 -2.999639000 2.569060000 |
| C -0.780583000 -3.867535000 1.777050000 |
| H -1.133823000 -4.904488000 1.780136000 |
| H -0.725558000 -3.537714000 2.818941000 |
| H 0.222700000 -3.864226000 1.341724000 |
| C 1.842244000 1.057187000 2.042885000 |
| H 2.503827000 1.035756000 2.931518000 |
| H 0.860398000 1.370224000 2.388457000 |
| C 2.417520000 2.081035000 1.080649000 |
| C 3.799026000 2.330256000 1.163669000 |
| H 4.372036000 1.781124000 1.907588000 |
| C 4.454169000 3.249047000 0.347857000 |
| H 5.523458000 3.409321000 0.453016000 |
| C 3.716669000 3.944324000 -0.609953000 |
| H 4.201581000 4.648725000 -1.280436000 |
| C 2.348549000 3.716014000 -0.717702000 |
| H 1.769797000 4.227250000 -1.478525000 |
| C 1.669281000 2.825931000 0.135549000 |
| C -0.618301000 3.762269000 0.664653000 |
| C -2.035200000 3.643384000 0.086493000 |
| H -2.028081000 3.841291000 -0.990366000 |
| H -2.693149000 4.375324000 0.568990000 |
| H -2.446402000 2.647638000 0.250796000 |
| C -0.102677000 5.187061000 0.398654000 |
| H 0.891487000 5.361802000 0.819158000 |
| H -0.793240000 5.895875000 0.868295000 |
| H -0.073396000 5.408408000 -0.672964000 |
| C -0.652349000 3.510808000 2.184323000 |
| H -1.034873000 2.511521000 2.408962000 |
| H -1.314360000 4.239681000 2.666110000 |
| H 0.342102000 3.623912000 2.630362000 |
| C -1.931458000 0.691472000 -2.175037000 |
| H -1.593292000 1.738345000 -2.212378000 |
| H -1.563966000 0.208373000 -3.092037000 |
| Si -3.808205000 0.615459000 -2.295746000 |
| C -4.748488000 1.265512000 -0.777103000 |
| H -4.399344000 0.778140000 0.139378000 |
| H -4.631771000 2.348138000 -0.651511000 |
| H -5.822546000 1.062898000 -0.882309000 |
| C -4.375253000 1.662574000 -3.787313000 |
| H -3.927200000 1.302668000 -4.722269000 |
| H -5.466185000 1.631361000 -3.909502000 |
| H -4.085869000 2.714800000 -3.669945000 |
| C -4.384073000 -1.168221000 -2.613250000 |
| H -5.443737000 -1.196369000 -2.898097000 |
| H -3.810942000 -1.639325000 -3.422035000 |
| H -4.263643000 -1.784063000 -1.715381000 |
| **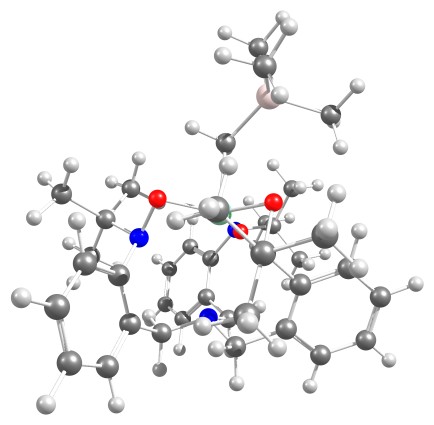**  **TS1**  Ti 0.483113000 -0.407475000 -0.301767000 |
| Si 2.510785000 -2.007762000 -3.129100000 |
| O 1.274488000 1.008593000 -1.269021000 |
| O 1.623464000 -1.700041000 -0.119256000 |
| O -1.221007000 -1.133869000 -0.230252000 |
| N 0.346449000 2.063032000 -0.950688000 |
| N 1.589667000 -0.740946000 1.567862000 |
| N -2.441893000 -1.254829000 -0.905468000 |
| N -1.034257000 1.055956000 1.680587000 |
| C -1.075722000 2.523621000 1.493255000 |
| H -1.540162000 3.004889000 2.374292000 |
| H -1.725191000 2.717463000 0.642374000 |
| C 0.278170000 3.146254000 1.246777000 |
| C 0.867276000 3.965754000 2.219735000 |
| H 0.324292000 4.157325000 3.142513000 |
| C 2.131470000 4.522860000 2.031584000 |
| H 2.569764000 5.153074000 2.800538000 |
| C 2.834866000 4.245397000 0.858340000 |
| H 3.831959000 4.650441000 0.707476000 |
| C 2.259814000 3.441156000 -0.124924000 |
| H 2.817988000 3.197409000 -1.019995000 |
| C 0.972014000 2.913606000 0.044874000 |
| C -0.104967000 2.734597000 -2.229781000 |
| C -0.763436000 1.663236000 -3.112136000 |
| H -1.251215000 2.144349000 -3.967604000 |
| H -1.525639000 1.108980000 -2.557492000 |
| H -0.023183000 0.959129000 -3.499039000 |
| C 1.047982000 3.385122000 -3.020774000 |
| H 0.678484000 3.698346000 -4.003896000 |
| H 1.861482000 2.670843000 -3.178375000 |
| H 1.443605000 4.274240000 -2.521018000 |
| C -1.151588000 3.800530000 -1.864017000 |
| H -0.750966000 4.545860000 -1.169087000 |
| H -2.046298000 3.348795000 -1.424757000 |
| H -1.457229000 4.328671000 -2.773975000 |
| C -0.402158000 0.741319000 2.987143000 |
| H -1.018850000 1.154169000 3.807423000 |
| H 0.557926000 1.257843000 3.007835000 |
| C -0.204243000 -0.739122000 3.215440000 |
| C -0.988804000 -1.416943000 4.155741000 |
| H -1.715100000 -0.852528000 4.736692000 |
| C -0.864938000 -2.792328000 4.353612000 |
| H -1.486530000 -3.296302000 5.088558000 |
| C 0.044972000 -3.513664000 3.578910000 |
| H 0.129290000 -4.591264000 3.693569000 |
| C 0.846863000 -2.854969000 2.648809000 |
| H 1.526871000 -3.417234000 2.021590000 |
| C 0.766274000 -1.460314000 2.476709000 |
| C 3.050119000 -0.617098000 1.894749000 |
| C 3.775517000 0.119764000 0.760113000 |
| H 3.291500000 1.071217000 0.533524000 |
| H 3.800510000 -0.481252000 -0.150544000 |
| H 4.807823000 0.323865000 1.067692000 |
| C 3.762414000 -1.964599000 2.137861000 |
| H 4.833219000 -1.782838000 2.286962000 |
| H 3.642837000 -2.625148000 1.274810000 |
| H 3.389913000 -2.474822000 3.030675000 |
| C 3.163403000 0.240011000 3.178881000 |
| H 4.214768000 0.309207000 3.481994000 |
| H 2.605299000 -0.203038000 4.010449000 |
| H 2.795837000 1.256858000 3.007457000 |
| C -2.415268000 0.498105000 1.678778000 |
| H -2.967987000 0.929619000 2.534084000 |
| H -2.319329000 -0.567211000 1.866695000 |
| C -3.250999000 0.741879000 0.434254000 |
| C -4.134581000 1.835342000 0.454793000 |
| H -4.162301000 2.453815000 1.349283000 |
| C -4.980234000 2.145666000 -0.607672000 |
| H -5.648283000 2.999887000 -0.543741000 |
| C -4.944733000 1.348637000 -1.751473000 |
| H -5.572968000 1.579222000 -2.607663000 |
| C -4.080087000 0.259779000 -1.801895000 |
| H -4.017746000 -0.343586000 -2.700120000 |
| C -3.254309000 -0.086436000 -0.716223000 |
| C -3.059599000 -2.603643000 -0.600433000 |
| C -2.040926000 -3.680476000 -1.004630000 |
| H -1.774673000 -3.580215000 -2.061768000 |
| H -2.479929000 -4.672990000 -0.851517000 |
| H -1.130587000 -3.609734000 -0.406489000 |
| C -4.325378000 -2.789730000 -1.454820000 |
| H -5.120558000 -2.089231000 -1.185637000 |
| H -4.707357000 -3.803876000 -1.295317000 |
| H -4.104464000 -2.677591000 -2.521304000 |
| C -3.421842000 -2.766250000 0.888474000 |
| H -2.532473000 -2.700091000 1.520849000 |
| H -3.877112000 -3.749425000 1.055339000 |
| H -4.146608000 -2.008391000 1.206932000 |
| C 0.902868000 -1.804352000 -2.180770000 |
| H 0.416417000 -2.749943000 -1.948860000 |
| H 0.173174000 -1.143274000 -2.668542000 |
| C 2.104601000 -2.780263000 -4.824816000 |
| H 1.436843000 -2.136253000 -5.410657000 |
| H 1.610587000 -3.753622000 -4.714496000 |
| H 3.016139000 -2.936981000 -5.416647000 |
| C 3.384119000 -0.355263000 -3.446279000 |
| H 2.768241000 0.300791000 -4.073503000 |
| H 4.337007000 -0.514300000 -3.967615000 |
| H 3.586383000 0.181351000 -2.514074000 |
| C 3.680311000 -3.192833000 -2.222558000 |
| H 4.570685000 -3.409040000 -2.826497000 |
| H 3.183750000 -4.147410000 -2.008172000 |
| H 4.009903000 -2.773005000 -1.266540000  **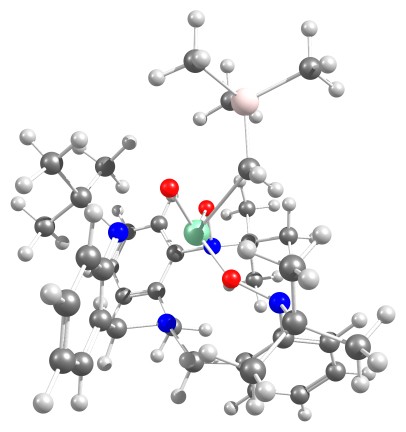** |

**TS1’**

| Ti 0.510709000 0.278664000 -0.416918000 |
| --- |
| Si 2.850688000 3.028088000 -1.976219000 |
| O 1.574655000 1.494608000 0.250708000 |
| O 1.413527000 -0.755460000 -1.745541000 |
| O -1.235113000 0.424655000 -0.985583000 |
| N 0.594317000 0.881839000 1.800539000 |
| N 1.247173000 -1.870187000 -0.879770000 |
| N -2.357760000 1.263822000 -1.055335000 |
| N -1.200924000 -1.573936000 1.152947000 |
| C -1.040020000 -1.306398000 2.595439000 |
| H -1.545097000 -2.088191000 3.194727000 |
| H -1.540463000 -0.364035000 2.806945000 |
| C 0.409546000 -1.213143000 3.018230000 |
| C 0.969612000 -2.187530000 3.851407000 |
| H 0.345390000 -3.015285000 4.182135000 |
| C 2.299715000 -2.115452000 4.271245000 |
| H 2.710620000 -2.884890000 4.919014000 |
| C 3.093175000 -1.052139000 3.840373000 |
| H 4.136420000 -0.990198000 4.139198000 |
| C 2.551956000 -0.063579000 3.018619000 |
| H 3.177067000 0.746236000 2.662131000 |
| C 1.206159000 -0.110128000 2.608212000 |
| C 0.361376000 2.225380000 2.440672000 |
| C -0.315306000 3.172615000 1.435919000 |
| H -0.719299000 4.038044000 1.974827000 |
| H -1.145151000 2.676883000 0.928392000 |
| H 0.386733000 3.533895000 0.684570000 |
| C 1.639246000 2.906349000 2.976892000 |
| H 1.393202000 3.922812000 3.306446000 |
| H 2.397743000 2.974779000 2.193163000 |
| H 2.061800000 2.378796000 3.837273000 |
| C -0.611692000 2.023528000 3.628570000 |
| H -0.237942000 1.282065000 4.342285000 |
| H -1.604028000 1.718517000 3.283039000 |
| H -0.724262000 2.972520000 4.165854000 |
| C -0.814232000 -2.974551000 0.885762000 |
| H -1.526931000 -3.664336000 1.377348000 |
| H 0.153704000 -3.132224000 1.359285000 |
| C -0.749562000 -3.310681000 -0.585288000 |
| C -1.706152000 -4.170306000 -1.144873000 |
| H -2.447177000 -4.621781000 -0.489320000 |
| C -1.742911000 -4.432138000 -2.512750000 |
| H -2.497996000 -5.098541000 -2.920388000 |
| C -0.821287000 -3.806145000 -3.353699000 |
| H -0.852713000 -3.974839000 -4.426715000 |
| C 0.147060000 -2.960710000 -2.816137000 |
| H 0.851457000 -2.452140000 -3.462130000 |
| C 0.212251000 -2.737771000 -1.434284000 |
| C 2.614076000 -2.541610000 -0.681285000 |
| C 3.589037000 -1.485446000 -0.149785000 |
| H 3.207820000 -1.016888000 0.759796000 |
| H 3.778540000 -0.708243000 -0.890461000 |
| H 4.540791000 -1.970694000 0.095487000 |
| C 3.158002000 -3.096448000 -2.011801000 |
| H 4.184311000 -3.446548000 -1.855773000 |
| H 3.179784000 -2.316388000 -2.777807000 |
| H 2.571152000 -3.942698000 -2.380311000 |
| C 2.489474000 -3.681861000 0.339134000 |
| H 3.456597000 -4.191422000 0.412151000 |
| H 1.748604000 -4.428242000 0.035668000 |
| H 2.239858000 -3.307025000 1.335877000 |
| C -2.595790000 -1.362001000 0.686230000 |
| H -3.245437000 -2.124950000 1.155588000 |
| H -2.596028000 -1.562508000 -0.382321000 |
| C -3.236263000 -0.011036000 0.967348000 |
| C -4.051308000 0.070427000 2.111049000 |
| H -4.144384000 -0.816460000 2.734020000 |
| C -4.751891000 1.220033000 2.465897000 |
| H -5.370547000 1.230652000 3.358747000 |
| C -4.635381000 2.352620000 1.661194000 |
| H -5.149422000 3.273995000 1.921414000 |
| C -3.828972000 2.308030000 0.529025000 |
| H -3.693299000 3.198414000 -0.073839000 |
| C -3.147304000 1.139347000 0.140696000 |
| C -3.038258000 1.064326000 -2.391554000 |
| C -2.002804000 1.385829000 -3.481423000 |
| H -1.607893000 2.398711000 -3.351464000 |
| H -2.477287000 1.323367000 -4.467332000 |
| H -1.167412000 0.683688000 -3.455438000 |
| C -4.206844000 2.055430000 -2.523873000 |
| H -5.003837000 1.863219000 -1.800357000 |
| H -4.636011000 1.956668000 -3.526844000 |
| H -3.866333000 3.089153000 -2.403930000 |
| C -3.562188000 -0.372651000 -2.577974000 |
| H -2.745065000 -1.097749000 -2.534383000 |
| H -4.045206000 -0.470009000 -3.557253000 |
| H -4.307009000 -0.625822000 -1.814988000 |
| C 1.286680000 1.991427000 -1.844397000 |
| H 1.206960000 1.284236000 -2.676572000 |
| H 0.379072000 2.595788000 -1.760733000 |
| C 2.910995000 3.733063000 -3.746458000 |
| H 2.029072000 4.348343000 -3.964152000 |
| H 2.945690000 2.931711000 -4.495234000 |
| H 3.798337000 4.362796000 -3.894019000 |
| C 2.841379000 4.498172000 -0.779601000 |
| H 1.926447000 5.094758000 -0.884052000 |
| H 3.691043000 5.163535000 -0.979345000 |
| H 2.908341000 4.171553000 0.263001000 |
| C 4.424931000 2.006337000 -1.713857000 |
| H 5.320042000 2.613317000 -1.900805000 |
| H 4.459018000 1.148277000 -2.396275000 |
| H 4.482145000 1.623210000 -0.689480000  **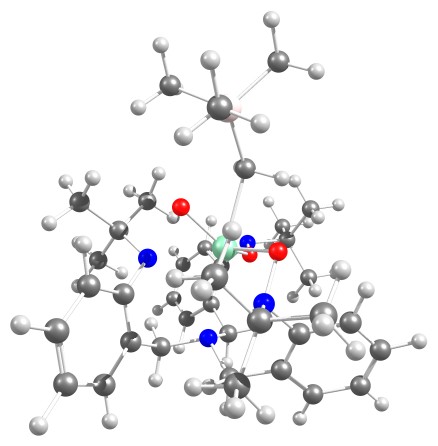** |

**TS1’’**

| Ti -0.534400000 -0.402470000 0.048765000 |
| --- |
| Si -2.884419000 -3.393225000 0.424011000 |
| O -1.183714000 -0.567554000 1.831048000 |
| O -2.099255000 -0.507137000 -1.057278000 |
| O 0.672184000 -1.565908000 -0.432076000 |
| N -0.007923000 0.175871000 2.143185000 |
| N -2.037337000 0.913020000 -1.150814000 |
| N 2.870955000 -1.636166000 -0.369694000 |
| N 0.931802000 1.588355000 -0.513189000 |
| C 1.442859000 2.204784000 0.752447000 |
| H 2.015704000 3.112886000 0.513222000 |
| H 2.139931000 1.498593000 1.191459000 |
| C 0.357224000 2.559920000 1.741630000 |
| C 0.046629000 3.901330000 2.006860000 |
| H 0.607045000 4.681468000 1.496815000 |
| C -0.967382000 4.247318000 2.899365000 |
| H -1.192310000 5.293198000 3.088495000 |
| C -1.701723000 3.241450000 3.531222000 |
| H -2.509615000 3.497281000 4.211207000 |
| C -1.399050000 1.901788000 3.291193000 |
| H -1.980029000 1.115882000 3.757779000 |
| C -0.355639000 1.558292000 2.422978000 |
| C 0.793220000 -0.554353000 3.230051000 |
| C 1.115842000 -1.971662000 2.737252000 |
| H 1.744464000 -2.460972000 3.490865000 |
| H 1.651524000 -1.963826000 1.784423000 |
| H 0.203729000 -2.561710000 2.622273000 |
| C -0.023520000 -0.644519000 4.533513000 |
| H 0.504338000 -1.298113000 5.236306000 |
| H -1.009589000 -1.078122000 4.343737000 |
| H -0.145513000 0.328713000 5.018878000 |
| C 2.104158000 0.202775000 3.491576000 |
| H 1.936139000 1.252088000 3.755782000 |
| H 2.782100000 0.146716000 2.635423000 |
| H 2.612303000 -0.269441000 4.339311000 |
| C 0.179851000 2.656230000 -1.248611000 |
| H 0.874937000 3.479216000 -1.478970000 |
| H -0.573884000 3.047769000 -0.568688000 |
| C -0.447568000 2.160308000 -2.524542000 |
| C 0.055903000 2.532110000 -3.778252000 |
| H 0.870960000 3.250060000 -3.830001000 |
| C -0.454347000 1.975333000 -4.949141000 |
| H -0.049456000 2.269659000 -5.913246000 |
| C -1.462998000 1.011649000 -4.871515000 |
| H -1.841859000 0.542637000 -5.775385000 |
| C -1.988495000 0.647027000 -3.633436000 |
| H -2.751425000 -0.118087000 -3.563637000 |
| C -1.512798000 1.252144000 -2.463182000 |
| C -3.387612000 1.542812000 -0.804843000 |
| C -3.835091000 1.019269000 0.564883000 |
| H -3.079915000 1.206504000 1.329576000 |
| H -4.036566000 -0.052477000 0.537287000 |
| H -4.756255000 1.536738000 0.855944000 |
| C -4.467217000 1.196370000 -1.848653000 |
| H -5.437168000 1.557306000 -1.489106000 |
| H -4.544022000 0.115124000 -1.991574000 |
| H -4.277064000 1.669240000 -2.816414000 |
| C -3.217802000 3.069057000 -0.730750000 |
| H -4.198640000 3.525867000 -0.559531000 |
| H -2.819410000 3.485815000 -1.661430000 |
| H -2.568996000 3.361621000 0.100794000 |
| C 2.076715000 1.128705000 -1.419344000 |
| H 2.276479000 1.941167000 -2.127908000 |
| H 1.701010000 0.279290000 -1.983223000 |
| C 3.371515000 0.797843000 -0.726527000 |
| C 4.306158000 1.831394000 -0.541531000 |
| H 4.086717000 2.808242000 -0.969772000 |
| C 5.488340000 1.652226000 0.168515000 |
| H 6.190882000 2.471997000 0.287544000 |
| C 5.736008000 0.401890000 0.743490000 |
| H 6.634073000 0.236825000 1.334309000 |
| C 4.834027000 -0.638877000 0.563872000 |
| H 5.022904000 -1.608752000 1.011486000 |
| C 3.656946000 -0.501603000 -0.226337000 |
| C 3.112945000 -2.547701000 -1.506631000 |
| C 2.385240000 -3.872945000 -1.219540000 |
| H 2.712660000 -4.277672000 -0.255466000 |
| H 2.611693000 -4.610470000 -1.999889000 |
| H 1.308031000 -3.720420000 -1.179005000 |
| C 4.630544000 -2.860217000 -1.625024000 |
| H 5.219338000 -1.968741000 -1.860960000 |
| H 4.780230000 -3.591044000 -2.428415000 |
| H 5.017608000 -3.294337000 -0.697460000 |
| C 2.644896000 -1.983565000 -2.868992000 |
| H 1.570532000 -1.782898000 -2.843396000 |
| H 2.843497000 -2.706980000 -3.669714000 |
| H 3.179281000 -1.060054000 -3.121240000 |
| C -1.164117000 -2.887310000 -0.176034000 |
| H -1.036818000 -3.023718000 -1.250204000 |
| H -0.374983000 -3.411756000 0.359448000 |
| C -2.800243000 -5.300267000 0.543821000 |
| H -2.039223000 -5.624949000 1.263704000 |
| H -2.557402000 -5.755782000 -0.423514000 |
| H -3.766201000 -5.707714000 0.872644000 |
| C -3.373921000 -2.767602000 2.144260000 |
| H -2.634772000 -3.071241000 2.894945000 |
| H -4.341875000 -3.196236000 2.435672000 |
| H -3.449088000 -1.677699000 2.186701000 |
| C -4.248033000 -2.980588000 -0.826942000 |
| H -5.197816000 -3.443827000 -0.529846000 |
| H -3.990326000 -3.360505000 -1.823285000 |
| H -4.404194000 -1.902515000 -0.916300000  **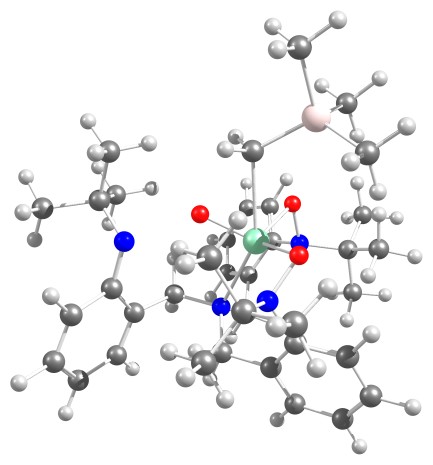** |
| **3** |
| Ti 0.650345000 -0.082276000 0.156969000 |
| Si 4.587145000 -1.606933000 -1.174942000 |
| O 1.339260000 1.013940000 -1.224300000 |
| O 2.142192000 -1.134448000 0.122883000 |
| O -0.683871000 -1.358397000 0.191817000 |
| N 0.013392000 1.369697000 -1.616867000 |
| N 0.850932000 0.693041000 1.934657000 |
| N -1.381794000 -2.304557000 -0.574802000 |
| N -2.069200000 1.110809000 0.705411000 |
| C -2.501037000 2.128147000 -0.263713000 |
| H -3.385838000 2.678514000 0.111882000 |
| H -2.820328000 1.605456000 -1.163853000 |
| C -1.421396000 3.130851000 -0.609153000 |
| C -1.608276000 4.480358000 -0.275013000 |
| H -2.536663000 4.770174000 0.211679000 |
| C -0.634460000 5.443340000 -0.531081000 |
| H -0.806895000 6.480193000 -0.255893000 |
| C 0.569965000 5.056506000 -1.119440000 |
| H 1.352289000 5.787365000 -1.305708000 |
| C 0.773432000 3.722771000 -1.467292000 |
| H 1.717235000 3.409251000 -1.895118000 |
| C -0.221148000 2.760035000 -1.245023000 |
| C -0.157422000 1.038184000 -3.097206000 |
| C 0.165150000 -0.452186000 -3.285885000 |
| H -0.114381000 -0.754563000 -4.301688000 |
| H -0.392303000 -1.079685000 -2.584697000 |
| H 1.233167000 -0.643055000 -3.158422000 |
| C 0.791864000 1.865910000 -3.985347000 |
| H 0.731897000 1.493672000 -5.014328000 |
| H 1.827754000 1.763458000 -3.649438000 |
| H 0.524735000 2.926684000 -4.000826000 |
| C -1.612450000 1.305082000 -3.508166000 |
| H -1.906151000 2.342429000 -3.317920000 |
| H -2.305419000 0.633956000 -2.994971000 |
| H -1.714003000 1.127610000 -4.584424000 |
| C -1.864279000 1.718752000 2.038133000 |
| H -2.809431000 2.161508000 2.408626000 |
| H -1.146051000 2.532359000 1.910050000 |
| C -1.352982000 0.718441000 3.045115000 |
| C -2.192248000 0.266671000 4.070809000 |
| H -3.188044000 0.696566000 4.158008000 |
| C -1.786123000 -0.722356000 4.965886000 |
| H -2.452889000 -1.052862000 5.757583000 |
| C -0.526472000 -1.301035000 4.807740000 |
| H -0.203746000 -2.102220000 5.468082000 |
| C 0.323363000 -0.858850000 3.794152000 |
| H 1.287356000 -1.333694000 3.658275000 |
| C -0.048288000 0.185014000 2.923213000 |
| C 2.036085000 1.461884000 2.463591000 |
| C 2.747733000 2.167780000 1.297870000 |
| H 2.070950000 2.841712000 0.767928000 |
| H 3.152697000 1.451655000 0.578151000 |
| H 3.582843000 2.759210000 1.691508000 |
| C 3.069539000 0.549533000 3.165779000 |
| H 3.991585000 1.113409000 3.354178000 |
| H 3.313436000 -0.309473000 2.533078000 |
| H 2.710929000 0.187412000 4.133084000 |
| C 1.562209000 2.544722000 3.456128000 |
| H 2.424997000 3.106687000 3.833286000 |
| H 1.044698000 2.109781000 4.316629000 |
| H 0.885195000 3.253979000 2.967440000 |
| C -3.045772000 -0.000084000 0.799714000 |
| H -3.998922000 0.386568000 1.209106000 |
| H -2.654087000 -0.704103000 1.529387000 |
| C -3.374490000 -0.715962000 -0.500142000 |
| C -4.564296000 -0.337964000 -1.148084000 |
| H -5.167303000 0.441244000 -0.687130000 |
| C -5.002638000 -0.918265000 -2.335506000 |
| H -5.931824000 -0.592626000 -2.794392000 |
| C -4.218333000 -1.907220000 -2.927695000 |
| H -4.513708000 -2.360590000 -3.870168000 |
| C -3.032439000 -2.298541000 -2.315016000 |
| H -2.397563000 -3.037226000 -2.790241000 |
| C -2.608515000 -1.756802000 -1.086152000 |
| C -1.460488000 -3.596011000 0.216114000 |
| C -0.017573000 -4.038314000 0.506271000 |
| H 0.535955000 -4.173322000 -0.428821000 |
| H -0.028434000 -4.994641000 1.041843000 |
| H 0.510955000 -3.303790000 1.116090000 |
| C -2.137711000 -4.688923000 -0.627824000 |
| H -3.187345000 -4.468654000 -0.839869000 |
| H -2.101923000 -5.629310000 -0.067120000 |
| H -1.612603000 -4.844103000 -1.576170000 |
| C -2.233881000 -3.416635000 1.536299000 |
| H -1.749167000 -2.677434000 2.178931000 |
| H -2.268022000 -4.366819000 2.081992000 |
| H -3.267787000 -3.104451000 1.349666000 |
| C 2.779497000 -2.071080000 -0.729226000 |
| H 2.784547000 -3.053322000 -0.229186000 |
| H 2.201603000 -2.212065000 -1.657733000 |
| C 5.285624000 -3.057028000 -2.184711000 |
| H 4.700714000 -3.231772000 -3.096317000 |
| H 5.284929000 -3.989166000 -1.606362000 |
| H 6.320547000 -2.861930000 -2.492058000 |
| C 4.630637000 -0.025595000 -2.214342000 |
| H 4.164446000 -0.186290000 -3.194378000 |
| H 5.660450000 0.311420000 -2.387247000 |
| H 4.082177000 0.783949000 -1.720781000 |
| C 5.605130000 -1.384299000 0.405910000 |
| H 6.657841000 -1.179435000 0.174338000 |
| H 5.571540000 -2.284912000 1.031263000 |
| H 5.226724000 -0.550226000 1.006801000 |

**
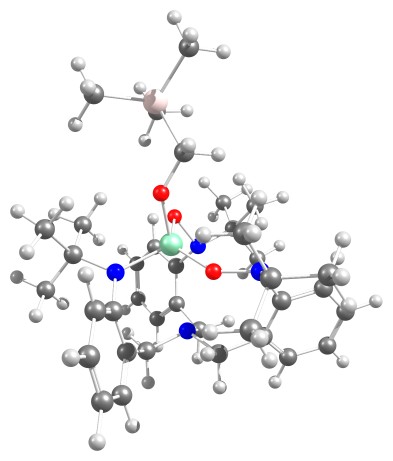
**

**4^+^**

| Ti -0.000897000 -0.140848000 -0.636858000 |
| --- |
| O -0.683686000 1.192853000 -1.763112000 |
| O 1.509554000 -0.541134000 -1.683654000 |
| N 0.109114000 0.049252000 1.616830000 |
| N -0.834545000 -1.894741000 -0.657717000 |
| N -1.490277000 1.487336000 -0.622455000 |
| N 2.145776000 0.222824000 -0.664683000 |
| C -0.293288000 -1.308559000 2.138784000 |
| H 0.472507000 -2.014169000 1.807548000 |
| H -0.267006000 -1.274343000 3.236562000 |
| C -1.657814000 -1.732087000 1.665573000 |
| C -2.695697000 -1.860645000 2.599727000 |
| H -2.487482000 -1.661482000 3.648312000 |
| C -3.974532000 -2.253756000 2.214657000 |
| H -4.760862000 -2.364357000 2.954763000 |
| C -4.229173000 -2.489907000 0.862122000 |
| H -5.223901000 -2.777749000 0.534144000 |
| C -3.213696000 -2.348453000 -0.078832000 |
| H -3.440346000 -2.517079000 -1.123100000 |
| C -1.896186000 -2.001502000 0.293694000 |
| C -0.451799000 -3.118377000 -1.498284000 |
| C 0.890874000 -3.657695000 -0.968084000 |
| H 1.215709000 -4.515952000 -1.567321000 |
| H 0.787302000 -3.996191000 0.069817000 |
| H 1.669789000 -2.899130000 -1.014299000 |
| C -1.456904000 -4.285876000 -1.420332000 |
| H -2.397661000 -4.081429000 -1.935962000 |
| H -1.676846000 -4.587243000 -0.392366000 |
| H -1.000873000 -5.144126000 -1.924342000 |
| C -0.330323000 -2.691717000 -2.972702000 |
| H 0.441076000 -1.932371000 -3.120927000 |
| H -1.283892000 -2.294658000 -3.337724000 |
| H -0.066763000 -3.557060000 -3.591112000 |
| C -0.846030000 1.090804000 2.142324000 |
| H -1.851978000 0.707020000 1.982745000 |
| H -0.695140000 1.179817000 3.226766000 |
| C -0.652665000 2.434831000 1.484624000 |
| C -0.145921000 3.516168000 2.219674000 |
| H 0.054575000 3.387314000 3.280309000 |
| C 0.112009000 4.743076000 1.610486000 |
| H 0.501997000 5.569371000 2.196901000 |
| C -0.112359000 4.894191000 0.240309000 |
| H 0.108930000 5.837259000 -0.250260000 |
| C -0.629418000 3.835018000 -0.504105000 |
| H -0.793000000 3.948534000 -1.568201000 |
| C -0.931639000 2.617396000 0.117856000 |
| C -2.977425000 1.604963000 -1.023097000 |
| C -3.814256000 1.441562000 0.253119000 |
| H -4.871944000 1.575795000 0.004706000 |
| H -3.560318000 2.199946000 1.001753000 |
| H -3.700697000 0.446706000 0.691680000 |
| C -3.292220000 2.960392000 -1.680413000 |
| H -4.331055000 2.935268000 -2.025565000 |
| H -2.661257000 3.136513000 -2.556851000 |
| H -3.197589000 3.802601000 -0.990341000 |
| C -3.291683000 0.502576000 -2.042210000 |
| H -2.944312000 -0.473221000 -1.709014000 |
| H -2.836049000 0.717973000 -3.010989000 |
| H -4.377216000 0.451872000 -2.175485000 |
| C 1.488429000 0.384411000 2.121683000 |
| H 1.690523000 1.414166000 1.826415000 |
| H 1.461076000 0.357471000 3.219623000 |
| C 2.555988000 -0.557780000 1.626489000 |
| C 3.243055000 -1.378259000 2.531653000 |
| H 3.023284000 -1.296046000 3.592955000 |
| C 4.189094000 -2.301182000 2.089866000 |
| H 4.712634000 -2.927239000 2.805798000 |
| C 4.437749000 -2.433817000 0.722557000 |
| H 5.149473000 -3.171159000 0.363960000 |
| C 3.770685000 -1.619814000 -0.191569000 |
| H 3.944901000 -1.740798000 -1.253011000 |
| C 2.857146000 -0.660549000 0.259367000 |
| C 2.998633000 1.368104000 -1.273421000 |
| C 3.424915000 2.294498000 -0.125979000 |
| H 4.075634000 3.075773000 -0.531517000 |
| H 3.992973000 1.765935000 0.645983000 |
| H 2.564929000 2.791664000 0.333890000 |
| C 4.238388000 0.804496000 -1.991426000 |
| H 4.741117000 1.629468000 -2.505699000 |
| H 3.954946000 0.066988000 -2.748457000 |
| H 4.962286000 0.360340000 -1.304565000 |
| C 2.165899000 2.147280000 -2.296045000 |
| H 1.294679000 2.618623000 -1.844816000 |
| H 1.829244000 1.507805000 -3.114685000 |
| H 2.799903000 2.935061000 -2.715244000 |

**
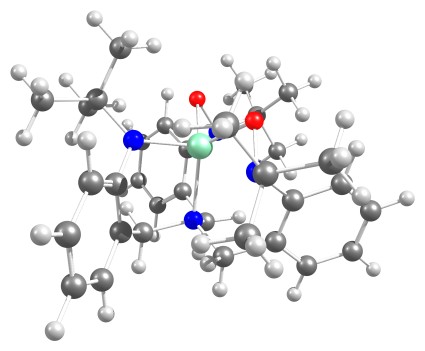
**

**NMO**

| C 0.114356000 1.228581000 -0.416909000 |
| --- |
| C -1.305301000 1.171167000 0.130934000 |
| C -1.305284000 -1.171172000 0.130933000 |
| C 0.114371000 -1.228578000 -0.416915000 |
| H -1.255446000 1.197914000 1.226183000 |
| H -1.883312000 2.024736000 -0.235107000 |
| H 0.114813000 1.249785000 -1.513111000 |
| H 0.662218000 2.084234000 -0.016853000 |
| H -1.255427000 -1.197929000 1.226183000 |
| H -1.883283000 -2.024750000 -0.235110000 |
| H 0.662242000 -2.084224000 -0.016854000 |
| H 0.114831000 -1.249780000 -1.513116000 |
| O -1.975507000 -0.000007000 -0.319396000 |
| N 0.868636000 0.000004000 0.068096000 |
| O 0.934756000 -0.000002000 1.424768000 |
| C 2.252935000 0.000007000 -0.499098000 |
| H 2.749859000 0.889197000 -0.110302000 |
| H 2.749869000 -0.889174000 -0.110294000 |
| H 2.242728000 0.000003000 -1.594934000  **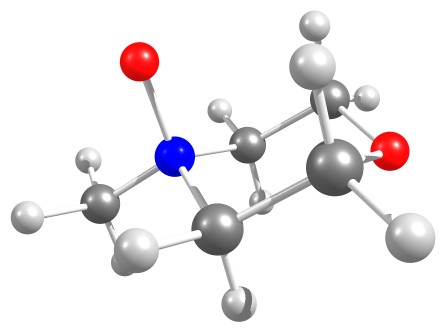** |

**4_NMO_^+^**

| Ti -0.034152000 0.067664000 -0.427855000 |
| --- |
| O 1.107906000 1.018912000 0.624167000 |
| O -1.120064000 1.698824000 -1.012506000 |
| O -1.437873000 -0.845800000 0.305418000 |
| N 2.357108000 1.073195000 1.302684000 |
| N 0.286767000 -0.374583000 -2.265028000 |
| N -2.114755000 -0.986851000 1.537589000 |
| N 1.215618000 -1.892830000 0.178371000 |
| C 2.546298000 -1.666058000 0.841950000 |
| H 3.034918000 -2.646329000 0.950464000 |
| H 2.335701000 -1.273025000 1.836431000 |
| C 3.490920000 -0.713637000 0.154653000 |
| C 4.523509000 -1.187867000 -0.661893000 |
| H 4.610279000 -2.257299000 -0.838476000 |
| C 5.466172000 -0.320867000 -1.217375000 |
| H 6.267111000 -0.711033000 -1.838030000 |
| C 5.377702000 1.044821000 -0.953274000 |
| H 6.102723000 1.733152000 -1.378060000 |
| C 4.351492000 1.539437000 -0.146173000 |
| H 4.289482000 2.604847000 0.030282000 |
| C 3.408624000 0.672829000 0.424112000 |
| C 2.420196000 2.253845000 2.210942000 |
| C 1.334411000 2.037018000 3.284953000 |
| H 1.368990000 2.849598000 4.018340000 |
| H 1.493544000 1.088651000 3.806357000 |
| H 0.333101000 2.012001000 2.849911000 |
| C 2.176690000 3.602640000 1.500934000 |
| H 2.030994000 4.396603000 2.241351000 |
| H 1.279959000 3.541759000 0.876734000 |
| H 3.009987000 3.903810000 0.860460000 |
| C 3.783963000 2.237421000 2.927659000 |
| H 4.622032000 2.463018000 2.265834000 |
| H 3.965041000 1.261707000 3.390230000 |
| H 3.773445000 2.991527000 3.721108000 |
| C 1.445702000 -2.662448000 -1.090526000 |
| H 1.836609000 -3.659246000 -0.835890000 |
| H 2.221428000 -2.129291000 -1.640925000 |
| C 0.213326000 -2.792658000 -1.948064000 |
| C -0.380033000 -4.038285000 -2.179101000 |
| H 0.059998000 -4.927238000 -1.733080000 |
| C -1.516036000 -4.154821000 -2.980665000 |
| H -1.958736000 -5.129828000 -3.160974000 |
| C -2.084237000 -3.006270000 -3.535919000 |
| H -2.980854000 -3.081446000 -4.144918000 |
| C -1.510014000 -1.756406000 -3.307070000 |
| H -1.973383000 -0.867919000 -3.718240000 |
| C -0.339423000 -1.631514000 -2.535570000 |
| C 0.958977000 0.359017000 -3.392375000 |
| C 1.699222000 1.570639000 -2.805835000 |
| H 2.449041000 1.263049000 -2.073191000 |
| H 1.003037000 2.265675000 -2.326884000 |
| H 2.217036000 2.108249000 -3.607624000 |
| C -0.069595000 0.868645000 -4.426167000 |
| H 0.429358000 1.539794000 -5.134419000 |
| H -0.871655000 1.426679000 -3.931832000 |
| H -0.508374000 0.053994000 -5.007900000 |
| C 1.976153000 -0.559992000 -4.097815000 |
| H 2.411443000 -0.040703000 -4.958901000 |
| H 1.502367000 -1.475743000 -4.466068000 |
| H 2.796053000 -0.832528000 -3.424797000 |
| C 0.384969000 -2.761374000 1.088945000 |
| H 0.972702000 -3.665901000 1.307447000 |
| H -0.480498000 -3.070808000 0.511032000 |
| C -0.034544000 -2.142212000 2.408242000 |
| C 0.745165000 -2.423283000 3.542702000 |
| H 1.614175000 -3.066042000 3.425968000 |
| C 0.434925000 -1.928350000 4.807464000 |
| H 1.060640000 -2.177786000 5.658978000 |
| C -0.682076000 -1.108214000 4.959971000 |
| H -0.935391000 -0.693413000 5.931239000 |
| C -1.472930000 -0.811280000 3.852840000 |
| H -2.332522000 -0.158901000 3.958837000 |
| C -1.190093000 -1.343214000 2.585017000 |
| C -3.348409000 -1.852221000 1.341607000 |
| C -4.199432000 -1.192053000 0.245904000 |
| H -4.495353000 -0.180222000 0.547129000 |
| H -5.114812000 -1.772892000 0.093218000 |
| H -3.661280000 -1.145210000 -0.703895000 |
| C -4.152791000 -1.856957000 2.653002000 |
| H -3.626420000 -2.366369000 3.464029000 |
| H -5.093893000 -2.389278000 2.482280000 |
| H -4.396828000 -0.840047000 2.977555000 |
| C -3.019271000 -3.300675000 0.938894000 |
| H -2.519427000 -3.344524000 -0.032743000 |
| H -3.946592000 -3.878124000 0.857645000 |
| H -2.393993000 -3.792519000 1.691463000 |
| C -2.126755000 2.341557000 1.050624000 |
| H -1.137077000 2.505400000 1.473399000 |
| H -2.845725000 3.032142000 1.493581000 |
| H -2.433672000 1.306630000 1.212836000 |
| C -1.529243000 3.988155000 -0.723789000 |
| C -3.355797000 2.394771000 -1.126312000 |
| C -2.596408000 5.047201000 -0.442741000 |
| H -1.274623000 3.971721000 -1.784220000 |
| H -0.620696000 4.137253000 -0.137932000 |
| C -4.334020000 3.532357000 -0.830169000 |
| H -3.103897000 2.371962000 -2.187285000 |
| H -3.734813000 1.417920000 -0.825254000 |
| H -2.219417000 6.017316000 -0.777347000 |
| H -2.814905000 5.126880000 0.633984000 |
| H -5.223738000 3.395544000 -1.450503000 |
| H -4.656975000 3.526295000 0.223015000 |
| N -2.022761000 2.584092000 -0.425178000 |
| O -3.777721000 4.787133000 -1.176517000  **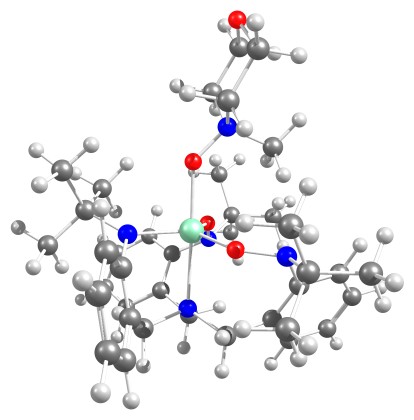** |

**TS2^+^**

| Ti -0.142328000 -0.195666000 0.112980000 |
| --- |
| O 1.139004000 -0.465341000 -1.115668000 |
| O -1.557575000 -1.388520000 -0.176663000 |
| O -1.024501000 1.394615000 0.069928000 |
| N 2.536013000 -0.425228000 -1.398870000 |
| N -0.532496000 -1.575694000 1.548760000 |
| N -1.068377000 2.654812000 -0.548953000 |
| N 1.368423000 0.808685000 1.568881000 |
| C 2.818729000 0.654034000 1.183480000 |
| H 3.422294000 1.076855000 2.000081000 |
| H 2.964821000 1.267826000 0.294712000 |
| C 3.312151000 -0.736082000 0.870110000 |
| C 3.995186000 -1.479139000 1.839890000 |
| H 4.105364000 -1.066825000 2.840178000 |
| C 4.579351000 -2.710316000 1.537864000 |
| H 5.115091000 -3.262937000 2.303607000 |
| C 4.485052000 -3.208442000 0.240069000 |
| H 4.936256000 -4.162924000 -0.015060000 |
| C 3.802792000 -2.488085000 -0.742318000 |
| H 3.728126000 -2.903607000 -1.737527000 |
| C 3.217880000 -1.248651000 -0.448503000 |
| C 2.766059000 -0.450854000 -2.875275000 |
| C 2.118184000 0.833227000 -3.432739000 |
| H 2.326160000 0.915131000 -4.504611000 |
| H 2.520389000 1.717856000 -2.930489000 |
| H 1.033770000 0.829743000 -3.296727000 |
| C 2.154220000 -1.674209000 -3.590704000 |
| H 2.185272000 -1.520920000 -4.674599000 |
| H 1.108371000 -1.807006000 -3.296573000 |
| H 2.684931000 -2.606381000 -3.380992000 |
| C 4.284203000 -0.355415000 -3.116487000 |
| H 4.826399000 -1.252722000 -2.811315000 |
| H 4.707049000 0.500883000 -2.580987000 |
| H 4.461353000 -0.206835000 -4.186357000 |
| C 1.143766000 0.212536000 2.928592000 |
| H 1.667873000 0.824149000 3.677055000 |
| H 1.609302000 -0.772759000 2.920367000 |
| C -0.320613000 0.096668000 3.288352000 |
| C -0.872290000 0.862968000 4.320142000 |
| H -0.233286000 1.547386000 4.872769000 |
| C -2.223858000 0.756057000 4.651123000 |
| H -2.634761000 1.356717000 5.456943000 |
| C -3.045403000 -0.116014000 3.932164000 |
| H -4.102776000 -0.188842000 4.169796000 |
| C -2.514667000 -0.894169000 2.905822000 |
| H -3.155326000 -1.548060000 2.328366000 |
| C -1.146829000 -0.818210000 2.588371000 |
| C -0.377803000 -3.063359000 1.714690000 |
| C 0.374454000 -3.621709000 0.499241000 |
| H 1.342741000 -3.132095000 0.370769000 |
| H -0.205251000 -3.495105000 -0.416629000 |
| H 0.558088000 -4.691074000 0.648072000 |
| C -1.732697000 -3.786411000 1.855927000 |
| H -1.560234000 -4.868073000 1.853439000 |
| H -2.393553000 -3.539898000 1.021439000 |
| H -2.236060000 -3.541795000 2.795295000 |
| C 0.459560000 -3.329085000 2.986734000 |
| H 0.513573000 -4.409576000 3.158239000 |
| H 0.007338000 -2.874433000 3.873859000 |
| H 1.484054000 -2.960558000 2.876565000 |
| C 1.070297000 2.291699000 1.673141000 |
| H 1.755596000 2.702432000 2.429195000 |
| H 0.062364000 2.371685000 2.071132000 |
| C 1.233304000 3.105964000 0.404687000 |
| C 2.443651000 3.799315000 0.229206000 |
| H 3.203025000 3.719034000 1.003375000 |
| C 2.696576000 4.595534000 -0.884703000 |
| H 3.642740000 5.119931000 -0.976478000 |
| C 1.723770000 4.699901000 -1.878709000 |
| H 1.904544000 5.295343000 -2.768812000 |
| C 0.515846000 4.023580000 -1.732805000 |
| H -0.236139000 4.076707000 -2.511520000 |
| C 0.236403000 3.257832000 -0.589607000 |
| C -2.225938000 3.460996000 0.009574000 |
| C -3.498686000 2.619821000 -0.169411000 |
| H -3.656909000 2.380026000 -1.226860000 |
| H -4.365472000 3.190421000 0.179731000 |
| H -3.446225000 1.691509000 0.404496000 |
| C -2.367823000 4.749986000 -0.817786000 |
| H -1.515387000 5.422671000 -0.693414000 |
| H -3.262574000 5.283186000 -0.480987000 |
| H -2.489162000 4.529034000 -1.882960000 |
| C -2.044903000 3.822521000 1.495083000 |
| H -2.018355000 2.928043000 2.124436000 |
| H -2.888019000 4.435927000 1.830729000 |
| H -1.131862000 4.406033000 1.654988000 |
| C -2.018364000 -0.208443000 -2.738938000 |
| H -0.968931000 -0.408663000 -2.966406000 |
| H -2.571210000 -0.132296000 -3.685273000 |
| H -2.092284000 0.742279000 -2.207798000 |
| C -2.346845000 -2.642631000 -2.436677000 |
| C -3.916314000 -1.102091000 -1.417320000 |
| C -3.415321000 -2.970757000 -3.491796000 |
| H -2.439283000 -3.341694000 -1.601596000 |
| H -1.337546000 -2.723083000 -2.850491000 |
| C -4.932241000 -1.482381000 -2.505374000 |
| H -4.048859000 -1.758977000 -0.553600000 |
| H -4.041516000 -0.067557000 -1.091368000 |
| H -3.324826000 -4.016916000 -3.796806000 |
| H -3.291428000 -2.341201000 -4.388118000 |
| H -5.944948000 -1.447435000 -2.094424000 |
| H -4.883571000 -0.780249000 -3.353779000 |
| N -2.530842000 -1.286748000 -1.886670000 |
| O -4.714930000 -2.811201000 -2.949517000 |

**
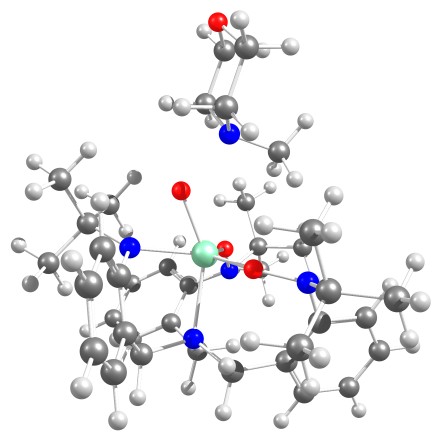
**

**1^+^**

| Ti 0.001664000 -0.001588000 -0.829315000 |
| --- |
| O 1.048597000 1.234887000 -1.753574000 |
| O 0.552791000 -1.528391000 -1.749791000 |
| O -1.595736000 0.283975000 -1.749518000 |
| N 0.001028000 0.000057000 1.542158000 |
| N 0.756150000 2.092372000 -0.648277000 |
| N 1.437541000 -1.705183000 -0.642144000 |
| N -2.192572000 -0.390518000 -0.640783000 |
| C 0.396861000 1.352654000 2.084789000 |
| H -0.397389000 2.046834000 1.815621000 |
| H 0.418609000 1.285663000 3.181612000 |
| C 1.736350000 1.826680000 1.583587000 |
| C 2.831506000 1.907301000 2.455540000 |
| H 2.689109000 1.674180000 3.507771000 |
| C 4.095242000 2.267175000 1.990798000 |
| H 4.931623000 2.326646000 2.680486000 |
| C 4.281505000 2.525908000 0.631255000 |
| H 5.266942000 2.778834000 0.251778000 |
| C 3.200086000 2.465965000 -0.246991000 |
| H 3.348984000 2.650828000 -1.302999000 |
| C 1.921716000 2.153144000 0.230896000 |
| C 0.216284000 3.458336000 -1.130796000 |
| C -0.318283000 4.204785000 0.100323000 |
| H -0.659732000 5.197131000 -0.211180000 |
| H 0.451598000 4.351638000 0.864770000 |
| H -1.174948000 3.687828000 0.544808000 |
| C 1.317052000 4.288566000 -1.816047000 |
| H 1.775735000 3.735829000 -2.641735000 |
| H 2.094542000 4.614905000 -1.121468000 |
| H 0.858123000 5.188669000 -2.236866000 |
| C -0.916832000 3.235752000 -2.138708000 |
| H -1.754170000 2.694459000 -1.701531000 |
| H -0.571214000 2.688975000 -3.018894000 |
| H -1.276984000 4.216503000 -2.465684000 |
| C 0.974109000 -1.017302000 2.087051000 |
| H 1.972067000 -0.674726000 1.818111000 |
| H 0.905374000 -1.000859000 3.183892000 |
| C 0.713271000 -2.415307000 1.588601000 |
| C 0.233117000 -3.401318000 2.461963000 |
| H 0.101647000 -3.159063000 3.513559000 |
| C -0.086744000 -4.676940000 1.999892000 |
| H -0.454595000 -5.429155000 2.690971000 |
| C 0.046056000 -4.971140000 0.641326000 |
| H -0.227085000 -5.952009000 0.263996000 |
| C 0.535897000 -4.006739000 -0.238908000 |
| H 0.621238000 -4.230241000 -1.294481000 |
| C 0.903643000 -2.742385000 0.236623000 |
| C 2.888997000 -1.926093000 -1.124014000 |
| C 3.798978000 -1.893451000 0.112392000 |
| H 4.826870000 -2.098841000 -0.202912000 |
| H 3.525796000 -2.654670000 0.849872000 |
| H 3.792947000 -0.908199000 0.589897000 |
| C 3.037314000 -3.271175000 -1.858621000 |
| H 4.041750000 -3.319702000 -2.290729000 |
| H 2.320062000 -3.354739000 -2.680973000 |
| H 2.929215000 -4.131164000 -1.193379000 |
| C 3.282503000 -0.801310000 -2.087184000 |
| H 3.248422000 0.174740000 -1.605579000 |
| H 2.636392000 -0.777850000 -2.967529000 |
| H 4.309202000 -0.982315000 -2.421399000 |
| C -1.366680000 -0.331351000 2.088767000 |
| H -1.573003000 -1.366400000 1.821608000 |
| H -1.317188000 -0.278897000 3.185633000 |
| C -2.445449000 0.594607000 1.589241000 |
| C -3.058200000 1.505220000 2.461456000 |
| H -2.782736000 1.498633000 3.513042000 |
| C -4.000466000 2.421695000 1.997616000 |
| H -4.467086000 3.118123000 2.687535000 |
| C -4.319935000 2.453638000 0.638581000 |
| H -5.030484000 3.182368000 0.260195000 |
| C -3.731182000 1.545193000 -0.240565000 |
| H -3.967251000 1.582301000 -1.296215000 |
| C -2.822780000 0.592633000 0.236880000 |
| C -3.109137000 -1.538962000 -1.118560000 |
| C -3.510466000 -2.356688000 0.118013000 |
| H -4.207840000 -3.141863000 -0.190661000 |
| H -4.019443000 -1.747714000 0.871877000 |
| H -2.644655000 -2.847497000 0.574212000 |
| C -4.363880000 -0.998084000 -1.828050000 |
| H -4.910647000 -1.844604000 -2.255342000 |
| H -4.095493000 -0.329072000 -2.651407000 |
| H -5.045489000 -0.481252000 -1.148216000 |
| C -2.342571000 -2.427213000 -2.104114000 |
| H -1.455348000 -2.867569000 -1.652019000 |
| H -2.038815000 -1.872643000 -2.994858000 |
| H -3.006807000 -3.239086000 -2.417621000  **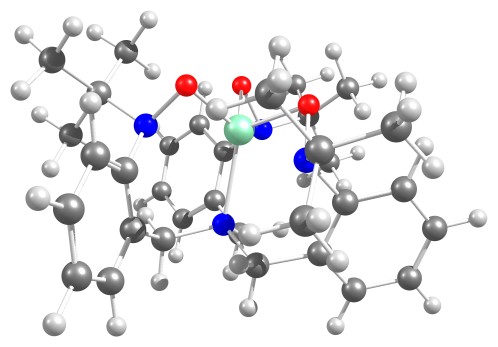** |

**NMM**

| C -0.286634000 1.199198000 0.187326000 |
| --- |
| C 1.191361000 1.169320000 -0.198387000 |
| C 1.191362000 -1.169320000 -0.198385000 |
| C -0.286634000 -1.199198000 0.187325000 |
| H 1.283406000 1.216513000 -1.296143000 |
| H 1.723193000 2.020950000 0.236790000 |
| H -0.370990000 1.283687000 1.289961000 |
| H -0.765193000 2.084039000 -0.251526000 |
| H 1.283407000 -1.216515000 -1.296141000 |
| H 1.723194000 -2.020949000 0.236793000 |
| H -0.765191000 -2.084039000 -0.251531000 |
| H -0.370995000 -1.283689000 1.289959000 |
| O 1.830114000 0.000000000 0.295023000 |
| N -0.954543000 0.000000000 -0.316024000 |
| C -2.377590000 0.000000000 -0.019711000 |
| H -2.846111000 0.887193000 -0.460646000 |
| H -2.846109000 -0.887194000 -0.460647000 |
| H -2.598902000 -0.000001000 1.066111000  **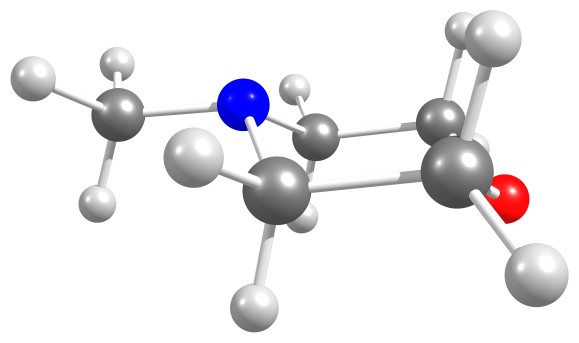** |

**H_2_NO^−^**

| N 0.072973000 -0.623499000 0.000000000 |
| --- |
| O 0.072973000 0.786354000 0.000000000 |
| H -0.547300000 -0.963169000 0.798125000 |
| H -0.547300000 -0.963169000 -0.798125000  **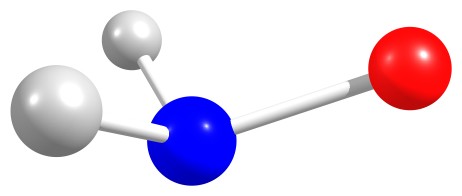** |

**HOO^–^**

| O 0.056557000 -0.710513000 0.000000000 |
| --- |
| H -0.904909000 -0.844696000 0.000000000 |
| O 0.056557000 0.816100000 0.000000000  **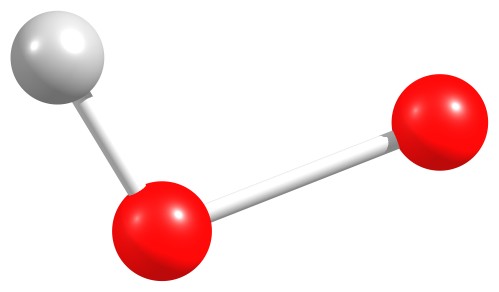** |

**References.**

1. J. A. Bogart, C. A. Lippincott, P. J. Carroll, C. H. Booth and E. J. Schelter, *Chem-Eur J*, 2015, **21**, 17850-17859.

2. M. A. Boreen, J. A. Bogart, P. J. Carroll and E. J. Schelter, *Inorganic Chemistry*, 2015, **54**, 9588-9593.

3. Bruker APEX 2, v2014.11-0; Bruker AXS Inc.: Madison, Wisconsin, 2012.

4. Bruker APEX 3, v2016.1-0; Bruker AXS Inc.: Madison, Wisconsin, 2015.

5. Bruker SAINT, v8.37a; Bruker AXS Inc.: Madison, Wisconsin, 2012.

6. G. M. Sheldrick, *Acta Crystallographica Section A: Foundations of Crystallography*, 2015, **71**, 3–8.

7. Bruker SADABS, v2014/5; Bruker AXS Inc.: Madison, Wisconsin, 2001

8. G. M. Sheldrick, *Acta Crystallographica Section C: Crystal Structure Communications*, 2015, **71**, 3–8.

9. M. J. Frisch, G. W. Trucks, H. B. Schlegel, G. E. Scuseria, M. A. Robb, J. R. Cheeseman, G. Scalmani, V. Barone, B. Mennucci, G. A. Petersson, H. Nakatsuji, M. Caricato, X. Li, H. P. Hratchian, A. F. Izmaylov, J. Bloino, G. Zheng, J. L. Sonnenberg, M. Hada, M. Ehara, K. Toyota, R. Fukuda, J. Hasegawa, M. Ishida, T. Nakajima, Y. Honda, O. Kitao, H. Nakai, T. Vreven, J. A. Montgomery Jr., J. E. Peralta, F. Ogliaro, M. Bearpark, J. J. Heyd, E. Brothers, K. N. Kudin, V. N. Staroverov, R. Kobayashi, J. Normand, K. Raghavachari, A. Rendell, J. C. Burant, S. S. Iyengar, J. Tomasi, M. Cossi, N. Rega, N. J. Millam, M. Klene, J. E. Knox, J. B. Cross, V. Bakken, C. Adamo, J. Jaramillo, R. Gomperts, R. E. Stratmann, O. Yazyev, A. J. Austin, R. Cammi, C. Pomelli, J. W. Ochterski, R. L. Martin, K. Morokuma, V. G. Zakrzewski, V. Voth, J. Cioslowski and D. J. Fox, *Journal*, 2009, **Revision D.01**.

10. A. D. Becke, *Journal of Chemical Physics*, 1993, **98**, 5648-5652.

11. C. Lee, W. Yang and R. G. Parr, *Physical Review B*, 1988, **37**, 785-789.

12. S. H. Vosko, L. Wilk and M. Nusair, *Canadian Journal of Physics*, 1980, **58**, 1200-1211.

13. P. J. Stephens, F. J. Devlin, C. F. Chabalowski and M. J. Frisch, *Journal of Physical Chemistry*, 1994, **98**, 11623-11627.

14. S. Grimme, J. Antony, S. Ehrlich and H. Krieg, *Journal of Chemical Physics*, 2010, **132**, 154104.

15. W. J. Hehre, R. Ditchfield and J. A. Pople, *Journal of Chemical Physics*, 1972, **56**, 2257-2261.

16. F. Weigend, *Physical Chemistry Chemical Physics*, 2006, **8**, 1057-1065.

17. F. Weigend and R. Ahlrichs, *Physical Chemistry Chemical Physics*, 2005, **7**, 3297-3305.
